# Extracellular phosphorylation of a receptor tyrosine kinase controls synaptic localization of NMDA receptors and regulates pathological pain

**DOI:** 10.1371/journal.pbio.2002457

**Published:** 2017-07-18

**Authors:** Kenji Hanamura, Halley R. Washburn, Sean I. Sheffler-Collins, Nan L. Xia, Nathan Henderson, Dipti V. Tillu, Shayne Hassler, Daniel S. Spellman, Guoan Zhang, Thomas A. Neubert, Theodore J. Price, Matthew B. Dalva

**Affiliations:** 1 Department of Neuroscience and Farber Institute for Neurosciences, Thomas Jefferson University, Jefferson Hospital for Neuroscience, Philadelphia, Pennsylvania, United States of America; 2 Department of Neurobiology and Behavior, Gunma University Graduate School of Medicine, Maebashi City, Gunma, Japan; 3 Neuroscience Graduate Group, University of Pennsylvania School of Medicine, Philadelphia, Pennsylvania, United States of America; 4 Department of Pharmacology, University of Arizona College of Medicine, Tucson, Arizona, United States of America; 5 School of Behavioral and Brain Sciences, University of Texas at Dallas, Richardson, Texas, United States of America; 6 Department of Cell Biology and Kimmel Center for Biology and Medicine at the Skirball Institute, New York University School of Medicine, New York, New York, United States of America; University of California, San Francisco, United States of America

## Abstract

Extracellular phosphorylation of proteins was suggested in the late 1800s when it was demonstrated that casein contains phosphate. More recently, extracellular kinases that phosphorylate extracellular serine, threonine, and tyrosine residues of numerous proteins have been identified. However, the functional significance of extracellular phosphorylation of specific residues in the nervous system is poorly understood. Here we show that synaptic accumulation of GluN2B-containing N-methyl-D-aspartate receptors (NMDARs) and pathological pain are controlled by ephrin-B-induced extracellular phosphorylation of a single tyrosine (p*Y504) in a highly conserved region of the fibronectin type III (FN3) domain of the receptor tyrosine kinase EphB2. Ligand-dependent Y504 phosphorylation modulates the EphB-NMDAR interaction in cortical and spinal cord neurons. Furthermore, Y504 phosphorylation enhances NMDAR localization and injury-induced pain behavior. By mediating inducible extracellular interactions that are capable of modulating animal behavior, extracellular tyrosine phosphorylation of EphBs may represent a previously unknown class of mechanism mediating protein interaction and function.

## Author summary

The activity of proteins can be finely and reversibly tuned by post-translational modifications. The attachment of phosphate groups to tyrosine residues is one of such modifications. While the existence of extracellular phosphoproteins has been known, the functional significance of extracellular phosphorylation is poorly understood. Here we describe a single extracellular tyrosine whose inducible phosphorylation may represent an archetype for a new class of mechanism mediating protein—protein interaction and regulating protein function. We show that the interaction between EphB2—which occurs upon receptor activation by its ligand ephrin-B—and the N-methyl-D-aspartate receptor (NMDAR) depends on extracellular phosphorylation of EphB2. This interaction regulates the localization of the NMDA receptor to synaptic sites in neurons. In vivo, EphB2 is phosphorylated in response to injury, and the subsequent up-regulation of the interaction between EphB2 and NMDA receptors enhances injury-induced pain behavior and mechanical hypersensitivity in mice. Importantly, our study defines a specific extracellular phosphorylation event as a mechanism driving protein interaction and suggests that extracellular phosphorylation of proteins is an underappreciated mechanism contributing to the development and function of the nervous system and synapse.

## Introduction

Modification of protein function by phosphorylation controls many aspects of cellular function and signaling [1]. Interestingly, the first evidence for phosphoproteins came from the observation that the secreted milk protein, casein, contained phosphate, suggesting that phosphorylation can occur in the extracellular space [2, 3]. Recently, protein kinases that mediate the selective phosphorylation of extracellular serine, threonine, and tyrosine amino acids have been identified. For example, extracellular phosphorylation of serine and threonine residues can be mediated by Fam20C [4, 5] and phosphorylation of extracellular tyrosine residues can be accomplished by vertebrate lonesome kinase (VLK or PKDCC), an essential gene expressed throughout the body, including the nervous system [6, 7]. Yet despite identification of these kinases, the functional significance of extracellular phosphorylation and whether extracellular phosphorylation of proteins inducibly modulates their function remains largely unexplored.

At excitatory synapses, glutamate receptors must be recruited and stabilized at synaptic sites. Of particular importance are interactions that maintain the proper localization of N-methyl-D-aspartate receptors (NMDARs), glutamate receptors that are essential for synaptic plasticity and development [8]. The synaptic localization, function, and signaling of NMDARs are regulated by intracellular scaffolding proteins such as PSD-95 [9], extracellular interacting proteins such as neuroligin-1 [10], and the EphB receptor tyrosine kinases (RTKs) [11]. While the mechanisms mediating intracellular interactions are well understood, the mechanisms mediating extracellular protein—protein interactions are not.

The direct extracellular interaction between the EphB receptor tyrosine kinase and the NMDAR appears to play important roles in the localization, function, and signaling of NMDARs [11]. The EphB family of RTKs consists of 5 members that bind to transmembrane ephrin-B ligands. EphB1–3 are essential for formation of up to 40% of excitatory synapses in the developing hippocampus and cortex, while in the mature brain EphBs are required for normal levels of synaptic NMDARs [12–14]. Ephrin-B binding to EphBs controls the localization and function of synaptic NMDARs by inducing a direct extracellular domain—dependent interaction with the NMDAR [11, 14–17]. While in vitro binding assays indicate that EphBs bind the GluN1 subunit of the NMDAR via a direct extracellular interaction [11, 18], the domain and molecular mechanism mediating the interaction between these 2 proteins remain undefined.

Underscoring the importance of the EphB—NMDAR interaction, the EphB—NMDAR interaction has been linked to a number of human diseases that are associated with NMDAR dysfunction. The pathological disruption of the ability of the EphB and NMDAR to interact has been linked to NMDAR dysfunction in Alzheimer disease [19, 20] and in anti-NMDAR encephalitis [18, 21]. A common feature of these findings is the disruption of the ability of EphBs to interact biochemically with the NMDAR and a rescue of the defects associated with the disease state by restoring the EphB—NMDAR interaction. The EphB-dependent enhancement of NMDAR activity associated with the EphB—NMDAR interaction is also linked to disease. EphB-dependent enhancement of NMDAR function plays a key role in sensitization of nociception, leading to chronic neuropathic and malignancy-induced pain through an unknown mechanism [22–25]. Because the interaction between EphBs and the NMDAR occurs in the extracellular space, it is thought to be a promising drug target [19]. However, despite the apparent importance of the EphB—NMDAR interaction, the molecular mechanisms controlling direct extracellular interaction between these proteins are unknown.

Here we show that the EphB2 receptor tyrosine kinase undergoes ephrin-B ligand-induced extracellular phosphorylation of tyrosine residues (Y481 and Y504). Sequence analysis indicates that 1 of these amino acids, Y504, is widely conserved in fibronectin type III (FN3) domains of Eph proteins across phylogeny and is present in all Eph proteins known to interact with the NMDAR. In cortical and spinal cord neurons, ephrin-B—dependent induction of the EphB—NMDAR interaction is mediated by extracellular phosphorylation of Y504 on EphB2. The charge of Y504 is both necessary and sufficient for EphB—NMDAR interaction and regulates the amount of NMDARs found at synaptic sites. Virally mediated spinal cord expression of EphB2 or a phosphomimetic EphB mutant that induces the EphB—NMDAR interaction increases NMDAR levels in the dorsal horn of the spinal cord and results in mechanical hypersensitivity. Intrathecal injections of a membrane-impermeable ectokinase inhibitor that blocks the EphB—NMDAR interaction in cortical and spinal cord neurons reduces long-term hypersensitivity induced by EphB2 wild type (WT) but not pathological pain induced by injection of a phosphomimetic EphB mutant. These findings suggest that extracellular phosphorylation of EphB2 regulates NMDAR synaptic localization and function. Together the data suggest extracellular phosphorylation as a novel, dynamic mechanism that regulates protein—protein interactions at synapses to drive assembly of macromolecular complexes.

## Results

### Extracellular phosphorylation of the EphB2 RTK

**Fig 1:**
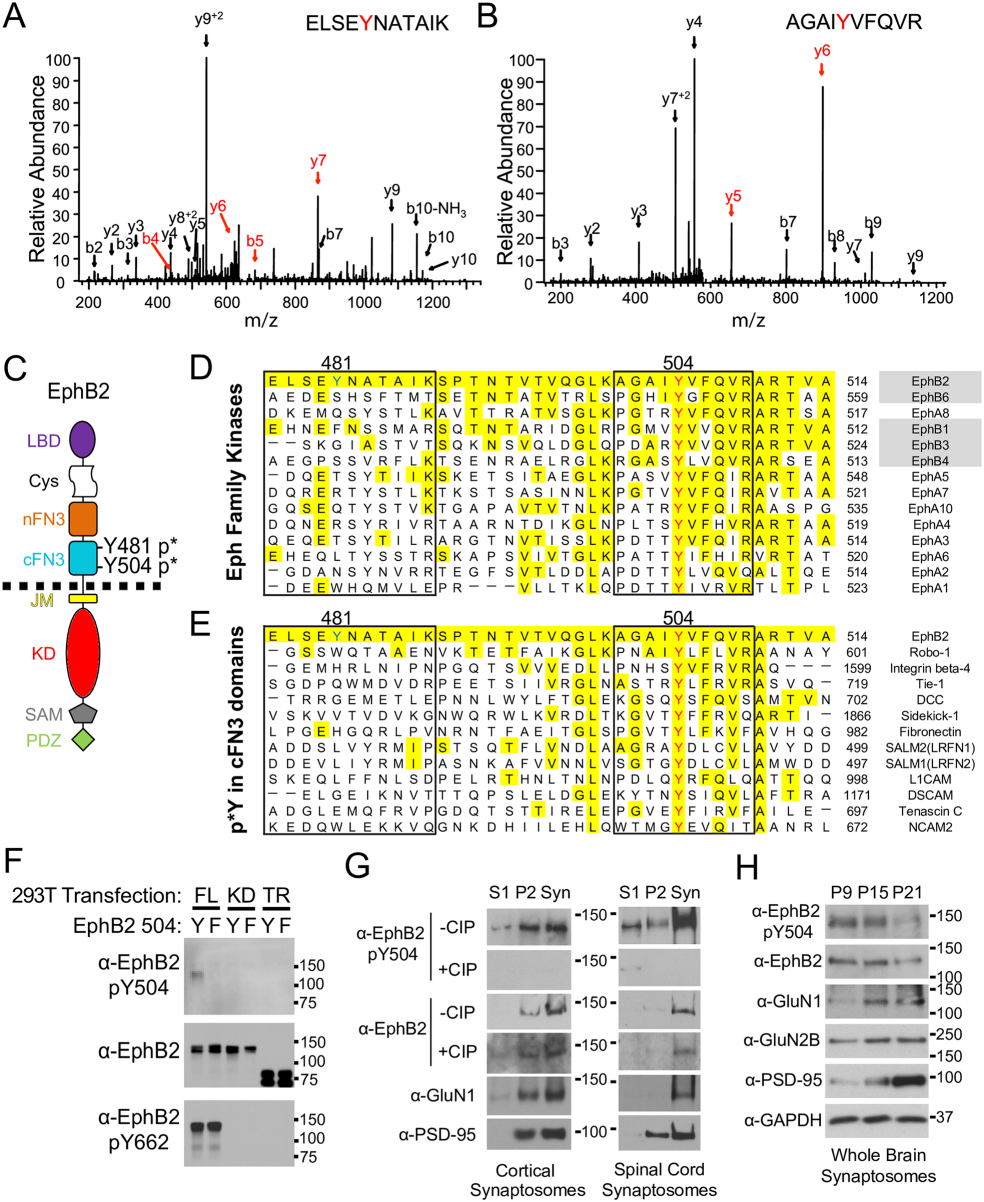
Identification of phosphorylated tyrosine in C-terminal fibronectin type III (FN3) (cFN3) of EphB2 extracellular region. (A and B) Tandem mass spectrometry (MS/MS) spectra of peptides (A) ELSEYNATAIK and (B) AGAIYVFQR are shown. Fragments critical for localization of phosphorylation sites are labeled in red. (C) Schematic of the known functional domains of EphB2 receptor. LBD, ligand-binding domain (purple); Cys, cysteine-rich domain (white); FN3, fibronectin type III repeat domain (orange [N-terminal FN3 (nFN3)]and blue [cFN3]); TM, transmembrane domain; JM, juxtamembrane domain (yellow); TK, tyrosine kinase domain (red), SAM (grey), sterile α-motif; PDZ, PSD-95/DLG1/ZO-1 domain (green). (D) Alignment of cFN3 domains of EphB2 with Eph family proteins (Uniprot database) using ClustalW2 software. EphB2 Y504 (red) corresponds to a conserved tyrosine residue, whereas Y481 (blue) is only conserved in mammals (S1E Fig, grey). Yellow indicates identical amino acids with mouse EphB2. (E) Alignment of cFN3 domain of EphB2 with other FN3-containing molecules. Y504 and neighboring residues in identified peptide (red) are well conserved amongst Eph family proteins (55.4% identical amino acids) or FN3-containing molecules (45.5% identical amino acids), whereas Y481 and neighboring residues in identified peptide (blue) are less well conserved amongst species (19.6% and 11.6% identical amino acids for Eph family proteins and FN3-containing molecules, respectively). (F) Top blot shows HEK293T lysates probed with a phospho-specific antibody generated against EphB2 Y504 (α-EphB2 p*Y504). Middle blot shows same lysates probed for EphB2. Bottom blot shows lysates probed for EphB2 p*Y662 (EphB2 kinase activity). Lanes were loaded with lysates of HEK293T cells transfected with full-length (FL) EphB2 wild type (Y) or Y504F (F), kinase-dead (KD) EphB2 wild type (Y) or Y504F (F), and truncated (TR) EphB2 wild type (Y) or Y504F (F). (G) Left blots show synaptosome lysates prepared from wild-type (WT) CD1 mouse brain and right blots show synaptosome lysates prepared from the spinal cord. Gels were loaded with nonsynaptic (S1), crude synaptosomal (P2), and synaptosomal (Syn) fractions. The top 2 blots were probed with α-EphB2 p*Y504 antibody before (upper) and after (lower) incubation with calf intestinal alkaline phosphatase (CIP) overnight to remove phosphate groups. The third and fourth blots were probed with α-EphB2 before and after CIP treatment. The fifth blot was probed with α-GluN1. The bottom blot was probed with α-PSD-95. (H) Blots show synaptosome lysates prepared from WT CD1 mouse brain at P9, P15, or P21. The top blot was probed with α-EphB2 p8Y504 antibody, the second blot was probed with α-EphB2, the third blot was probed with α-GluN1, the fourth blot was probed with α-GluN2B, and the fifth blot was probed with α-PSD-95. The bottom blot was probed with α-GAPDH as a loading control.

EphB receptors interact directly with NMDA-type glutamate receptors through an undefined region of their extracellular domain [11]. The extracellular domain of the EphB receptor consists of a globular domain required for ephrin-B binding, a cysteine-rich domain, and 2 FN3 repeat domains of unknown function (Fig 1C). To study whether the extracellular region of EphB2 undergoes post-translational modification, we took an unbiased mass spectrometry—based approach: liquid chromatography tandem mass spectrometry (LC-MS/MS) in combination with receptor immunoprecipitation (IP) and phosphopeptide mapping (S1A–S1D Fig). FLAG-tagged EphB2 was expressed in the neuroblastoma cell line NG108, treated with either ephrin-B1 or control reagents, and immunoprecipitated. After enrichment of phosphopeptides using TiO_2_, LC-MS/MS identified the known tyrosine phosphopeptides in the juxtamembrane and kinase domains (S1 Table) of EphB2 and 2 tyrosine phosphopeptides (ELSE**Y**NATAIK [Y481] and AGAI**Y**VFQVR [Y504]) from the extracellular portion of EphB2 (Fig 1A–1C). Each extracellular peptide was identified in 4 independent experiments, with Mascot scores of 34 and 63, respectively, and definable separation from the next peptide assigned to that spectrum. Manual inspection of the tandem mass spectrometry (MS/MS) spectrum confirmed that the majority of ion signals present are accounted for by the assigned amino acid sequence and ions critical to localization of the site of phosphorylation are present. The 2 phosphopeptides in the extracellular region of EphB2 were both found in the C-terminal FN3 (cFN3) domain (see Fig 1C for schematic) and correspond to tyrosine residues Y481 and Y504. Interestingly, recent analysis of human non—small cell lung cancer cell line also identified Y481 as undergoing phosphorylation (PhosphoSitePlus; http://www.phosphosite.org), further supporting our analysis.

Y504 and neighboring amino acid residues in the identified peptide are well conserved among different species (79.0% identical amino acids) (S1E Fig), within the Eph family of proteins (55.4% identical amino acids) (Fig 1D), and in other FN3-containing molecules (Figs 1E and S1F). In contrast, Y481 and neighboring residues in the identified peptide are less well conserved amongst different species (63.6% identical amino acids) (S1E Fig), within the Eph family (19.6% identical amino acids), and in other FN3-containing proteins (Figs 1D and 1E and S1F). Because Y481 is found only in EphB2 and was not conserved among other known NMDAR-interacting Eph family members (Fig 1D), we focused our study on Y504.

Our analysis of the F-strand region of cFN3 domains indicates that Y504 is well conserved in proteins that contain homologous domains [11, 26]. Therefore, we next examined mass spectrometry (MS) databases and asked whether FN3-containing molecules that regulate axon guidance and target recognition might also contain phosphorylated tyrosines in FN3 domains that are similar to the cFN3 domain of EphB2. Interestingly, phosphorylated tyrosines have been identified in a number of these proteins at homologous residues. Proteins with previously identified phosphorylation sites include Sidekick1 and Sidekick2 as well as DSCAM1 (S1F Fig). These findings suggest that phosphorylation at EphB2 Y504 may be conserved among various species, Eph family proteins, and other synaptic FN3-containing molecules.

To begin to determine whether Y504 might be phosphorylated, we generated a polyclonal phospho-specific antibody to tyrosine 504 of EphB2. Recognition by the phospho-Y504 antibody (p*Y504) was blocked by preabsorption with the immunogenic phospho-Y504 peptide (S1G Fig). In addition, the p*Y504 antibody recognized full-length EphB2 WT and full-length EphB2 Y481F but not nonphosphorylatable EphB2 Y504F, kinase-dead, or intracellular region—truncated EphB2 mutants (Fig 1F and S1H Fig). These results suggest that the antibody selectively recognizes phosphorylated Y504 and that the kinase domain may play a role in the phosphorylation of EphB2 Y504.

To test whether Y504 phosphorylation of EphB2 is enriched in the cortex and spinal cord, we purified synaptosomes from these regions of WT mice. To validate our synaptosome purification, blots were probed for EphB2, PSD-95, and GluN1 [27]. The EphB p*Y504 signal was enriched in the synaptosome fraction and migrated at the same molecular weight as EphB2. To confirm that the signal detected was due to the presence of phosphate groups, the same blots were incubated with calf intestinal alkaline phosphatase (CIP) (Fig 1G). Incubation with CIP completely removed the signaling from the p*Y504 antibody but had no effect on the signal from the EphB2 antibody. Thus, EphB2 is likely to be phosphorylated on Y504 at synapses in both the brain and spinal cord (S1I Fig).

Finally, to begin to address whether phosphorylation of Y504 on EphB2 might change as synapses mature, we asked whether the phosphorylation of Y504 was developmentally regulated. Synaptosomes were purified from P9, P15, and P21 WT mouse brains and probed for Y504 phosphorylation. We found that the levels of p*Y504 were elevated at P9 and P15 and declined with age. Interestingly, the pattern of p*Y504 parallels decreases in the proportion of NMDARs containing the developmentally regulated NMDAR subunit GluN2B (Fig 1H) [28, 29]. Together these findings indicate that EphB2 is phosphorylated on a highly conserved residue in vitro and in the brain and spinal cord at synaptic sites and this phosphorylation is down-regulated as synapses begin to mature.

### Ligand-inducible phosphorylation of EphB2 occurs on the cell surface

**Fig 2:**
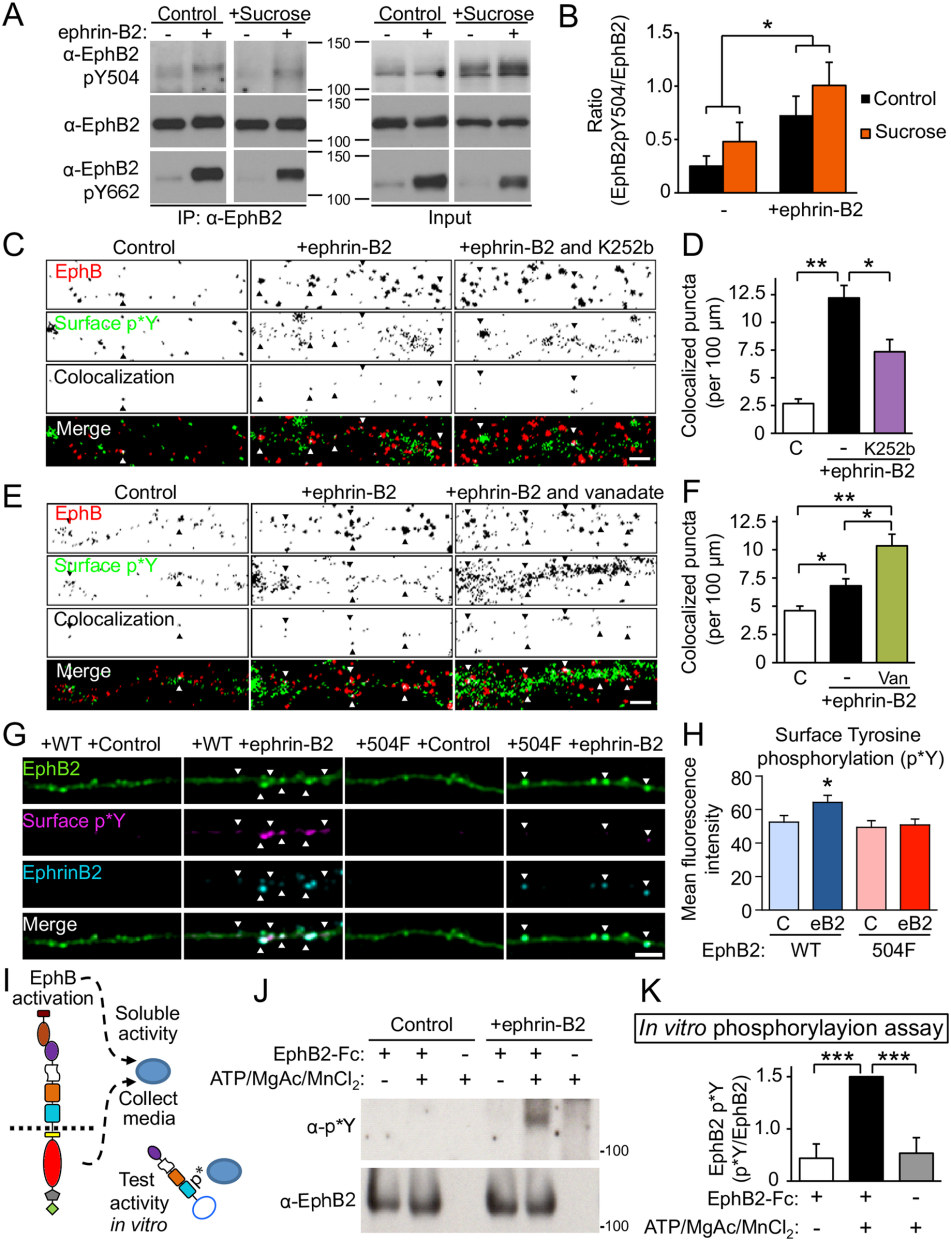
Phosphorylation of Y504 occurs on the surface of neurons. (A) Untransfected cultured rat cortical neurons treated with either ephrin-B2 (+) or control reagents (-) for 45–60 minutes and with either control (C) or sucrose (450 mM, for 15 minutes). In the left panels, EphB2 was immunoprecipitated and blots were probed (listed top to bottom) for EphB2 p*Y504, EphB2, and EphB2 p*Y662. Right panels show neuronal lysates probed in the same manner from the same experiment. (B) Quantification of the ratio of EphB2 Y504 to total EphB2 (control [*n* = 7] and sucrose [*n* = 5]; * *p <* 0.01, 2-factor factorial ANOVA). (C) Images of dendrites stained in live neurons for phosphotyrosine and ephrin-B binding sites (EphBs). Day in vitro (DIV) 7–10 rat cortical neurons were treated with control reagents, activated ephrin-B2 alone, or with 10 μM K252b for 45 minutes and then α-phosphotyrosine antibodies (PY99, p*Y) were added to cells at room temperature for 10 minutes. Neurons were fixed and processed for immunostaining. High-contrast images are shown. Top panels were stained for EphBs, middle panels were live-cell stained for α-p*Y and colocalization, and bottom panels show merged images. In merged panels, red staining indicates surface EphBs, green indicates p*Y, and white indicates colocalization. Arrows indicate examples of colocalized EphB and surface p*Y staining. Scale bar = 2 μm. (D) Quantification of the effects of ephrin-B2 treatment and K252b on surface p*Y staining of EphBs. *n* = 32, 34, and 34 fields from 3 independent experiments for control (C), ephrin B2-Fc treatment, and K252b plus ephrin-B2-Fc treatment, respectively (*p < 0.01, **p < 0.0001, ANOVA followed by Dunn’s multiple comparison test). (E) Images of live-cell stained dendrites as in (C) but treated with ephrin-B2 and vandadate (1 mM for 10 minutes). Scale bar = 2 μm. (F) Quantification of the effects of ephrin-B2 treatment and vanadate treatment on surface p*Y staining of EphB (*p < 0.01, ANOVA followed by Dunn’s multiple comparison test, *n* = 44 fields from 4 independent experiments). (G) DIV 7–10 rat cortical neurons transfected at DIV 3 with td-Tomato and enhanced yellow fluorescent protein (EYFP)-tagged EphB2 wild type (WT), Y504E, or Y504F were treated with either control reagents or activated ephrin-B2 for 45 minutes and then α-phosphotyrosine antibodies (PY99, p*Y) were added to cells at 37°C for 10 minutes. Neurons were fixed and processed for immunostaining. High-contrast images are shown. Top panels were stained for EYFP-tagged EphB2, upper middle panels were live-cell stained for α-p*Y, and lower middle panels were stained for Fc (control Fc or ephrin-B2). Bottom panels show merged images. Arrows indicate examples of triple colocalized puncta. Scale bar = 3 μm. (H) Quantification of the mean fluorescence intensity of surface phosphotyrosine for each condition (*n* = 20 fields from 3 independent experiments, *p < 0.05, ANOVA followed by Dunn’s multiple comparison test). (I) Experimental approach for EphB2 extracellular phosphorylation activity assay in neurons is illustrated. (J) Tyrosine kinase activity in ephrin-B2–treated conditioned artificial cerebrospinal fluid (ACSF) in cultured rat cortical neurons. Medium of cultured neurons was replaced with ACSF, and neurons were treated with ephrin-B2 or control reagents for 45–60 minutes. The extracellular domain of EphB2 fused to the Fc fragment of immunoglobulin G or control reagent was added to filtered ACSF. To activate the activity released from cultured neurons into ATP, MgAc and MnCl_2_ were added. EphB2-Fc was precipitated with Protein G agarose and probed for α-phosphotyrosine (PY99). ACSF was heated to 37°C for 1 hour. Blots were probed as shown. (K) Quantification of the effect of ephrin-B2 treatment in cultured neurons and addition of ATP, MgAc, and MnCl_2_ to isolated conditioned ACSF on the tyrosine phosphorylation of EphB2-Fc (*p <* 0.005, ANOVA followed by Fisher’s exact test, *n* = 4).

To begin to examine the mechanism of EphB2 phosphorylation, we next asked whether EphBs might be phosphorylated on the cell surface after ephrin-B2 stimulation. Cultured cortical neurons were treated with activated ephrin-B2 for 45–60 minutes and then lysates were probed with the p*Y504 antibody. Consistent with the ephrin-B2-dependent activation of EphB2, ephrin-B2 treatment led to a significant increase in Y504 phosphorylation (Fig 2A and 2B). Blockade of EphB2 kinase activity after cell lysis had no effect on ephrin-B2–dependent phosphorylation of Y504 (S2A and S2B Fig), suggesting that phosphorylation of EphB2 at Y504 occurred prior to cell lysis.

EphB2 can be internalized by clathrin-mediated endocytosis after ephrin-B stimulation [30]. To block internalization of EphB2, we blocked clathrin-mediated endocytosis with 450 mM hypertonic sucrose and asked whether ephrin-B2 treatment induced phosphorylation of EphB2 at Y504. Inhibition of endocytosis blocked ephrin-B2–dependent internalization of EphB2 (S2C and S2D Fig) but had no effect on ephrin-B2–induced extracellular tyrosine phosphorylation (p*Y504) of EphB2 (Fig 2A and 2B). These findings suggest that EphB2 undergoes ligand-dependent phosphorylation of Y504 on the cell surface of neurons.

To further test whether the extracellular domain of EphB2 was phosphorylated on the surface of neurons, we conducted live-cell cell surface staining experiments. Because our Y504 phospho-specific antibody is not suitable for immunostaining, these experiments were conducted with the broad-spectrum and well-characterized phosphotyrosine antibody PY99. To induce Y504 phosphorylation, day in vitro (DIV) 7–16 cultured neurons were stimulated with activated soluble ephrin-B2 for 45 minutes, and live-cell staining was conducted with α-phosphotyrosine (PY99) or control antibodies recognizing intracellular proteins. As expected, after treatment with ephrin-B2 or control reagents, live-cell staining with intracellular α-actin antibodies only showed weak background staining (S2E Fig). In contrast, ephrin-B treatment of cultured neurons resulted in a marked increase in tyrosine phosphorylation staining on the cell surface at endogenous sites of ephrin-B binding compared to the control (Fig 2C–2F; *p <* 0.01, ANOVA followed by Dunn's multiple comparison test).

We next asked whether any phosphotyrosine staining might be due to phosphorylation of Y504. To test this, cultured neurons were transfected at DIV 3 with either EphB2 WT or EphB2 Y504F. After ephrin-B2 treatment, surface staining with PY99 resulted in a significant increase in phosphotyrosine staining in the EphB2 WT-transfected neurons but not Y504F-transfected neurons (Fig 2G and 2H; *p <* 0.05, ANOVA followed by Dunn's multiple comparison test). These findings suggest that the majority of the surface phosphotyrosine signal induced by ephrin-B2 treatment can be attributed to phosphorylation at Y504.

To begin to determine whether EphB2 might be phosphorylated by a kinase outside the cell, we blocked kinase activity selectively in the extracellular space by pretreating neurons with a membrane-impermeable ectokinase inhibitor, K252b [31]. In control in vitro experiments, K252b did not block EphB2 kinase activity (S2A Fig). Following 1 hour of K252b treatment of cultured neurons, ephrin-B2–induced surface tyrosine phosphorylation was significantly reduced (Fig 2C and 2D; *p <* 0.01, ANOVA followed by Dunn's multiple comparison test). Moreover, preincubation with the phosphatase inhibitor vanadate potentiated the ephrin-B—induced increase in surface PY99 staining of ephrin-B2 binding sites (Fig 2E and 2F; *p <* 0.01, ANOVA followed by Dunn’s multiple comparison test). These findings suggest that ephrin-B2 stimulation of EphBs in neurons results in the tyrosine phosphorylation of the EphB ectodomain in the extracellular space.

Both soluble and membrane-attached ectokinases have been identified in several cell types, including neurons [32]. We next sought to determine whether ephrin-B stimulation of neurons might induce a soluble activity sufficient to phosphorylate the extracellular domain of EphB2. To address this, the medium of cultured neurons was replaced with artificial cerebrospinal fluid (ACSF), a solution that contains only salts and glucose. Activated ephrin-B2 or control reagents were added to the ACSF, and the ACSF was collected after 45–60 minutes. The conditioned ASCF was then tested for an activity that could phosphorylate the ectodomain of EphB2 (Fig 2I). Treated media alone failed to phosphorylate the extracellular region of EphB2 (Fig 2J). Remarkably, the addition of 100 μM ATP, 10 mM magnesium acetate, and 10 mM manganese chloride to the ephrin-B2 treated ASCF resulted in robust phosphorylation of the EphB2 ectodomain (Fig 2J and 2K; *p <* 0.005, ANOVA followed by Fisher’s exact test). This activity was absent in control ACSF with ATP added or ephrin-B2–treated media without ATP and blocked by heating the ephrin-B2–treated ACSF to 73°C (Fig 2J and S2F and S2G Fig). Taken together with the results from the live-cell staining experiments, these findings indicate that EphB2 Y504 is phosphorylated on the cell surface, likely by an ephrin-B2–induced soluble activity that requires ATP.

### Extracellular phosphorylation of EphB2 is required for interaction with the NMDAR

EphBs are required for normal levels of synaptic NMDARs [14]. EphB2 binds to the NMDAR in both the cortex and the spinal cord [23] via an extracellular domain—dependent interaction that requires ephrin-B [11]. Therefore, we asked whether phosphorylation of EphB2 Y504 might be required for the EphB—NMDAR interaction in cortical and spinal cord neurons. In these experiments, dissociated cortical or spinal cord neurons were treated with soluble activated ephrin-B2 to activate the endogenous EphB—NMDAR interaction. We then conducted experiments to determine whether endogenous EphB2 Y504 was phosphorylated by ephrin-B2 treatment and whether pharmacological blockade of this phosphorylation might block the EphB—NMDAR interaction.

**Fig 3:**
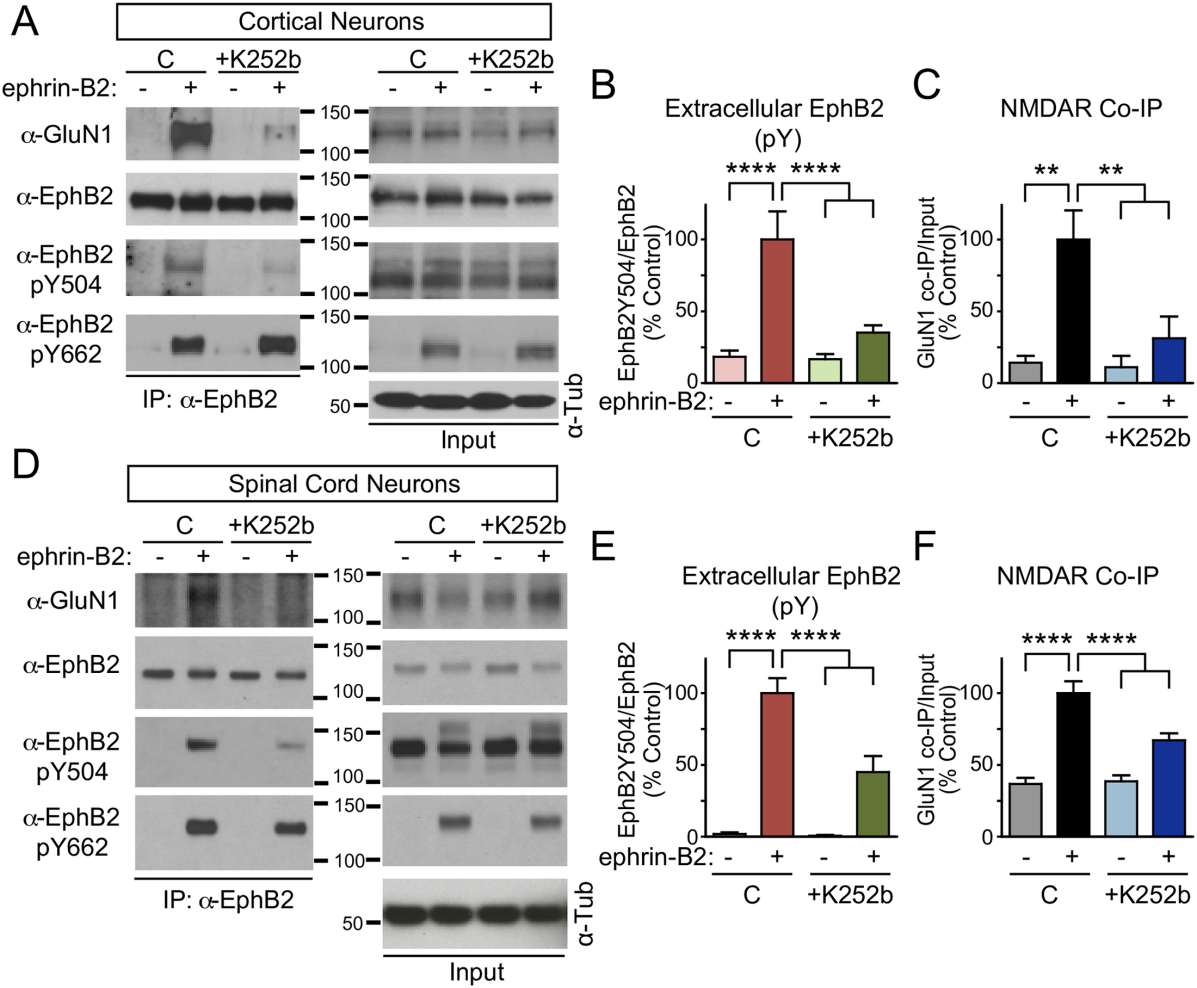
Extracellular phosphorylation is induced by ephrin-B2 and mediates the EphB—N-methyl-D-aspartate receptor (NMDAR) interaction. (A) Untransfected cultured rat cortical neurons (day in vitro [DIV] 6–7) were treated with ephrin-B2 (+) or control reagents (-) for 45–60 minutes and either control (C) or K252b. Endogenous EphB2 was immunoprecipitated using α-EphB2 antibodies, and blots (listed top to bottom) were probed for GluN1, EphB2, EphB2 p*Y504, EphB2 p*Y662, and tubulin. Panels on the left show IP samples and right panels show lysates. (B–C) Quantification of the effects of ephrin-B2 treatment after blockade of extracellular kinase activity with K252b on the phosphorylation of Y504 (B) and the EphB—NMDAR interaction (C) in neurons (**p < 0.01, ****p < 0.001, ANOVA followed by Fisher’s exact test; *n* = 5 experiments for each condition). (D) Untransfected cultured rat spinal cord neurons (DIV 12–14) were treated with ephrin-B2 (+) or control reagents (-) for 45–60 minutes and either control (C) or K252b. Endogenous EphB2 was immunoprecipitated using α-EphB2 antibodies and blots (listed top to bottom) were probed for GluN1, EphB2, EphB2 p*Y504, EphB2 p*Y662, and tubulin. (E–F) Quantification of the effects of ephrin-B2 treatment after blockade of extracellular kinase activity with K252b (****p < 0.001, ANOVA followed by Fisher’s exact test; *n* = 5 experiments for each condition).

To determine whether the EphB2–NMDAR interaction requires Y504 phosphorylation, endogenous extracellular kinase activity was blocked with K252b. Neurons were then treated with activated ephrin-B2 for 45–60 minutes, endogenous EphB2 was immunoprecipitated in a RIPA buffer, and blots were probed for endogenous GluN1 and p*Y504. Ephrin-B2 treatment of DIV 6–7 cultured cortical and spinal cord neurons effectively induced the EphB—NMDAR interaction and phosphorylation of Y504 (Fig 3A–3F). Pretreatment of neurons for 1 hour with K252b caused a significant decrease in phosphorylation of Y504 (*p <* 0.001, ANOVA followed by Fisher’s exact test; Fig 3B and 3E). K252b treatment did not alter the ability of ephrin-B2 treatment to stimulate EphB2 intracellular phosphorylation (Fig 3D and S3A Fig), suggesting that K252b does not alter the ability of ephrin-B to activate the EphB kinase. However, K252b treatment did cause a significant decrease in the ephrin-B—induced EphB—NMDAR interaction (**p < 0.01, ****p < 0.001, ANOVA followed by Fisher’s exact test; Fig 3C and 3F). These effects were particularly robust in cortical neurons but resulted in significant decreases in both cortical and spinal cord neurons. These data indicate that blockade of endogenous p*Y504 blocks the ability of EphB2 to interact with the NMDAR and suggest that the extracellular phosphorylation of EphB2 at Y504 is required for the EphB—NMDAR interaction.

Induction of phosphorylation requires ATP hydrolysis. If phosphorylation of Y504 is necessary for the EphB—NMDAR interaction, we expect that blocking extracellular ATP hydrolysis should block the interaction. Neurons were treated with the nonhydrolyzable ATP analogue ATPγS (1 μM) for 1 hour before ephrin-B treatment, and the effect on the EphB—NMDAR interaction was determined by co-IP. Bath application of ATPγS significantly reduced the ephrin-B—induced EphB—NMDAR interaction without affecting intracellular tyrosine phosphorylation of EphB2 (***p < 0.005, ****p < 0.001, ANOVA followed by Fisher’s exact test; S3B–S3D Fig). These findings suggest that phosphorylation of EphB2 at Y504 is required for the EphB—NMDAR interaction and that ephrin-B—dependent induction of this interaction can be blocked by blocking ATP hydrolysis in the extracellular space.

### Phosphorylation of EphB2 is necessary and sufficient for the EphB—NMDAR interaction

**Fig 4:**
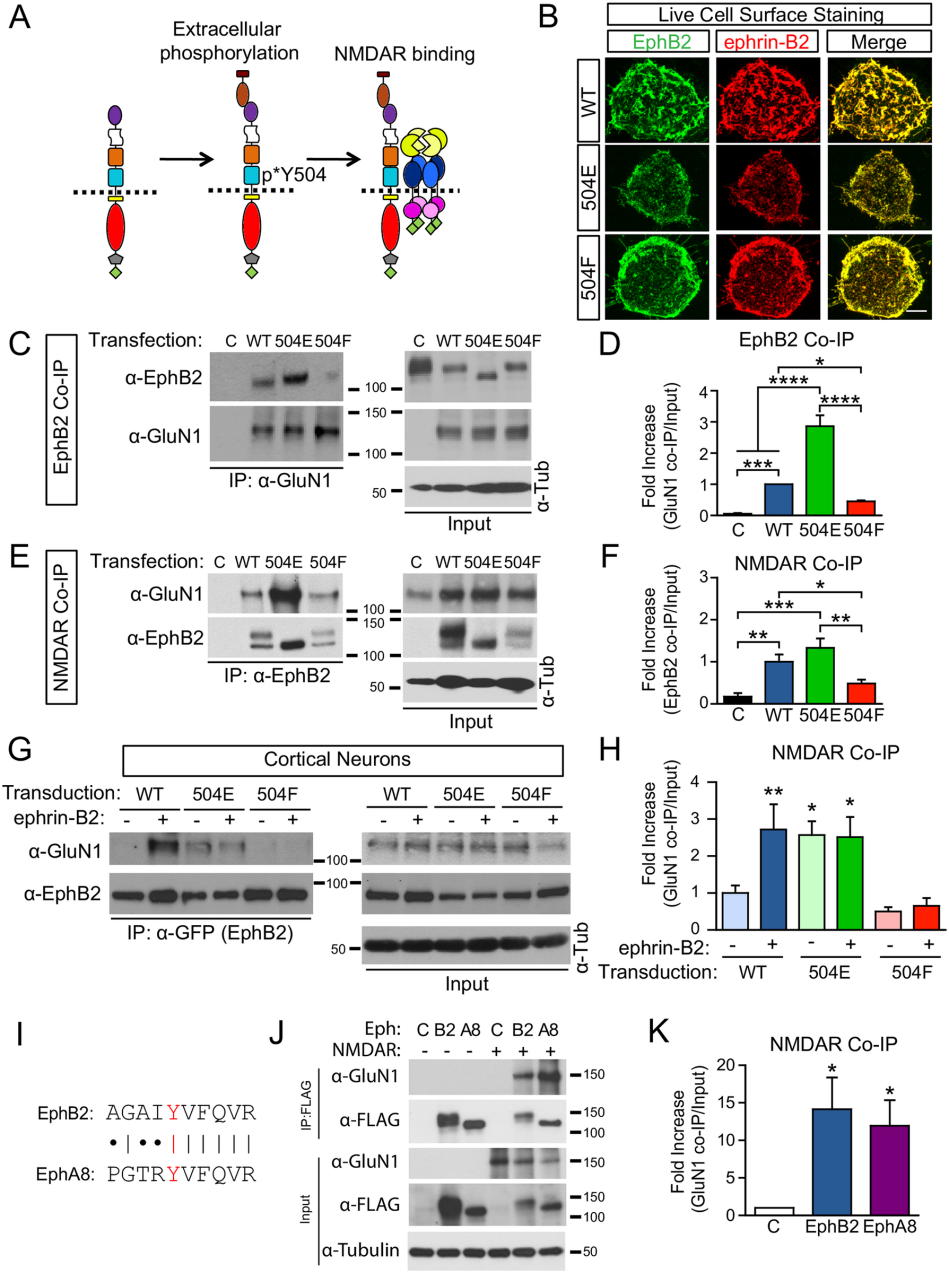
Phosphorylation of extracellular tyrosine residue 504 of EphB2 is required for interaction with the N-methyl-D-aspartate receptor (NMDAR). (A) Model of how extracellular phosphorylation at Y504 modulates the EphB—NMDAR interaction. (B) Live-cell immunostaining of HEK293T for FLAG(-EphB2) and ephrin-B2-Fc—binding sites. HEK293T cells transfected with FLAG-EphB2 wild type (WT), Y504E, or Y504F mutants were incubated with ephrin-B2-Fc for 45 minutes and then α-FLAG antibody was added to cells at 37°C for 10 minutes. After washing, cells were fixed and processed for immunostaining. Left panels were live-cell stained for FLAG-tagged EphB2, middle panels were stained for ephrin-B2-Fc, and right panels show merged images. Quantified in S4A Fig. For surface staining controls, see S4B–S4D Fig. Scale bar = 5μm. (C) Coimmunoprecipitation (co-IP) of FLAG-EphB2 and GluN1 with α-GluN1 antibodies from HEK293T. Left control (C) lane has only FLAG-EphB2 WT. Other lanes are transfected with GluN1 and GluN2B and indicated EphB2 constructs. The right WT lane is transfected with FLAG-EphB2 WT, the Y504E lane is transfected with FLAG-EphB2 Y504E, and the Y504F lane is transfected with FLAG-EphB2 Y504F. Antibodies used for detection are shown at the left. IP samples are shown in blots on the left; cell lysates are shown on the right. Tubulin is used as loading control. (D) Quantification of ratio of coimmunoprecipitated EphB2 to total EphB2 in input. EphB2 Y504E coimmunoprecipitated with GluN1 significantly more than EphB2 WT. In addition, GluN1 co-IP of EphB2 Y504E was significantly higher than EphB2 WT (*p < 0.05, ***p < 0.005, ****p < 0.001, ANOVA followed by Fisher’s exact test, *n* = 4). (E) Co-IP of GluN1 and FLAG-tagged EphB2 with α-EphB2 antibodies from HEK293T. All lanes are transfected with HA-GluN1 and GluN2B. The control lane had only HA-GluN1 and GluN2B alone, the WT lane was transfected with FLAG-EphB2, the E lane was transfected with FLAG-EphB2 Y504E, and the F lane was transfected with FLAG-EphB2 Y504F. Top blots were probed for HA (GluN1), and the bottom blots were probed for FLAG (EphB2). Left panels are IP samples and right panels are lysates. Tubulin was used as a loading control. (F) Quantification of ratio of coimmunoprecipitated GluN1 to total GluN1 in the input. IP of FLAG-tagged EphB2 mutants revealed a significant increase of GluN1 co-IP in lysates from cells expressing EphB2 Y504E, while GluN1 co-IP was significantly reduced in lysates from cells expressing the Y504F EphB2 mutants compared to GluN1 pull-down in EphB2 WT-expressing cells (*p < 0.05, **p < 0.01, ***p < 0.005, ANOVA followed by Fisher’s exact test, *n* = 5). (G) Cultured rat cortical neurons (day in vitro [DIV] 9) infected with lentivirus harboring either enhanced yellow fluorescent protein (EYFP)-tagged EphB2 WT or Y504 mutants at DIV 2 were stimulated with ephrin-B2-Fc or control. EphB2 was immunoprecipitated with α-GFP antibodies and blots were probed for GluN1 (top left). WT indicates neurons transduced with EphB2-EYFP, Y504E indicates neurons transduced with EphB2 Y504F EYFP, and Y504F indicates neurons transduced with EphB2 Y504F EYFP. Minus sign (-) indicates control treatment, plus sign (+) indicates ephrin-B2 treatment. Top blots were probed for GluN1 (α-GluN1), and bottom blots were probed for transduced EphB2 (α-GFP). Left panels are IP samples and right panels are lysates. Tubulin was used as a loading control. (H) Quantification of ratio of coimmunoprecipitated GluN1 to total GluN1 in input. In control neurons infected with EphB2-EYFP, GluN1 coimmunoprecipitates robustly with EphB2 pull-down after ephrin-B2 treatment (top-left blot in E). In neurons expressing the Y504E mutant, GluN1 coimmunoprecipitates under control conditions without ephrin-B treatment (*p* = 0.0106, ANOVA followed by Fisher’s exact test, *n* = 6), and ephrin-B treatment did not potentiate GluN1 co-IP (*p* = 0.919, *n* = 6). Y504F mutants demonstrate little pull-down with GluN1 in absence of ephrin-B treatment (*n* = 6), and ephrin-B stimulation did not potentiate the EphB—NMDAR interaction in Y504 mutants (*p* = 0.549, ANOVA followed by Fisher’s exact test; *n* = 6). (I) Alignment of homologous regions of fibronectin type III (FN3) domains of EphB2 and EphA8. (J) IP of FLAG-EphB2 and FLAG-EphA8 from HEK293T cells cotransfected with HA-GluN1 and GluN2B. The first 3 lanes are transfected without NMDAR. The last 3 lanes are transfected with HA-GluN1 and GluN2B. The Control lane has only HA-GluN1 and GluN2B alone, B2 is transfected with FLAG-EphB2, and the A8 lane is transfected with FLAG-EphA8. Top blots were probed for α-GluN1, and bottom blots were probed for FLAG (EphB2). Left panels are IP samples and right panels are lysates. Tubulin was used as a loading control. (K) Quantification of ratio of coimmunoprecipitated GluN1 to FLAG IP. Immunoprecipitation from HEK293T cells transfected with FLAG-EphB2 or FLAG-EphA8 and GluN1 and GluN2B revealed that both EphA8 and EphB2 can effectively co-IP the GluN1 subunit of the NMDAR (*p < 0.05 versus control, ANOVA followed by Fisher’s exact test, *n* = 4).

Phosphorylation of Y504 appears to be required for the EphB—NMDAR interaction in neurons. Therefore, we tested whether the phosphorylation of Y504 within the cFN3 of EphB2 might be necessary and sufficient for the EphB—NMDAR interaction (Fig 4A). To test this possibility, we generated an EphB2 phosphomimetic mutant (Y504E) and a nonphosphorylatable mutant (Y504F) and then examined the interaction between EphB2 mutants and GluN2B-containing NMDARs in HEK293T cells by co-IP. The ability of surface EphB2 to bind to ephrin-B2 was not altered by mutation of Y504 (Fig 4B and S4A–S4D Fig, *p* > 0.05, ANOVA), suggesting that the structure of the extracellular domain remains intact after mutation of this residue. In HEK293T cells, mutations to Y504 modulated the rate of removal of EphB2 from the cell surface; however, these effects were not seen in neurons (S5A–S5G Fig).

Mutation of the second phosphotyrosine identified by MS (Y481) had no effect on the ability of EphB2 to interact with the NMDAR, suggesting that this amino acid is not required for the EphB—NMDAR interaction (S4E and S4F Fig). In contrast, mutations of EphB2 at Y504 resulted in profound changes. Expression of the phosphomimetic EphB2 Y504E mutant resulted in a significant increase in EphB2 co-IP when pulled down with GluN1 antibody, while expression of the nonphosphorylatable EphB2 Y504F mutant caused a significant decrease in co-IP compared to WT (Fig 4C and 4D; *p* > 0.001, ANOVA followed by Fisher’s exact test). Similar effects were observed when antibodies against FLAG (FLAG-EphB2 or FLAG-EphB2 mutants Y504E or Y504F) were used to immunoprecipitate GluN1 (Fig 4E and 4F). These results indicate that the charge of this residue within the extracellular domain of EphB2 is necessary and sufficient for the EphB—NMDAR interaction in HEK293T cells.

To test the effect of EphB2 Y504 mutants in neurons, DIV 2 cultured cortical neurons were transduced with lentiviruses expressing enhanced yellow fluorescent protein (EYFP)-tagged EphB2 WT, EphB2-Y504E, or EphB2-Y504F. Neurons were then challenged with ephrin-B2 treatment at DIV 9 for 45–60 minutes, and the ability of the GluN1 subunit of the NMDAR to co-IP with EphB2 was tested. In the control group, cultured neurons were transduced with EphB2 WT. In these neurons, little EphB—NMDAR interaction was detectable without ephrin-B stimulation, but ephrin-B stimulation induced a significant increase in the co-IP of GluN1 with EYFP-tagged EphB2 WT (Fig 4G and 4H; *p* = 0.0056, ANOVA followed by Fisher’s exact test). In contrast, neurons transduced with Y504E mutant EphB2 showed robust GluN1 pull-down even in the absence of ephrin-B treatment (Fig 4G and 4H; *p* = 0.0106, ANOVA followed by Fisher’s exact test), and ephrin-B treatment of neurons transduced with EYFP-tagged EphB2 Y504E did not further increase the EphB—NMDAR interaction (Fig 4G and 4H; *p* = 0.919, ANOVA followed by Fisher’s exact test). These findings suggest that Y504 phosphorylation is sufficient to induce the EphB2–NMDAR interaction in neurons. Neurons transduced with the Y504F mutant form of EphB2 failed to show GluN1 co-IP with EphB2, either with or without ephrin-B treatment (Fig 4G and 4H; *p* = 0.549, ANOVA followed by Fisher’s exact test). These data indicate that phosphorylated EphB2 Y504 is both necessary and sufficient for the EphB—NMDAR interaction in neurons and suggest that extracellular phosphorylation of a tyrosine in the cFN3 domain of EphBs may drive protein—protein interactions.

To test whether phosphorylation of specific tyrosine residues and surrounding amino acids in the FN3 domain might provide a mechanism to mediate protein—protein interactions, we examined whether other Eph proteins with sequences similar to that found in EphB2 might interact with the NMDAR. EphB1–3 all interact with the NMDAR and have a similar set of amino acids near Y504 (XpYVXQVR), while the sequences of EphA3 and EphA4, which do not interact with the NMDAR, differ [11]. Interestingly, the sequence of EphA8, which was not previously known to interact with the NMDAR, is identical to EphB2 in this region (Fig 4I). To test whether EphA8 can associate with the NMDAR, we generated a FLAG-tagged EphA8 expression construct and coexpressed it along with GluN1 and GluN2B in HEK293T cells. We found that FLAG-EphA8 efficiently coimmunoprecipitates with the NMDAR from HEK293T cell lysates (Fig 4J and 4K). EphA8 is only 49.0% homologous to EphB2 outside of this region, while EphA4, which does not interact with the NMDAR, is 58.5% homologous; thus, these findings suggest that tyrosines with surrounding amino acids similar to EphB2 might be used to predict the ability of proteins to interact with the NMDAR.

### EphB2 Y504 controls synaptic NMDAR function

**Fig 5:**
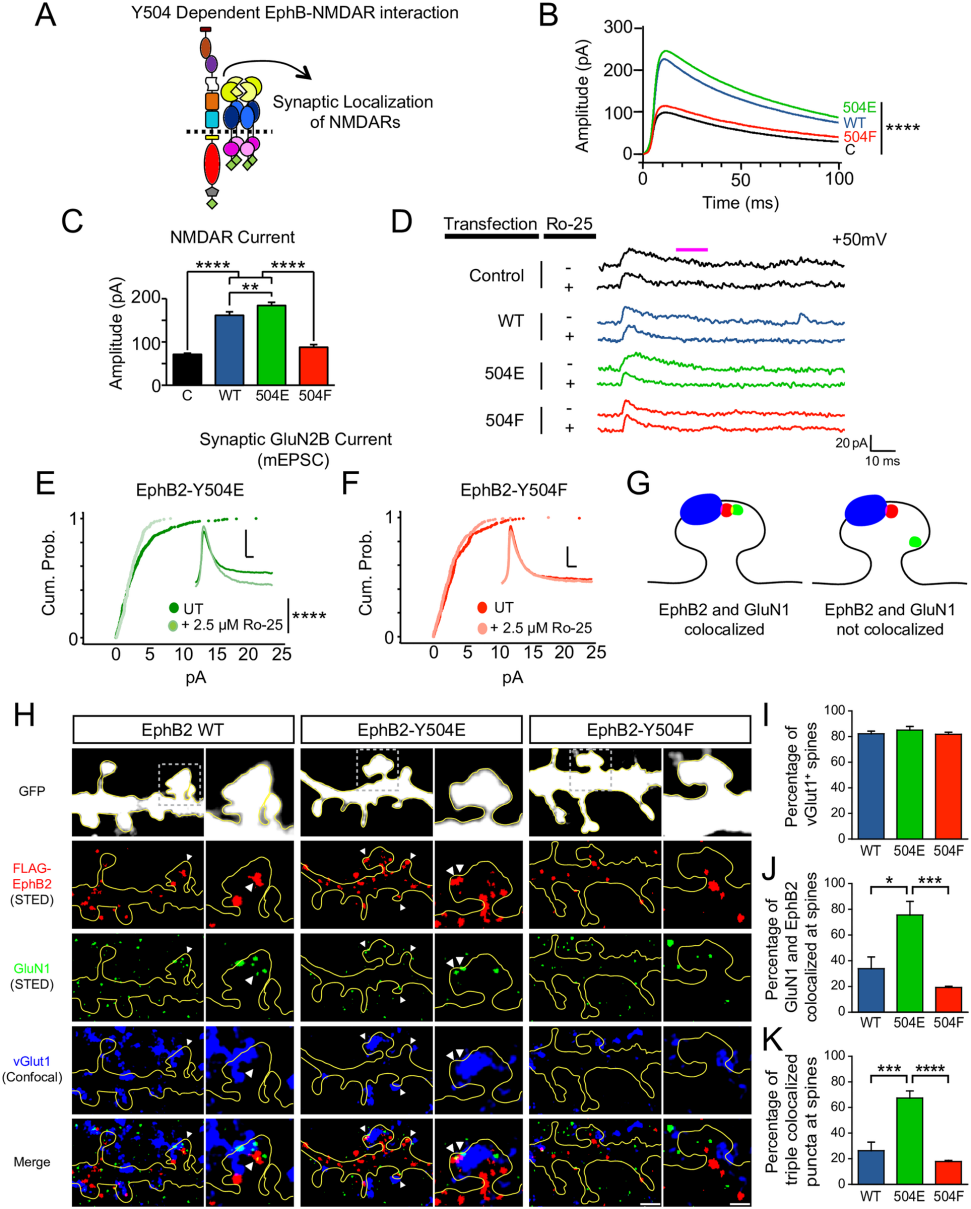
Synaptic currents and accumulation of N-methyl-D-aspartate receptors (NMDARs) at synaptic sites are controlled by phosphorylation of EphB2 Y504. (A) Experimental approach for the figure is illustrated. (B) Mean traces of whole-cell patch-clamp recording at 50 mV show the evoked and miniature excitatory postsynaptic currents (EPSCs) of primary rat cortical day in vitro (DIV) 21–23 neurons expressing enhanced green fluorescent protein (EGFP) without or with EphB2 wild type (WT) and Y504 mutants induced by light stimulation of neurons that express optogenetic light-sensitive channels (channelrhodopsin-2). (C) Effects of overexpression of EphB2 WT and Y504 mutants on the mean amplitude of evoked, spontaneous, and miniature EPSCs (30 milliseconds after the evoked EPSC peak) in mature cultured neurons. Overexpression of EphB2 WT or Y504E significantly increased amplitude of the NMDAR-dependent component of evoked EPSCs compared to the control or the Y504F mutant (****p < 0001, ANOVA followed by Fisher’s exact test, *n* = 375, 332, 581, and 200 events for control, EphB2 WT, Y504E, and Y504F, respectively). In addition, the amplitude of Y504E-overexpressing neurons was significantly higher than that of WT-overexpressing neurons (**p < 0.02, ANOVA followed by Fisher’s exact test). (D) Representative sample traces of whole-cell patch-clamp recording at 50 mV show that NMDAR-dependent currents in control neurons and neurons expressing EphB2 WT and Y504E, but not Y504F, are greatly reduced by GluN2B-specific antagonist Ro 25–6981 (Ro25). (E) Cumulative probability histogram of miniature EPSC (mESPC) amplitude for Y504E before and after application of Ro25 (2.5 μM). Inset: mean traces of mEPSCs after treatment with Ro25 (*p <* 0.001, Kolmogorov—Smirnov [K–S] test, *n* = 720 for Y504E and *n* = 427 for Y504E + Ro25). Vertical scale bar = 20 picoamperes (pA); horizontal scale bar = 10 milliseconds. (F) Cumulative probability histogram of mEPSC amplitude Y504F as in (E) (*p* = 0.0775, K–S test, *n* = 414 for Y504F and *n* = 404 for Y504F + Ro25). Vertical scale bar = 10 pA; horizontal scale bar = 10 milliseconds. (G) Model depicting 2 scenarios of VGLUT1 (blue), EphB2 (red), and GluN1 (green) localization at dendritic spines. (H) High-contrast images of dendrites of DIV 21 cortical neurons expressing EGFP, EphB2 short hairpin RNA (shRNA), and RNA interference (RNAi)-insensitive FLAG-tagged EphB2 WT, EphB2-Y504E, or EphB2-Y504F. Top panels show confocal EGFP staining (white), second panels show stimulated emission depletion (STED) EphB2 staining (red), third panels show STED GluN1 staining (green), fourth panels show confocal VGLUT1 (presynaptic marker) staining (blue), and bottom panels show merged images. White arrows indicate examples of triple colocalization of EphB2, GluN1, and VGLUT1. Scale bar = 1 μm, 0.5 μm inset. (I) Quantification of the effects of expression of EphB2 Y504 mutants on localization of VGLUT1 to dendritic spines in DIV 21 rat cortical neurons transfected at DIV 14. Graph shows fraction of spines containing VGLUT1 (not significant, ANOVA followed by Fisher’s exact test). (J) Quantification of the effects of expression of EphB2 Y504 mutants on colocalization with GluN1 in dendritic spines in DIV 21 rat cortical neurons transfected at DIV 14. Graph shows percentage of spines containing colocalized EphB2 and GluN1 puncta as defined by Fig 5G (*p < 0.05, ***p < 0.005, ANOVA followed by Fisher’s exact test). (K) Quantification of the effects of expression of EphB2 Y504 mutants on colocalization with GluN1 at synaptic sites in DIV 21 rat cortical neurons transfected at DIV 14. Graph shows percentage of spines containing triple colocalized puncta as defined by Fig 5G (***p < 0.005, ****p < 0.0005, ANOVA followed by Fisher’s exact test).

Early in development, EphBs function to control synapse formation [13], but later in neuronal development, EphBs are key regulators of NMDAR localization and function at synapses [14]. To test whether the extracellular phosphorylation of EphB2 Y504 is required for EphB-dependent NMDAR function and synaptic accumulation, we asked if mutation of EphB2 at Y504 might alter the NMDAR-dependent synaptic currents in cortical neurons (Fig 5A). To avoid effects on synapse development, neurons were transfected at DIV 14 with enhanced green fluorescent protein (EGFP) with or without EYFP-tagged EphB2 WT, Y504E, or Y504F constructs [14]. Cultures were infected at DIV 10 with adeno-associated virus transducing channelrhodopsin-2 to enable optical activation [33] (Penn Vector Core). To evaluate the strength of evoked NMDAR currents, we measured both spontaneous and optogenetically evoked synaptic currents in neurons at DIV 21–23 using whole-cell patch-clamp recording at 50 mV. Expression of EYFP-tagged EphB2 WT and Y504E both caused a significant increase in amplitude of the NMDAR-dependent component of excitatory postsynaptic currents (EPSCs) compared to the control or the Y504F mutant (Fig 5B and 5C; NMDAR component measured 30 milliseconds after the evoked EPSC peak; ****p < 0.001, ANOVA followed by Fisher’s exact test). In addition, the amplitude of Y504E-expressing neurons was significantly higher than that of WT-expressing neurons (**p < 0.02, ANOVA followed by Fisher’s exact test).

To determine whether these effects were due to changes to the NMDAR at synaptic sites, spontaneous miniature excitatory postsynaptic currents (mEPSCs) were recorded in the presence of tetrodotoxin and blockers of GABAergic channels. Consistent with previous work [14], no significant changes in mEPSC frequency were observed between conditions (S6A and S6B Fig). As expected, expression of EYFP-tagged EphB2 WT and Y504E both caused a significant increase in amplitude of the NMDAR-dependent component of mEPSC compared to control or Y504F mutants (S6C and S6D Fig). These changes in mEPSC amplitude are attributable specifically to the increase of synaptic NMDARs because treatment with the NMDAR antagonist D-2-amino-5-phosphonovalerate (D-APV) (50 μM) blocked the effects of EphB overexpression and reduced mEPSC amplitude to a similar size in all transfection conditions (S6E and S6F Fig; NMDAR component measured 15–25 milliseconds after the mEPSC peak; *p <* 0.01; ANOVA followed by Fisher’s exact test). Cumulative probability histograms of events from neurons transfected with EphB2 Y504E or EphB2 Y504F indicate that the NMDAR-dependent component of mEPSC amplitude is greater in EphB2 Y504E than in EphB2 Y504F—expressing neurons (S6F Fig, *p <* 0.0001, Kolmogorov—Smirnov [K–S] test).

APV blocks all NMDARs, but previous work suggests that EphBs may preferentially act on GluN2B-containing NMDARs in younger neurons [14]. Therefore, we asked whether mutations to EphB2 at Y504 that enhance (Y504E) or prevent (Y504F) the EphB—NMDAR interaction might drive or exclude GluN2B-containing NMDAR from synapses. We selectively blocked GluN2B-containing NMDAR with the GluN2B-specific antagonist Ro 25–6981 (Ro-25) and determined the effect on the NMDAR component of mEPSCs in cultured cortical neurons. Whole-cell patch-clamp recordings of mEPSCs revealed that NMDAR-dependent currents in neurons transfected with EphB2 Y504E decreased significantly with selective inhibition of GluN2B-containing NMDARs (Fig 5D and 5E, *p <* 0.001, K–S test). However, Ro-25 treatment had no significant effect on mEPSC amplitude in Y504F-overexspressing neurons (Fig 5D and 5F). These data suggest that EphB2 preferentially recruits GluN2B-containing NMDARs to synaptic sites.

We next asked whether the accumulation of NMDARs at synaptic sites is altered by expression of EphB2 Y504 mutants in mature neurons. To test this, we conducted 2-color super-resolution imaging using stimulated emission depletion (STED) microscopy of FLAG-EphB2 or FLAG-EphB2 mutants (Y504E and Y504F) and endogenous GluN1 combined with 2-color confocal imaging of vGlut1 and cell-filling EGFP expressed in DIV 21–23 cortical neurons [34]. EphBs are required for normal numbers of dendritic spine synapses and the proper number of NMDARs at synaptic sites [13]. Therefore, we focused on analyzing spine synapses. Synapses were defined by the presence of 1 or more vGlut1 puncta that contacted a spine (Fig 5G). Consistent with our previous findings and physiological data, the percentage of vGlut1-positive spines did not differ between the transfection conditions, with approximately 80% of imaged spines containing vGlut1 puncta (Fig 5I) [14].

Using 2-color STED super-resolution imaging, we determined the proportion of vGlut1-positive spines containing FLAG-EphB2 and GluN1 puncta. Approximately 30% of spines contained FLAG-EphB2 WT puncta that colocalized with 1 or more puncta of GluN1 (Fig 5G, 5H and 5J). We next examined the impact of the expression of FLAG-EphB2 Y504E. FLAG-EphB2 Y504E interacts with the NMDAR independent of ephrin-B ligand activation; therefore, we expect that expression of FLAG-EphB2 Y504E would increase the number of synapses where EphB2 and the GluN1 subunit of NMDAR colocalize. Indeed, STED analysis revealed that both the fraction of spines containing colocalized FLAG and GluN1 puncta and the fraction of spines with GluN1 colocalization with EphB2 at synaptic sites were significantly increased in Y504E-expressing neurons compared with EphB2 WT- or Y504F-expressing neurons (*p < 0.05, **p < 0.01, ANOVA followed by Fisher’s exact test; Fig 5G, 5H, 5J and 5K). Interestingly, in these experiments, expression of EphB2 Y504F did not appear to act in a dominant negative manner. Although we knocked down endogenous EphB2, the lack of an effect of EphB2 Y504F might be due to the presence of other EphB proteins or might reflect the presence NMDAR-interacting proteins such as PSD-95. Regardless, these findings indicate that phosphorylation of Y504 enables EphB2 to recruit or retain NMDARs to spine synapses.

### Y504 regulates NMDAR localization in spinal cord

**Fig 6:**
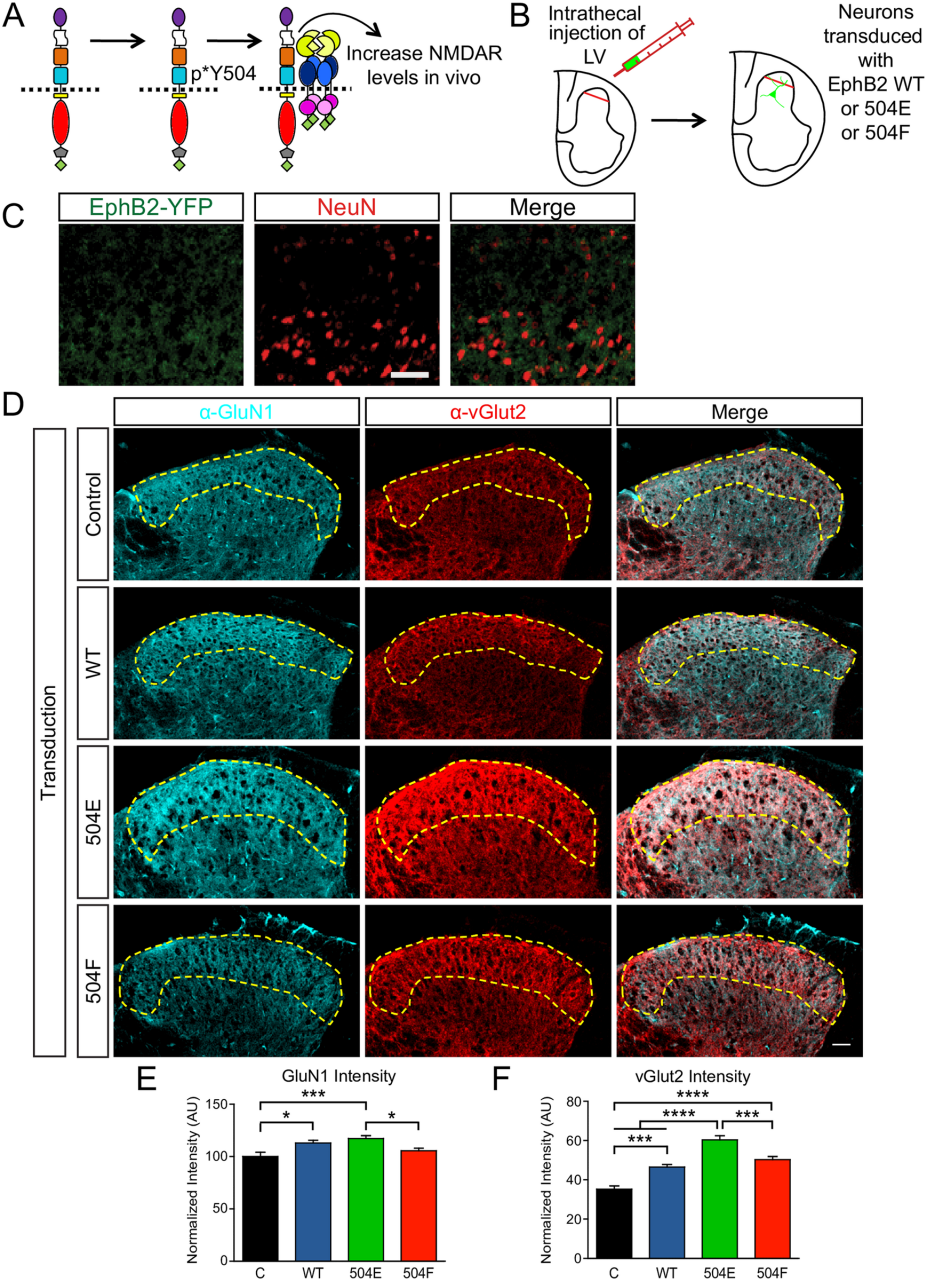
Expression of EphB2 recruits the N-methyl-D-aspartate receptor (NMDAR) to synaptic regions of the dorsal horn. (A) Model for effects of EphB2 extracellular phosphorylation on the EphB—NMDAR interaction. (B) Experimental approach using intrathecal injection of lentivirus (LV) to avoid effects outside of the spinal cord. Examination of injection site revised transduced neurons within the dorsal horn. (C) Neuronal viral transduction was confirmed with NeuN labeling (marker for neuronal nucleus) surrounded by EphB2- enhanced yellow fluorescent protein (EYFP) wild type (WT). The left panel was stained for EphB2-EYFP with α-GFP. The middle panel was stained for NeuN. The right panel shows the merged image. Control enhanced green fluorescent protein (EGFP), EphB2-EYFP Y504E, and EphB2-EYFP Y504F also show the distribution surrounding NeuN. Scale bar = 50 μm. (D) Distribution of GluN1 and vGlut2 in the dorsal horn of the adult mouse spinal cord, expressing control EGFP, EphB2-EYFP WT, or Y504E or Y504F mutants. The left panel shows GluN1 (cyan). The middle panels show vGlut2 (red) to mark superficial layers of dorsal horn. The right panel shows a merged image of GluN1 and vGlut2 staining. The dashed yellow line indicates superficial layers of dorsal horn. Scale bar = 50 μm. (E) Quantification of the effects of expression of EphB2 WT and Y504E and Y504F mutants on GluN1 intensity in superficial layers of the dorsal horn of the adult mouse spinal cord (*p < 0.05, ***p < 0.05, ANOVA followed by Tukey’s range test, 17 sections from 3 mice for control, 18 sections from 3 mice for EphB2 WT, 22 sections from 4 mice for Y504E mutant, and 24 sections from 4 mice for Y504F mutant). (F) Quantification of the effects of expression of EphB2 WT and Y504E and Y504F mutants on vGlut2 intensity in superficial layers of the dorsal horn of the adult mouse spinal cord. (***p < 0.0005, ****p < 0.0001, ANOVA followed by Tukey’s range test, 17 sections from 3 mice for control, 18 sections from 3 mice for EphB2 WT, 22 sections from 4 mice for Y504E mutant, and 24 sections from 4 mice for Y504F mutant). AU, arbitrary unit.

Despite normal levels of NMDARs in EphB triple-knockout mice, in these animals NMDARs are redistributed from synaptic sites to extrasynaptic locations, suggesting that EphBs play an important role in regulating NMDAR localization [14]. To test whether Y504 might regulate the recruitment of NMDARs in vivo, neurons in the dorsal horn of the spinal cord were transduced by intrathecal injection of lentivirus coding for either EYFP-tagged EphB2 WT or the constitutively interacting EYFP-tagged EphB2 Y504E mutant (Fig 6A and 6B). This method results in transduction of approximately 60% of NeuN-positive cells in the region near the injection site [35]. Consistent with our previous findings, injection of the virus resulted in a focal infection of neurons (NeuN-positive cells, Fig 6C) within the dorsal aspect of the spinal cord near the injection site (Fig 6B–6D).

The superficial nociceptive layers of the dorsal horn are vGlut1-negative and vGlut2-positive [36, 37]. To determine whether transduction of EYFP-tagged EphB2 results in changes in NMDAR expression within the nociceptive region of the spinal cord, sections were stained for GluN1 and vGlut2. In mice transduced with EYFP-tagged EphB2 WT or Y504E, the expression of the GluN1 subunit of the NMDAR was significantly up-regulated in the superficial layers of the dorsal horn compared to control and Y504F-injected mice (*p <* 0.05, ANOVA followed by Tukey’s range test; Fig 6D and 6E). In addition to the increase in NMDAR levels, there was also a significant increase in vGlut2 intensity (*p <* 0.0005, ANOVA followed by Tukey’s range test; Fig 6D and 6F). These changes in presynaptic vesicle markers are consistent with the known function of EphB2 in the induction of presynaptic terminal formation. Evidence suggests that different domains might mediate the role of EphB2 in synapse formation and the EphB—NMDAR interaction, with the ephrin-B binding domain of EphB2 being essential for induction of presynaptic differentiation [12, 38]. Consistent with this model, transduction of EphB2 Y504F resulted in a significant increase in the intensity of vGlut2 but not in GluN1 levels (Fig 6E and 6F). Regardless, these findings suggest that Y504 of EphB2 regulates the localization of the NMDAR in the spinal cord.

### Extracellular phosphorylation regulates pathological pain

**Fig 7:**
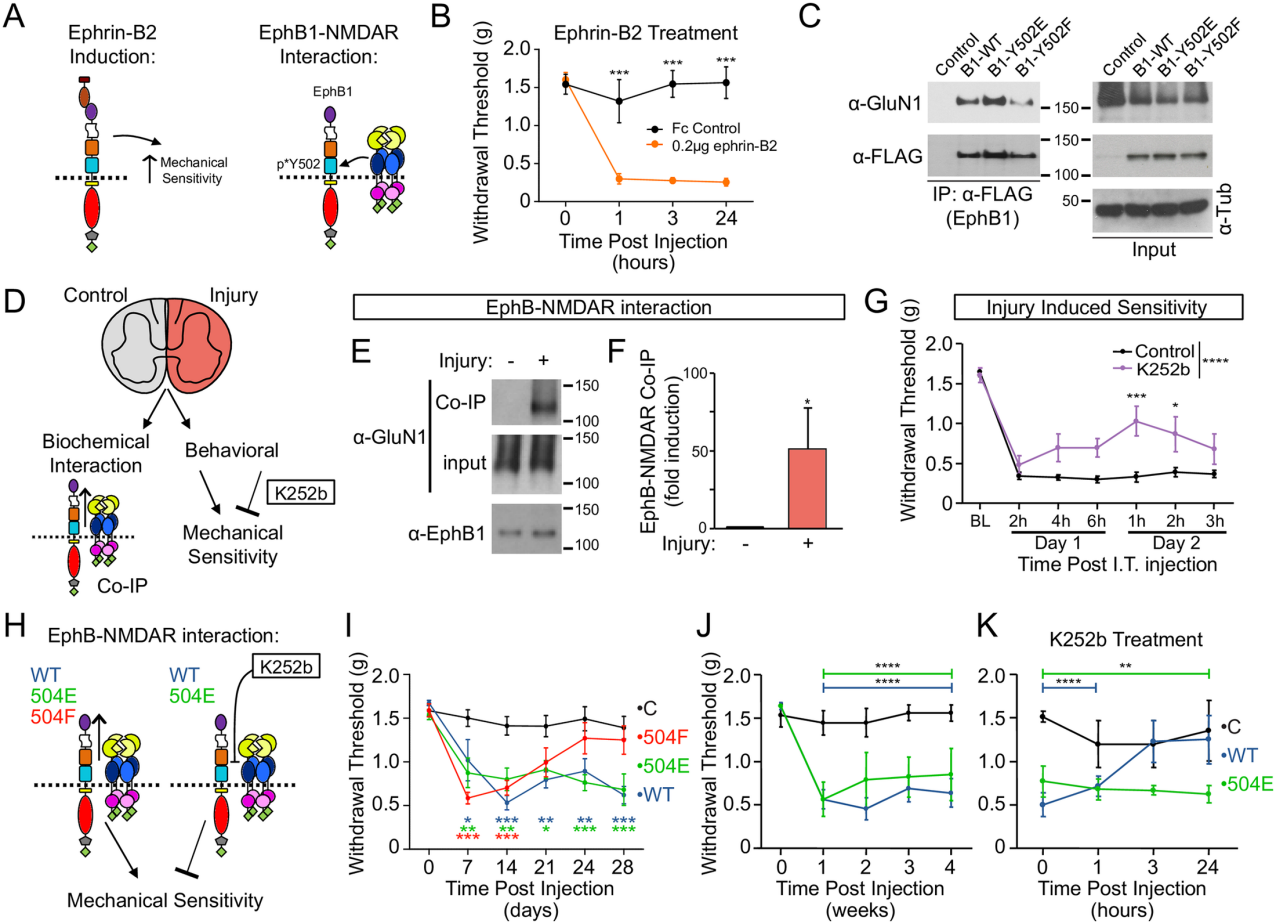
Pain sensitivity is regulated by EphB2 Y504 and extracellular phosphorylation. (A) Experimental approach for panels B–C is illustrated. (B) Quantification of the effects of intrathecal (I.T.) injection of ephrin-B2-Fc (0.2 μg) on mechanical sensitivity in adult mice (***p < 0.001, ANOVA followed by Dunnet’s multiple comparison test, *n* = 6 mice). (C) Co-immunoprecipitation (co-IP) of GluN1 and EphB1 with α-FLAG (EphB1) antibody from HEK293T. All lanes are transfected with HA-GluN1 and GluN2B. The control lane is transfected with enhanced green fluorescent protein (EGFP), the wild type (WT) lane is transfected with EphB1 WT, the E lane is transfected with EphB1 Y502E, and the F lane is transfected with EphB1 Y502F. Top blots were probed for GluN1, and bottom blots were probed for EphB1. The left panels are IP samples pulled down with α-FLAG antibody, and the right panels are lysates. Tubulin was used as a loading control. (D) Experimental approach for panels (E–G) is illustrated. (E) Adult mice underwent unilateral plantar incision. 24 hours following incision, their spinal cords were separated into ipsilateral (+) and contra-lateral (-) sides to injury. Endogenous EphB1 was immunoprecipitated with α-EphB1 antibody from these tissues. Blots were probed for GluN1 and EphB1. EphB1 is expressed in the spinal cord and interacts with the N-methyl-D-aspartate receptor (NMDAR) [25, 61]; therefore, for these experiments we used antibodies against EphB1. (F) Quantification of the effects of unilateral plantar incision on the EphB—NMDAR interaction (*p <* 0.05, Mann—Whitney U test, 8 spinal cords pooled per sample, *n* = 3). (G) Adult mice underwent plantar incision. Mice were given intrathecal injections of either vehicle control (black) or K252b (1 μg, purple) at the time of plantar incision and again 24 hours following incision (*n* = 6 mice for control and *n* = 5 mice for K252b) (for all graphs, *p < 0.05, **p < 0.01, ***p < 0.001, ****p < 0.0001, 1-way or2-way ANOVA followed by Dunnett’s multiple comparison test). (H) Experimental approach for panels (I–K) is illustrated. (I) Intrathecal injections of lentivirus transducing EGFP (black), enhanced yellow fluorescent protein (EYFP)-tagged EphB2 WT (blue), EphB2 Y504E (green), or EphB2 Y504F (red) were made into adult mice. Graph shows quantification of the effects of these injections on mechanical sensitivity in mice 1–4 weeks after injection of the virus (*n* = 8 mice for control before viral injection and *n* = 7 for 1–4 weeks after viral injection; *n* = 8 mice for EphB2 WT; *n* = 8 for EphB2 Y504E before viral injection; *n* = 7 for 2–4 weeks after viral injection; *n* = 8 for EphB2 Y504F). (J) Intrathecal injections of lentivirus transducing EGFP (black), EYFP-tagged EphB2 WT (blue), or EphB2 Y504E (green) were made into adult mice. Graph shows quantification of the effects of these injections on mechanical sensitivity in mice 1–4 weeks after injection of the virus (*n* = 6 mice for control, *n* = 8 mice for EphB2 WT, *n* = 6 for EphB2 Y504E before viral injection and *n* = 4 for 1–4 weeks after viral injection). Bars indicate the period of significant effects on sensitivity in EphB2 WT or Y504E-injected animals. (K) Four weeks after intrathecal injection of lentivirus transducing EGFP (black), EYFP-tagged EphB2 WT (blue), or EphB2 Y504E (green), mice were given intrathecal injections with K252b (1 μg), and effects on mechanical sensitivity were monitored at 1, 3, and 24 hours after injection (*n* = 6 mice for control before K252b injection and *n* = 3 mice for control after K252b injection; *n* = 6 mice for EphB2-WT before K252b injection and 1 hour after K252b injection; *n* = 5 mice for EphB2-WT 3 hours and 24 hours after K252b injection; *n* = 6 mice for EphB2-Y504E). Bars indicate period of significant effects on sensitivity in EphB2 WT or Y504E-injected animals following K252b injection. K252b reduced mechanical sensitivity in EphB2 WT—transduced but not in EphB2 Y504E—transduced mice.

Ephrin-B—EphB signaling to the NMDAR is implicated in pain plasticity, leading to pathological pain states [39, 40]. Given the impact of overexpression of EphBs on NMDAR levels in the dorsal synaptic region of the spinal cord that is associated with pain, we next examined whether the EphB—NMDAR interaction might be enhanced in models of pain. Consistent with previous reports on the role of ephrin-Bs in induction of pain and pain plasticity [40, 41], intrathecal injection of activated ephrin-B2, which activates EphBs and the EphB—NMDAR interaction [11] (Fig 7A, 0.2 μg), induced robust, sustained mechanical hypersensitivity in mice (*p <* 0.0001, ANOVA followed by Dunnett’s multiple comparison test; Fig 7B). Thus. injection of activated ephrin results in hypersensitivity.

Because EphB1 is expressed more highly than EphB2 in the spinal cord and is known to interact with the NMDAR (S7C Fig) [23, 42, 43], we tested whether the region homologous to Y504 in EphB2 was also important for the previously described EphB1–NMDAR interaction. EphB1 contains a homologous tyrosine residue at Y502 (Fig 1D). Therefore, we hypothesized that the charge of this residue might regulate the EphB1–NMDAR interaction. Consistent with this model, compared to FLAG-EphB1, WT co-expression of FLAG-EphB1 Y502E with GluN1 and GluN2B in HEK293T cells resulted in increased coimmunoprecipitation, while the interaction was significantly reduced when FLAG-EphB1 Y502F was coexpressed (Fig 7C and S7A Fig). We next asked whether EphB1 is also phosphorylated at Y502. HEK293T cells were transfected with either EphB1 WT or EphB1 Y502F, and lysates were probed with the p*Y504 antibody. Consistent with selective phosphorylation of Y502, only WT EphB1 was recognized by the p*Y504 antibody (S7B Fig). These findings suggest that the ability of both EphB1 and EphB2 to interact with the NMDAR is mediated by phosphorylation of homologous tyrosine residues (Y502 and Y504 respectively).

Next, we asked whether injury-induced mechanical hypersensitivity might induce the EphB—NMDAR interaction (Fig 7D). Consistent with a role for the EphB—NMDAR interaction, unilateral plantar incision (a mouse model sensitive to NMDAR receptor blockade [44]) resulted in a significant increase in the coimmunoprecipitation of the GluN1 subunit of the NMDAR with EphB1 (51.3 ± 26.4-fold increase, *p <* 0.05, Mann—Whitney U test, 8 hemispinal cords pooled per sample, *n* = 3, Fig 7E and 7F). In the spinal cord, detection of phosphorylation of EphB1 and EphB2 was difficult because of the small amounts of tissue available from each animal and levels of EphB expression. However, by pooling samples we were able to observe modest effects on EphB2 Y504 and Y502 phosphorylation in spinal cord samples after injury (S7C Fig). Importantly, the increase in the EphB—NMDAR interaction was only observed in tissue from the same side as the injury, indicating that this effect is likely specific to the injury paradigm.

Next, to begin to test whether extracellular phosphorylation might be important for the behavioral response to injury, we tested whether blockade of extracellular kinase activity with the membrane-impermeant drug K252b might rescue injury-induced mechanical hypersensitivity (Fig 7D). We used intrathecal infusion, a method allowing for local infusion of drugs and widely used clinically to allow for spinal cord—selective treatment [45]. Intrathecal injections of K252b (1 μg) at the time of plantar incision significantly reduced mechanical hypersensitivity for up to 24 hours after incision in adult mice (Fig 7G). These results support a model in which inhibiting extracellular phosphorylation can reduce pain signaling and mechanical hypersensitivity behavior (S8 Fig).

To begin to test whether the EphB—NMDAR interaction might play a role in pathological pain, we asked whether inducing the EphB—NMDAR interaction with transduction of EYFP-tagged EphB2 WT or Y504E that results in increased levels of NMDAR in the spinal cord (Fig 6) might result in mechanical hypersensitivity (Fig 7H). Intrathecal injection of lentivirus transducing either EYFP-tagged EphB2 WT or Y504E resulted in a long-lasting enhancement of mechanical sensitivity (Fig 7I and 7J). Consistent with in vivo and in vitro immunostaining and physiological data (Figs 5 and 6), we find that intrathecal injections of the Y504F mutant did not result in a long-lasting increase in mechanical hypersensitivity (Fig 7I). Thus, transduction of EYFP-tagged EphB2 WT or Y504E results in mechanical hypersensitivity and increased levels of GluN1 in regions of the spinal cord linked to pathological pain (Figs 6 and 7I).

### Role of extracellular phosphorylation in EphB-induced mechanical hypersensitivity

Injections of lentivirus transducing WT EphB2 and EphB2-Y504E resulted in long-lasting mechanical hypersensitivity, even 2 months after virus injection (Fig 7I and 7J). To test whether these effects are due to phosphorylation of Y504, we asked next whether inhibition of extracellular phosphorylation might block the effects of EphB2 expression. If mechanical hypersensitivity depends on phosphorylation of Y504, then blocking extracellular phosphorylation should only reduce or rescue mechanical hypersensitivity in mice transduced with WT but not in those with Y504E EYFP-tagged EphB2. To avoid potential effects of inhibiting the EphB—NMDAR interaction outside of the spinal cord [45], intrathecal injections of K252b were made near the site of viral injection. Remarkably, intrathecal injection of K252b resulted in rapid (<3 hours) and sustained (>24 hours) reversal of mechanical hypersensitivity compared to control levels when injected into mice overexpressing WT EYFP-tagged EphB2 (*p <* 0.01, 2-way ANOVA followed by Dunnett’s multiple comparison test; Fig 7J and 7K). Importantly, the rescue of mechanical hypersensitivity to control levels in EphB2 WT—transduced mice (Fig 7K) was found in animals having experienced increased levels of pain for 2 months.

We next conducted a series of controls. Although we did not observe evidence of action of K252b outside of the spinal cord, and intrathecal infusion of drugs is used clinically to avoid side effects seen with other injection methods [45], the drug used to block the interaction (K252b) could act elsewhere in the brain. However, injection of K252b had no effects in control EGFP-expressing mice (Fig 7K). Next, to control for effects of K252b injection outside of the spinal cord and to demonstrate the specific action of this compound on EphB2 with an intact extracellular phosphorylation site, we asked whether K252b might alter the mechanical hypersensitivity in mice expressing the constitutively interacting EphB2 Y504E mutant. Importantly, in mice expressing the EYFP-tagged EphB2 Y504E construct, injection of K252b did not block mechanical hypersensitivity (Fig 7K). Thus, K252b, which inhibits extracellular phosphorylation of Y504, is acting selectively within the spinal cord in WT EphB2-overexpressing mice to block mechanical hypersensitivity. Taken together, these findings support a model wherein the EphB—NMDAR interaction mediated by extracellular phosphorylation of EphB cFN3 induces amplification in pain signaling, causing mechanical hypersensitivity (S8 Fig).

## Discussion

Regulation of cell function and animal behavior by phosphorylation of the intracellular portions of proteins has long been appreciated. Here we show that the ligand-induced phosphorylation of the extracellular domains of proteins may have similarly important impacts on synaptic function and animal behavior. Extracellular tyrosine phosphorylation of EphB2 occurs on a specific well-conserved residue within the FN3 repeat domain. We show that the direct extracellular interaction between EphBs and NMDARs regulates recruitment of NMDARs to synaptic sites and that phosphorylation of Y504 on EphB2 is likely to impact the maintenance of injury-induced mechanical hypersensitivity. Taken together, our findings suggest that extracellular phosphorylation of EphB cFN3 is a critical regulator of synaptic NMDAR function and has implications for understanding synaptic plasticity and disease, especially pathological pain.

### Ligand-inducible extracellular phosphorylation mediates protein–protein interaction

Phosphorylation of protein ectodomains can regulate *Drosophila* wing development, neurite outgrowth, osteoclast migration, and aggregation of amyloid beta [46–49]; however, these events appear to occur largely constitutively and are not known to be regulated by cell—cell signaling. In contrast, phosphorylation of Y504 on EphB2 is induced by ephrin-B and results in the recruitment of NMDAR to synaptic sites. The activity that mediates extracellular phosphorylation of EphB2 is present in ACSF of cultured neurons after eprhin-B2 stimulation and requires ATP. Importantly, ample ATP is found at synaptic terminals [50] and ATP can be released into the extracellular space by synaptic or secretory vesicles [51, 52]. Together with recent work demonstrating that a wide variety of proteins are constitutively extracellularly phosphorylated [4, 5] our data suggest a model in which, similar to intracellular tyrosine phosphorylation, inducible extracellular tyrosine phosphorylation of specific amino acids can regulate extracellular domain—mediated protein—protein interactions.

Intracellular phosphorylation sites enable protein—protein interactions with specific amino acid motifs. Similarly, the conserved region surrounding the Y504 residue on EphB2 appears to define a new extracellular interaction domain. Based on homology, we could accurately predict that EphA8 would also interact with the NMDAR. In contrast, the corresponding regions of EphA3 and EphA4 that do not co-IP with the NMDAR are less homologous to EphB2 [11], although we have not ruled out the possiblity that they may also become phosphorylated. In addition, mutation of the homologous amino acid on EphB1 (Y502) can drive increased or reduced interactions with the NMDAR. These data suggest that the motif XpYVXQVR may be important for interactions between the extracellular domains of proteins. Phosphoprotein databases provide additional examples of phosphorylated tyrosine residues with unknown function in homologous regions of FN3 domains of proteins linked to synaptogenesis, axon guidance, and target recognition, such as Sidekicks and DSCAML1 (S1F Fig). It will be important to determine whether phosphorylation of the FN3 domain of other proteins also regulates protein—protein interactions and whether extracellular phosphorylation is an underappreciated mechanism contributing to the development and function of the nervous system and synapse.

How might extracellular phosphorylation enable protein—protein interaction? In the intracellular space, changes in the charge of tyrosine residues in specific amino acid motifs control the binding of specific SH2 domain—containing molecules [1]. Interestingly, the EphB2 Y504F constructs fail to interact with the NMDAR but are likely not functioning in a dominant negative manner. These findings are consistent with a model in which the EphB—NMDAR interaction is mediated by charge because phenylalanine is not a positively charged amino acid. The role of charge is difficult to test, as positively charged amino acids are bulky and may affect protein—protein interactions in other ways, such as steric hindrance. However, a dominant negative mutant should be able to be designed using surface charge analysis of the region surrounding Y504. It is likely that phosphorylation of the Y504 residue would alter the secondary structure or interaction surface of the FN3 domain. Supporting this idea, analysis of the crystal structures of Eph family members indicates that Y504 of EphB2 is exposed and suggests that the cFN3 of Eph family members, but not the N-terminal FN3 (nFN3) domain, might have a pocket for binding to other molecules [53]. Indeed, the crystal structures of ligand-unbound EphA4 and ligand-bound EphA4 indicate that ligand binding results in changes to the structure of cFN3 [54]. These data suggest that phosphorylation of Y504 could modify FN3 structure. Consistent with this model, phosphorylation of Y504 in EphB2 is necessary and sufficient to induce binding to the NMDAR, and we find that homologous FN3 domains and tyrosine residues can be phosphorylated. However, detailed structural analysis will be required to resolve these questions. FN3 domains of other synaptic proteins contain a homologous residue, suggesting that extracellular phosphorylation is a novel mechanism that may mediate many events related to synaptic function and behavior.

### Extracellular phosphorylation controls GluN2B-containing NMDAR function in young neurons

Extracellular phosphorylation of EphB2 at Y504 stabilizes GluN2B-containing NMDARs on the cell surface and drives the accumulation of GluN2B-containing NMDARs at synaptic sites in young neurons. During development, GluN2B-containing NMDARs predominate at synaptic sites, and the presence of GluN2B-containing NMDARs at synapses can enhance synaptic plasticity [55]. Interestingly, phosphorylation of EphB2 at Y504 is down-regulated as synapses mature, and the proportion of GluN2B-containing NMDARs is reduced. EphB2 regulates a number of other aspects of GluN2B-containing NMDAR function. EphB2 activation can drive the Src kinase—dependent phosphorylation of GluN2B on specific tyrosine residues that prevent AP2-mediated internalization [56]. The kinase-active EphB2 reduces calcium-dependent inactivation of GluN2B-containing (but not GluN2A-containing) NMDAR currents [14]. However, in adult animals, EphBs are required for normal synaptic levels of both GluN2A and GluN2B, suggesting a role for additional developmentally regulated mechanisms. It remains to be determined whether the ability of EphB2 to interact with GluN2A-containing NMDAR relies on the same Y504 site or a different mechanism. Further indication of the importance of extracellular modification of the EphB2 receptor is suggested by findings in human disease models. Disruption of the EphB—NMDAR interaction in models of Alzheimer disease [19] or in NMDAR encephalitis [18] that results in reduced surface levels of NMDAR may involve the FN3 domain of the EphB2 protein, further supporting the importance of understanding phosphorylation of these domains.

### Mechanism of EphB extracellular phosphorylation

The kinase regulating the phosphorylation of Y504 on EphB2 remains to be identified. Interestingly, Y504 is not phosphorylated on the kinase-dead version of EphB2 (EphB2 KD) implicating the kinase domain of EphB2. It is not likely, though, that the ephrin-B—induced extracellular phosphorylation of EphB2 Y504 is phosphorylated directly by the EphB2 kinase because (1) p*Y504 requires extracellular ATP, (2) p*Y504 occurs on the cell surface, (3) p*Y504 is blocked by a membrane-impermeable kinase inhibitor K252b, (4) extracellular treatment with phosphatases augments the interaction, (5) an activity that can phosphorylate Y504 is secreted following ephrin-B treatment, and (6) although K252b effectively inhibited p*Y504, it failed to block EphB2 kinase activity. However, while the EphB2 kinase is unlikely directly responsible for phosphorylating Y504, given that p*Y504 requires ephrin-B activation of EphB2, it is likely that the EphB2 kinase participates in the signal transduction pathway necessary for p*Y504. Further work will be needed to identify the genuine Y504 kinase. One interesting set of candidates is the recently identified family of extracellular kinases (such as Fam20C) that function in the secretion pathway [46]. A second class of candidates is represented by the tyrosine kinase VLK, which can be secreted from platelets and is an essential gene [6, 7]. The mechanism of action of this family is unknown, but since these kinases can function extracellularly, they might be good candidates for the genuine p*Y504 kinase, which appears to be secreted.

### Extracellular phosphorylation of EphB regulates pathological pain

While disorders like Alzheimer disease and NMDAR-encephalitis may be linked to pathological reduction of the EphB—NMDAR interaction [18, 21], induction of hypersensitivity and pain can occur by enhancing EphB-dependent effects on NMDAR function [39]. Indeed, increases in the amount of ephrin-B ligand have been reported in the spinal cord in pain models. Consistent with a role for the EphB—NMDAR interaction are the following: (1) MK801 blocks the effect of ephrin-B1 on pathological pain [40], (2) local intrathecal application of EphB-Fc blocks pathological pain [24, 42], and (3) Nav1.8+ nociceptive sensory neuron (DRG)-specific ephrin-B2 knockout regulates inflammatory and neuropathic pain [57]. These findings suggest that in the spinal cord and periphery, EphB-dependent modulation of NMDAR function drives NMDAR-dependent hyperexcitability of spinal circuits, causing pathological pain associated with traumatic nerve injury and cancer-induced nerve damage [25, 41, 58, 59]. Consistent with these previous findings, we show that a single intrathecal injection of activated ephrin-B2 results in hypersensitivity.

In addition to functions in the induction of pathological pain, we find that the EphB—NMDAR interaction also appears to play an important role in maintaining long-lasting mechanical hypersensitivity. Virally induced expression of WT EphB2 in dorsal horn neurons results in sustained mechanical hypersensitivity. Importantly, injection of K252b 2 months after induction by an EphB2-expressing virus rapidly alleviates this mechanical hypersensitivity. These findings suggest that inhibition of extracellular phosphorylation might provide a target suitable to alleviate more chronic pain states.

In animals injected with virus transducing the EphB2 Y504E point mutant that induces a constitutive, ligand-independent interaction with the NMDARs, injection of K252b did not influence this established mechanical hypersensitivity. This underscores the specific, local effect of extracellular EphB2 phosphorylation. Consistent with clinical practices [45], our findings indicate that intrathecally injected K252b is acting selectively within the spinal cord on WT EphB2 overexpressing cells and not likely targeting regions outside of the spinal cord. In addition to blocking EphB2-EYFP—induced mechanical hypersensitivity, K252b reduced incision-induced mechanical hypersensitivity, suggesting a key role for EphB—NMDAR interactions in this context, a notion supported by biochemical findings in our models. Although K252b is not itself a suitable compound for further development, these data suggest that extracellular phosphorylation and inhibition of the EphB—NMDAR interaction may provide potential therapeutic targets for the mitigation of pathological pain. In summary, extracellular phosphorylation of EphB mediates the direct extracellular EphB—NMDAR interaction to direct NMDARs to synaptic sites in a variety of neuronal types and regulates behaviorally relevant events such as pathological pain.

## Materials and methods

### Animals

All animal procedures were approved by the Institutional Animal Care and Use Committee of Thomas Jefferson University, the University of Pennsylvania, the University of Texas at Dallas, and the University of Arizona and were in accordance with International Association for the Study of Pain guidelines (protocol numbers: 01286 and 01797 TJU, 14–04 UTD, 802645 UPenn and 09–115 UA). Embryonic day 17 (E17) to E18 Long—Evans rats (Charles River) were used for preparing dissociated cortical neural culture as previously described [13, 14]. Postnatal day 30 wild-type CD1 mice (Charles River) were used for synaptosome preparation as previously described [14]. Male ICR mice (Harlan) were used for all behavioral studies. Mice were used for behavioral experiments starting at 8–12 weeks of age.

### NG108 cell culture and peptide identification

For LC-MS/MS analysis, NG108 cells were transfected with FLAG-tagged EphB2 receptor, treated with ephrin-B1, and lysed. Samples immunoprecipitated with α-FLAG antibody were separated by SDS-PAGE, the gels were stained with Coomassie Blue, and the EphB2 band was excised and digested with trypsin in-gel. After enrichment of phosphopeptides using TiO_2_, LC-MS/MS analysis was conducted (Thermo Fisher Scientific LTQ-Orbitrap).

### Expression constructs

Generation of FLAG-tagged full-length EphB2, truncated EphB2 (fEphB2 Tr), and kinase-dead (KD; K663R) EphB2 were previously described [11]. Generation of EphB2-EYFP was previously described [60]. Single amino acid point mutations to Y481 and Y504 were introduced using sequence-specific primers and site-directed mutagenesis (Stratagene, La Jolla, CA). EphB2 expression constructs were generated based on published sequences. Expression of each EphB2 version used was validated using RT-PCR from mouse brain cDNA. In some cases, the RT-PCR products were validated by sequencing. FLAG- and EYFP-tagged EphB2 expression constructs gave similar results in assays tested. Lentiviruses were produced and purified by the Gene Therapy Program at the Penn Vector Core facility of the University of Pennsylvania.

### HEK293T cells and primary neuronal culture

HEK293T cells were transfected using the calcium phosphate method. Dissociated cortical neurons were prepared from embryonic day 17 (E17) to E18 rats. Dissociated spinal cord neurons were prepared from E14.5 rats. Cultured neurons were transfected at DIV 3 or 14 using Lipofecatmine 2000. Immunoprecipitations and western blot analysis were performed as previously described with small changes [11]. Cell-surface biotinylations were performed as previously described [14, 38].

### Immunocytochemistry, immunohistochemistry, imaging, and analysis

For phosphotyrosine surface staining, live neurons were incubated for 10 minutes at room temperature in PBS with α-phosphotyrosine, then washed and fixed. For synaptic NMDAR staining, cells were fixed and permeabilized with 0.1% saponin at room temperature. For immunohistochemical analysis, spinal cord tissues were frozen in OCT compound and cut into sections and then fixed and stained. Images were obtained using Leica TCS SP5 confocal scanning microscopy and Leica TCS SP8 STED microscopy. Analysis was done using NIH ImageJ. All data shown as mean ± SEM.

### Generation of a phosphorylation-specific antibody

Phospho-specific antibodies against α-EphB2 Y504 were generated against a phosphopeptide of the sequence Ac-CKGLKAGAI-pY-VGQVRA-NH_2_ (Covance, Denver, PA).

### Synaptosome preparation

Synaptosomes were prepared as previously described [14].

### Electrophysiology

Electrophysiological recordings from DIV 21–23 cultured rat cortical neurons were performed using whole-cell patch methods as previously described [14]. Evoked currents were generated by light stimulation (470 nanometers) of neurons expressing channelrhodopsin-2.

### Behavioral testing and drug administration

Behavioral testing and drug administration were performed as described previously [35].

## Supporting information

S1 Fig Identification of phosphorylated tyrosine in cFN3 of EphB2 extracellular region; related to Fig 1. (A-D) Validation of co-immunoprecipitation for mass spectrometry. Lysates of NG108 cells expressing FLAG-tagged EphB2 treated without or with clustered Fc or ephrin-B1 were immunoprecipitated with anti-FLAG or PY99. Western blotting with α-FLAG or α-PY99 showed that transfected FLAG-tagged EphB2 (arrow heads in A and D) as well as other protein (arrow in B) were phosphorylated on tyrosine residues after treatment with clustered ephrin B1- Fc. The total amount of EphB2 was decreased after ephrin-B1 treatment (arrow head in C). (E-F) Alignment of cFN3 in EphB2 with Eph Family Proteins and FN3-containing molecules. (E) Alignment of EphB2 cFN3 domain in vertebrates and Eph in invertebrates using ClustalW2 software. EphB2 Y504 (red) corresponds to a conserved tyrosine residue whereas Y481 (blue) is only conserved in mammals (gray). (F) Alignment of EphB2 cFN3 domain with other FN3- containing proteins with phosphorylated tyrosine residues that are comparable to EphB2 Y504. (G) Untransfected cultured rat cortical neurons (DIV 6–10) were treated with ephrin-B2 (eB2) or control reagents (Fc) for 45–60 minutes. Endogenous EphB2 was immunoprecipitated using α-EphB2 antibodies and blots were probed with α-EphB2 p*Y504 pre-absorbed with non-phospho- or phospho-EphB2 Y504 peptide (top blots) or α-EphB2 extracellular region (bottom blots). Preabsorption of α-EphB2 Y504 with phospho- but not non-phospho-peptide eliminates the EphB2 p*Y504 signals induced by ephrin-B2-treament. Similar results were obtained in three separate experiments. (H) Top blot shows HEK293T lysates probed with a phospho-specific antibody generated against EphB2 Y504 (α-EphB2 p*Y504). Second blot shows same lysates probed for EphB2. Third blot shows lysates probed for EphB2 pY662 (EphB2 kinase activity). Bottom blot shows lysates probed for tubulin loading control. Lanes were loaded with lysates of HEK293T cells transfected with control, (WT) full-length EphB2, or full-length EphB2 Y481F. (I) Full blots of synaptosomes probed for α-p*Y504 in Fig 1G.
(PDF)

S2 Fig Extracellular tyrosine phosphorylation of EphB2 is not induced during sample preparation, but is induced by ephrin-B2 in cultured cortical neurons; related to Fig 2. (A) *In vitro* kinase assay revealed that autophosphorylation of intracellular fragments of EphB2 containing tyrosine kinase domain induced by ATP is inhibited by 4 μM PD161570 (PD) in culture medium (Neurobasal medium + B27 supplement) or MOPS buffer, but not inhibited by membrane-impermeable kinase inhibitor K252b (10 uM). All lanes are from the same blot with irrelevant intervening lanes removed. (B) Although pretreatment with PD161570 (PD) inhibits both extracellular and intracellular tyrosine phosphorylation of EphB2 induced by ephrin-B2-treatment, presence of this inhibitor following lysis did not affect phosphorylation of EphB2. (C) Sucrose treatment inhibited internalization of ephrin-B2-Fc in cultured cortical neurons. To strip ephrin-B2-Fc binding to membrane surface and only detect the internalized ephrin-B2-Fc, neurons were incubated with 0.2 M acetic acid and 0.5 M NaCl on ice followed by washes with ACSF. Intracellular without or with surface ephrin-B2-Fc was probed for human IgG (ephrinB2-Fc) or actin (control). (D) Quantification of effects of pretreatment with 450 mM sucrose significantly inhibited incorporation of ephrin-B2-Fc into cultured cortical neurons (p < 0.05, Mann-Whitney U-test, n = 3). (E) Regular immunocytochemistry (ICC) and surface staining of living neurons with α-actin revealed weak background staining in a living condition. (F) Ephrin-B2-treated conditioned ACSF in cultured cortical neurons was treated without (-) or with (+) 73–75°C for 20–30 min (+) prior to being used for kinase assay. Upper blots were probed for phospho-tyrosine. Bottom blots were probed for EphB2. (G) Quantification of tyrosine phosphorylation of EphB2-Fc. Heat treatment significantly reduced tyrosine kinase activity present in ephrin-B2-treated conditioned ACSF in cultured cortical neurons (*p < 0.05, Mann-Whitney U-test, n = 4).
(PDF)

S3 Fig Blockade of extracellular phosphorylation does not affect EphB2 Y662 phosphorylation; related to Fig 3. (A) Quantification of the effects of ephrin-B2 treatment after blockade of extracellular kinase activity with K252b on the phosphorylation of Y662 in neurons (*p<0.01, ANOVA followed by Fisher’s; n = 5 experiments for each condition). No significant difference was found (p > 0.05) between ephrin-B2 treated samples. (B) Untransfected cultured rat cortical neurons (DIV 6–7) were treated with ephrin-B2 (+) or control reagents (-) for 45–60 minutes and either control (C) or ATPγS applied to the extracellular space (1 μM). EphB2 was immunoprecipitated and blots were probed for GluN1 (top blots). Lower blots are input controls showing GluN1 staining, EphB2 pY662, EphB2, and tubulin. (C) Quantification of the ratio of EphB2 pY662 to total EphB2. (****p < 0.001, ANOVA followed by Fisher’s, n = 5 experiments). (D) Quantification of the ratio of GluN1 pulled down with EphB2 and GluN1 levels in the input (***p < 0.005, ANOVA followed by Fisher’s, n = 5 experiments).
(PDF)

S4 Fig The charge of EphB2 Y504 controls EphB-NMDAR interaction but not ephrin-B2 binding; related to Fig 4. (A) Quantification of ephrin-B2-Fc binding to surface FLAG-tagged EphB2 WT and Y504E and Y504F mutants (n = 25, 25 and 25 cells from three independent experiments for WT, Y504E and Y504F, respectively, p > 0.05 ANOVA). (B) Regular immunocytochemistry (ICC) and surface staining of living HEK293T cells expressing FLAG-tagged EphB2 WT with α-actin revealed weak background staining in a living condition. (C) Images of HEK293T without transfection, incubated with ephrin-B2 for 45 minutes, stained as in (Fig 4B). (D) Images of HEK293T transfected FLAG-tagged EphB2 WT, incubated with control (human Fc) for 45 minutes, stained as in (B). (E) Immunoprecipitation (IP) of FLAG-tagged EphB2 with α-EphB2 antibodies from HEK293T. All lanes are transfected with HA-GluN1 and GluN2B. Control lane has only HA-GluN1 and GluN2B alone, WT lane transfected with FLAG-EphB2, E lane is transfected with FLAG-EphB2 Y481E, F lane is transfected with FLAG-EphB2 Y481F. Top blots were probed for HA (GluN1), bottom blots probed for FLAG (EphB2). Left panels are IP samples, right panels are lysates. (F) Quantification of ratio of co-IPed GluN1 to total GluN1 in input. IP of FLAG-tagged EphB2 mutants revealed that neither EphB2 Y481E nor Y481F affect the ability of EphB2 pull-down HA-GluN1. (*p < 0.01 for WT, p = 0.196 for Y481E and p = 0.191 for Y481F compared to WT, ANOVA followed by Fisher’s PLSD test, n = 5).
(PDF)

S5 Fig EphB2 tyrosine 504 and NMDAR reciprocally regulate the surface retention of the EphB-NMDAR complex; related to Fig 4. (A) Model of how extracellular phosphorylation at Y504 modulates EphB receptor surface retention and the EphB-NMDAR interaction. (B and C) EphB2 Y504 regulates surface retention of NMDAR in HEK293T cells. Co-expression of EphB2 Y504E with GluN1 and GluN2B significantly increased the fraction of surface GluN1 receptors on the plasma membrane compared to HEK293T cells transfected with GluN1 and GluN2B alone or with EphB2 WT or Y504F (*p < 0.05, ANOVA followed by Fisher’s PLSD test, n = 6). In contrast, co-expression of non-phosphorylatable Y504F mutant EphB2 receptors that fail to interact with NMDARs resulted in a significant decrease of GluN1 receptors on the cell surface compared to WT EphB2 (p = 0.0272). (D and E) Co-expression of NMDAR increases the surface retention of EphB2 Y504E mutant in HEK293T cells (*p < 0.05, n = 6, Fisher’s PLSD test). (F and G) Cultured neurons (DIV 7) infected with EphB2 YFP or Y504 mutants at DIV 2 were used to detect the surface level of EphB2 WT or Y504 mutants. In neurons, Y504E mutants are retained on the plasma membrane (p = 0.2175 for Y504E and p = 0.5814 for Y504F, compared with WT, Fisher’s PLSD test).
(PDF)

S6 Fig Effects of EphB2 Y504 mutation on the mEPSC frequency and amplitude; related to Fig 5. (A) Effects of EphB2 Y504 mutation on the mEPSC frequency recorded at -65 mV in mature cultured cortical neurons. Whole-cell patch-clamp recordings from DIV 21–23 cultured rat cortical neurons transfected with EGFP and control, EphB2-YFP-WT, Y504E or Y504F. There were no significant differences between control (9 neurons) and EphB2 WT (p = 0.0541, ANOVA followed by Fisher’s PLSD test, 10 neurons), Y504E (p = 0.259, ANOVA followed by Fisher’s) or Y504F (p = 0.473, ANOVA followed by Fisher’s, 14 neurons) mutants in mean mEPSC frequency. Data are shown as mean ± s.e.m. (B) Example whole-cell patch-clamp recordings from cortical neurons in each condition showing the mEPSC frequency. (C) Quantification of mean mEPSC amplitude. An increase in mEPSC amplitude recorded at -65 mV was observed with overexpression of EphB2 WT (p < 0.0001, ANOVA followed by Fisher’s, 2534 events from 11 neurons) and Y504E (p = <0.0001, ANOVA followed by Fisher’s, 1983 events from 11 neurons) and Y504F (p = 0.0002, ANOVA followed by Fisher’s, 2433 events from 15 neurons) in compared with control neurons (1366 events from 10 neurons). In addition, mEPSC amplitude of neurons expressing Y504F was significantly lower than neurons expressing EphB2 WT or Y504E (*p = 0.0048 and < 0.0001, ANOVA followed by Fisher’s). Data are shown as mean ± s.e.m. (D) Sample traces of whole cell patch-clamp recording at -65 mV shows that mEPSC amplitude of neurons expressing EphB2 WT and Y504E, but not Y504F are higher than control neurons. (E) Effects of overexpression of EphB2 WT and Y504 mutants without or with APV on the mean amplitude of mEPSC (15–25 msec after the mEPSC peak) in mature cortical rat DIV 21–23 neurons. Neurons were transfected at DIV 14. Overexpression of EphB2 WT or Y504E significantly increased amplitude of the NMDAR dependent component of mEPSC compared to control or Y504F mutants (*p < 0.01, ANOVA followed by Fisher’s, n = 421, 308, 758, 818, 349, 469, 541 and 293 events for Control, Control + APV, EphB2 WT, EphB2 WT + APV, Y504E, Y504E + APV, Y504F and Y504F + APV, respectively). (F) Cumulative probability histogram of mEPSC amplitude at 15–25 msec after the mEPSC peak for Y504E and Y504F mutants before (dark shading) and after (light shading) NMDAR blockage with 50 μM APV. Inset, Mean traces of mEPSCs after NMDAR blockage with APV. Vertical Scale bar = 10 pA, horizontal scale bar = 10 msec.
(PDF)

S7 Fig Phosphorylation of EphB1 Y502 is recognized by p*Y504 antibody and control the EphB1-NMDAR interaction; related to Fig 7. (A) Co-immunoprecipitation (Co-IP) of GluN1 and EphB1 with α-GluN1 antibody from HEK293T. All lanes are transfected with HA-GluN1 and GluN2B. Control lane is transfected with GFP, WT lane is transfected with EphB1 WT, E lane is transfected with EphB1 Y502E, F lane is transfected with EphB1 Y502F. Top blots were probed for FLAG, bottom blots probed for GluN1. Left panels are IP samples pulled down with α-GluN1 antibody, right panels are lysates. Tubulin is used as loading control. (B) Top blot shows HEK293T lysates probed with a phospho-specific antibody α-EphB2 p*Y504. Middle blot shows same lysates probed for EphB1. Bottom blot shows lysates probed for tubulin loading control. Lanes were loaded with lysates of HEK293T cells transfected with either GFP Control, EphB1 WT, or EphB1 Y502F. (C) Adult mice underwent unilateral plantar incision. 24 hours following incision spinal cord was separated into ipsilateral (+) and contra-lateral (-) sides to injury. Endogenous EphB2 or EphB1 were immunoprecipitated with α-EphB2 or α-EphB1 antibodies, respectively from these tissues. Blots were probed with phospho-specific antibody α-p*Y504 and EphB.
(PDF)

S8 Fig Models of how extracellular tyrosine phosphorylation of EphBs cFN3 may affect NMDAR function and pain behaviors through regulating EphB-NMDAR interaction; related to Fig 7. (A) Model of effects of extracellular phosphorylation of EphB cFN3 on the surface retention, accumulation and function of GluN2B-containing NMDARs and pain plasticity. (B) Under physiological conditions, ephrin-B-induced extracellular tyrosine phosphorylation of EphB cFN3 is not required for pain sensitivity through regulating EphB-NMDAR interaction, Ca2+-dependent signaling, and NMDAR-dependent gene expression. (C) Injury increases the level of ephrin-B and/or EphB expression. In turn, this induces NMDAR-dependent pain plasticity potentially leading to pathological pain. (D) Potential approach for therapies to alter pain plasticity: In chronic malignancy-induced or neuropathic pain diseases, EphB-dependent enhancement of NMDAR activity may be prevented by blocking the extracellular tyrosine phosphorylation of EphB cFN3 using ecto-kinase inhibitors such as K252b.
(PDF)

S1 Table Identification of EphB2 phosphorylation sites; related to Fig 1. (DOCX)

S1 Text Extended experimental procedures. (DOCX)

S1 Data Supporting data file for Hanamura et al. (XLSX)

## Abbreviations

ACSF: artificial cerebrospinal fluid
AU: arbitrary unit
cFN3: C-terminal FN3
CIP: calf intestinal alkaline phosphatase
Cys: cysteine-rich domain
D-APV: D-2-amino-5-phosphonovalerate
DIV: day in vitro
EGFP: enhanced green fluorescent protein
EPSC: excitatory postsynaptic current
EYFP: enhanced yellow fluorescent protein
FL: full length
FN3: fibronectin type III
IP: immunoprecipitation
JM: juxtamembrane domain
KD: kinase dead
K–S: Kolmogorov–Smirnov
LBD: ligand-binding domain
LC-MS/MS: liquid chromatography tandem mass spectrometry
LV: lentivirus
mEPSC: miniature excitatory postsynaptic current
MS/MS: tandem mass spectrometry
nFN3: N-terminal FN3
NMDAR: N-methyl-D-aspartate receptor
pA: picoampere
PDZ: PSD-95/DLG1/ZO-1 domain
PY99: α-phosphotyrosine
RNAi: RNA interference
Ro25: Ro 25–6981
RTK: receptor tyrosine kinase
SAM: sterile α-motif
shRNA: short hairpin RNA
STED: stimulated emission depletion
TK: tyrosine kinase domain
TM: transmembrane domain
TR: truncated
VLK: vertebrate lonesome kinase
WT: wild type
