# Correction: Extracellular phosphorylation of a receptor tyrosine kinase controls synaptic localization of NMDA receptors and regulates pathological pain

**DOI:** 10.1371/journal.pbio.3001452

**Published:** 2021-11-09

**Authors:** Kenji Hanamura, Halley R. Washburn, Sean I. Sheffler-Collins, Nan L. Xia, Nathan Henderson, Dipti V. Tillu, Shayne Hassler, Daniel S. Spellman, Guoan Zhang, Thomas A. Neubert, Theodore J. Price, Matthew B. Dalva

The S1 Data File is incorrect. The correct data file is provided here.

## Supporting information

S1 Data Supporting data file for Hanamura et al. (XLSX)

## References

[1] Hanamura K, Washburn HR, Sheffler-Collins SI, Xia NL, Henderson N, Tillu DV, et al. (2017) Extracellular phosphorylation of a receptor tyrosine kinase controls synaptic localization of NMDA receptors and regulates pathological pain. PLoS Biol 15(7): e2002457. 10.1371/journal.pbio.2002457 28719605 PMC5515392

