# Supplementary material for: Extracellular phosphorylation of a receptor tyrosine kinase controls synaptic localization of NMDA receptors and regulates pathological pain
Source: PLoS Biol. 2017 Jul 18;15(7):e2002457. doi: 10.1371/journal.pbio.2002457 (PMC5515392; doi:10.1371/journal.pbio.2002457)
Supplement: S1 Fig — (A-D) Validation of co-immunoprecipitation for mass spectrometry. Lysates of NG108 cells expressing FLAG-tagged EphB2 treated without or with clustered Fc or ephrin-B1 were immunoprecipitated with anti-FLAG or PY99. Western blotting with α-FLAG or α-PY99 showed that transfected FLAG-tagged EphB2 (arrow heads in A and D) as well as other protein (arrow in B) were phosphorylated on tyrosine residues after treatment with clustered ephrin B1- Fc. The total amount of EphB2 was decreased after ephrin-B1 treatment (arrow head in C). (E-F) Alignment of cFN3 in EphB2 with Eph Family Proteins and FN3-containing molecules. (E) Alignment of EphB2 cFN3 domain in vertebrates and Eph in invertebrates using ClustalW2 software. EphB2 Y504 (red) corresponds to a conserved tyrosine residue whereas Y481 (blue) is only conserved in mammals (gray). (F) Alignment of EphB2 cFN3 domain with other FN3- containing proteins with phosphorylated tyrosine residues that are comparable to EphB2 Y504. (G) Untransfected cultured rat cortical neurons (DIV 6–10) were treated with ephrin-B2 (eB2) or control reagents (Fc) for 45–60 minutes. Endogenous EphB2 was immunoprecipitated using α-EphB2 antibodies and blots were probed with α-EphB2 p*Y504 pre-absorbed with non-phospho- or phospho-EphB2 Y504 peptide (top blots) or α-EphB2 extracellular region (bottom blots). Preabsorption of α-EphB2 Y504 with phospho- but not non-phospho-peptide eliminates the EphB2 p*Y504 signals induced by ephrin-B2-treament. Similar results were obtained in three separate experiments. (H) Top blot shows HEK293T lysates probed with a phospho-specific antibody generated against EphB2 Y504 (α-EphB2 p*Y504). Second blot shows same lysates probed for EphB2. Third blot shows lysates probed for EphB2 pY662 (EphB2 kinase activity). Bottom blot shows lysates probed for tubulin loading control. Lanes were loaded with lysates of HEK293T cells transfected with control, (WT) full-length EphB2, or full-length EphB2 Y481F. (I) Full blots of sy [file pbio.2002457.s001.pdf]

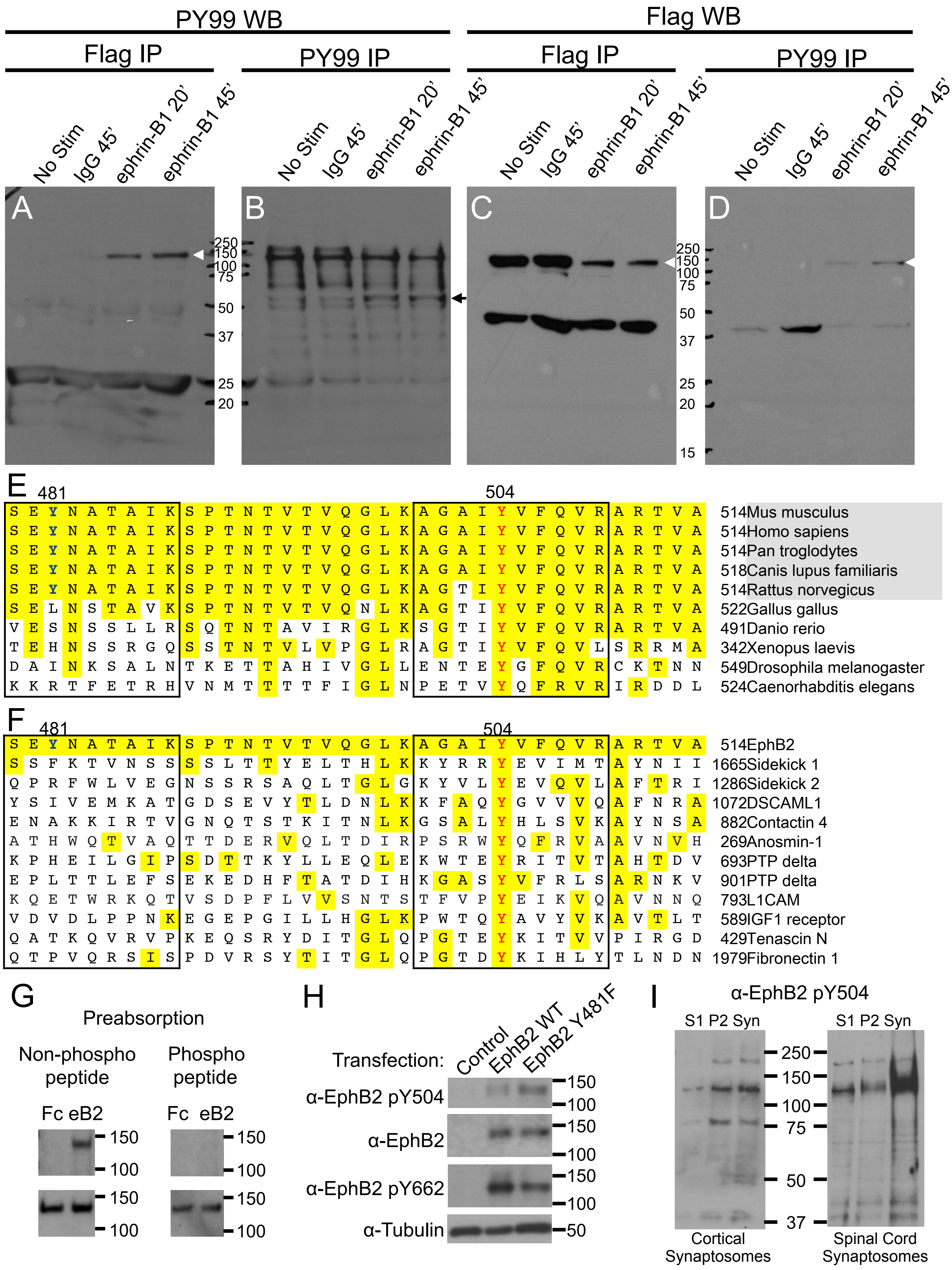
PY99 WB
Flag WB
Flag IP
PY99 IP
Flag IP
PY99 IP
No Stim
IgG 45'
ephrin-B1 20'
ephrin-B1 45'
A
B
C
D
250
150
100
75
50
37
25
20
15
E
481
504
S E Y N A T A I K S P T N T V T V Q G L K A G A I Y V F Q V R A R T V A 514Mus musculus
S E Y N A T A I K S P T N T V T V Q G L K A G A I Y V F Q V R A R T V A 514Homo sapiens
S E Y N A T A I K S P T N T V T V Q G L K A G A I Y V F Q V R A R T V A 514Pan troglodytes
S E Y N A T A I K S P T N T V T V Q G L K A G A I Y V F Q V R A R T V A 518Canis lupus familiaris
S E Y N A T A I K S P T N T V T V Q G L K A G T I Y V F Q V R A R T V A 514Rattus norvegicus
S E L N S T A V K S P T N T V T V Q N L K A G T I Y V F Q V R A R T V A 522Gallus gallus
V E S N S S L L R S Q T N T A V I R G L K S G T I Y V F Q V R A R T V A 491Danio rerio
T E H N S S R G Q S S T N T V L V P G L R A G T I Y V F Q V L S R R M A 342Xenopus laevis
D A I N K S A L N T K E T T A H I V G L L E N T E Y G F Q V R C K T N N 549Drosophila melanogaster
K K R T F E T R H V N M T T T T F I G L N P E T V Y Q F R V R I R D D L 524Caenorhabditis elegans
F
481
504
S E Y N A T A I K S P T N T V T V Q G L K A G A I Y V F Q V R A R T V A 514EphB2
S S F K T V N S S S S L T T Y E L T H L K K Y R R Y E V I M T A Y N I I 1665Sidekick 1
Q P R F W L V E G N S S R S A Q L T G L G K Y V L Y E V Q V L A F T R I 1286Sidekick 2
Y S I V E M K A T G D S E V Y T L D N L K K F A Q Y G V V V Q A F N R A 1072DSCAML1
E N A K K I R T V G N Q T S T K I T N L K G S A L Y H L S V K A Y N S A 882Contactin 4
A T H W Q T V A Q T T D E R V Q L T D I R P S R W Y Q F R V A A V N V H 269Anosmin-1
K P H E I L G I P S D T T K Y L L E Q L E K W T E Y R I T V T A H T D V 693PTP delta
E P L T T L E F S E K E D H F T A T D I H K G A S Y V F R L S A R N K V 901PTP delta
K Q E T W R K Q T V S D P F L V V S N T S T F V P Y E I K V Q A V N N Q 793L1CAM
V D V D L P P N K E G E P G I L L H G L K P W T Q Y A V Y V K A V T L T 589IGF1 receptor
Q A T K Q V R V P K E Q S R Y D I T G L Q P G T E Y K I T V V P I R G D 429Tenascin N
Q T P V Q R S I S P D V R S Y T I T G L Q P G T D Y K I H L Y T L N D N 1979Fibronectin 1
G
Preabsorption
Non-phospho peptide
Phospho peptide
Fc eB2
Fc eB2
150
100
H
Transfection:
Control
EphB2 WT
EphB2 Y481F
α-EphB2 pY504
α-EphB2
α-EphB2 pY662
α-Tubulin
150
100
50
I
α-EphB2 pY504
S1 P2 Syn
S1 P2 Syn
250
150
100
75
50
37
Cortical Synaptosomes
Spinal Cord Synaptosomes
