# Supplementary figures and images for: Extracellular phosphorylation of a receptor tyrosine kinase controls synaptic localization of NMDA receptors and regulates pathological pain
Source: PLoS Biol. 2017 Jul 18;15(7):e2002457. doi: 10.1371/journal.pbio.2002457 (PMC5515392; doi:10.1371/journal.pbio.2002457)

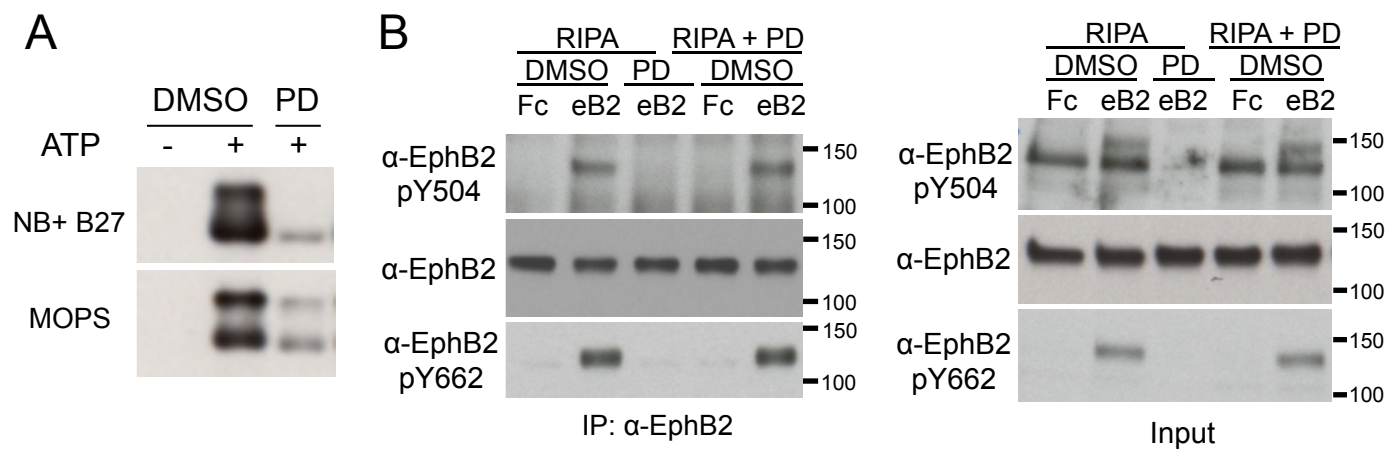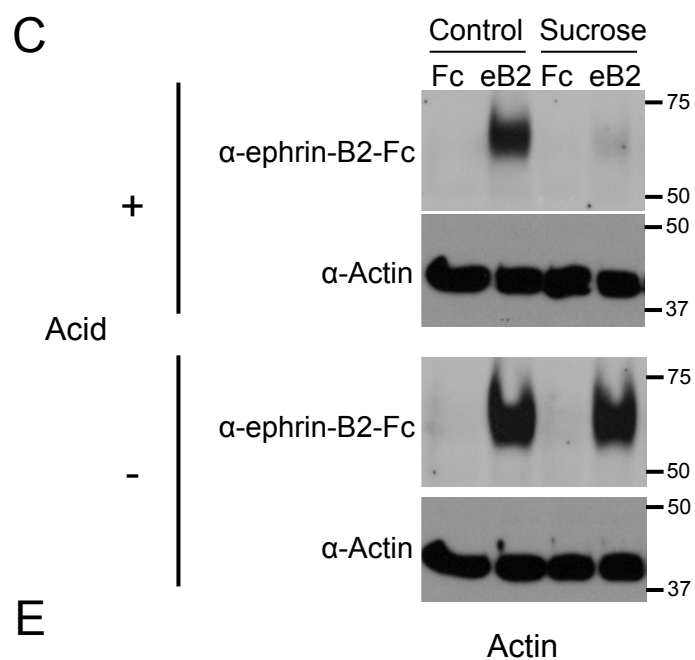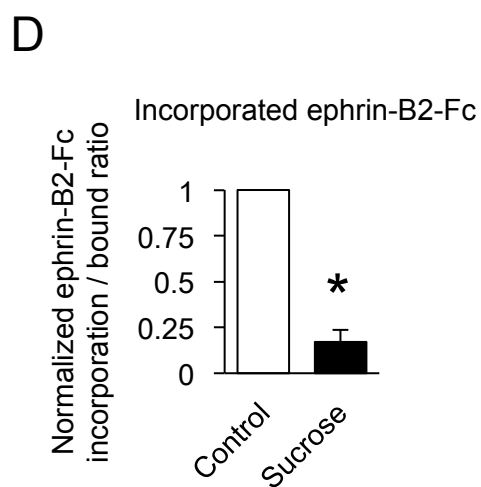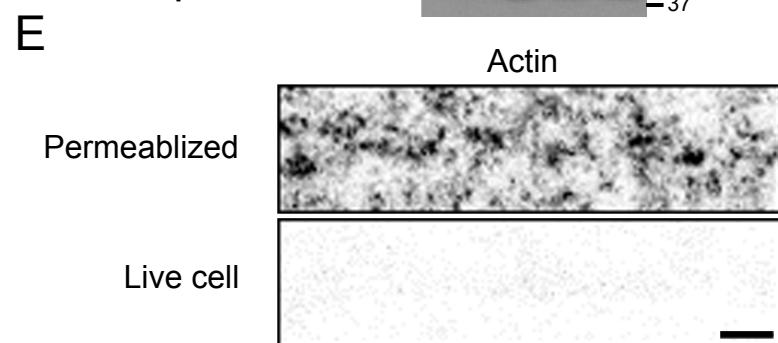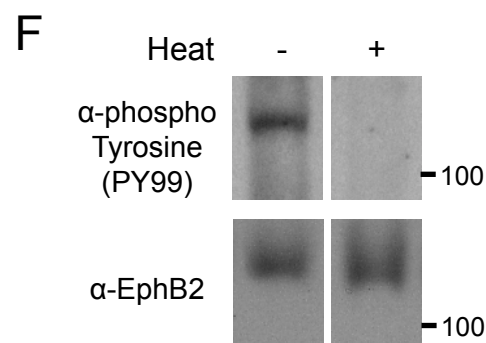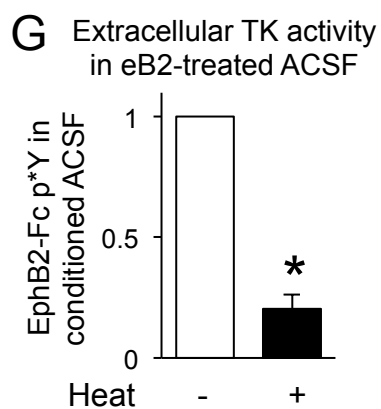

Supplement: S2 Fig — (A) In vitro kinase assay revealed that autophosphorylation of intracellular fragments of EphB2 containing tyrosine kinase domain induced by ATP is inhibited by 4 μM PD161570 (PD) in culture medium (Neurobasal medium + B27 supplement) or MOPS buffer, but not inhibited by membrane-impermeable kinase inhibitor K252b (10 uM). All lanes are from the same blot with irrelevant intervening lanes removed. (B) Although pretreatment with PD161570 (PD) inhibits both extracellular and intracellular tyrosine phosphorylation of EphB2 induced by ephrin-B2-treatment, presence of this inhibitor following lysis did not affect phosphorylation of EphB2. (C) Sucrose treatment inhibited internalization of ephrin-B2-Fc in cultured cortical neurons. To strip ephrin-B2-Fc binding to membrane surface and only detect the internalized ephrin-B2-Fc, neurons were incubated with 0.2 M acetic acid and 0.5 M NaCl on ice followed by washes with ACSF. Intracellular without or with surface ephrin-B2-Fc was probed for human IgG (ephrinB2-Fc) or actin (control). (D) Quantification of effects of pretreatment with 450 mM sucrose significantly inhibited incorporation of ephrin-B2-Fc into cultured cortical neurons (p < 0.05, Mann-Whitney U-test, n = 3). (E) Regular immunocytochemistry (ICC) and surface staining of living neurons with α-actin revealed weak background staining in a living condition. (F) Ephrin-B2-treated conditioned ACSF in cultured cortical neurons was treated without (-) or with (+) 73–75°C for 20–30 min (+) prior to being used for kinase assay. Upper blots were probed for phospho-tyrosine. Bottom blots were probed for EphB2. (G) Quantification of tyrosine phosphorylation of EphB2-Fc. Heat treatment significantly reduced tyrosine kinase activity present in ephrin-B2-treated conditioned ACSF in cultured cortical neurons (*p < 0.05, Mann-Whitney U-test, n = 4). (PDF) [file pbio.2002457.s002.pdf]

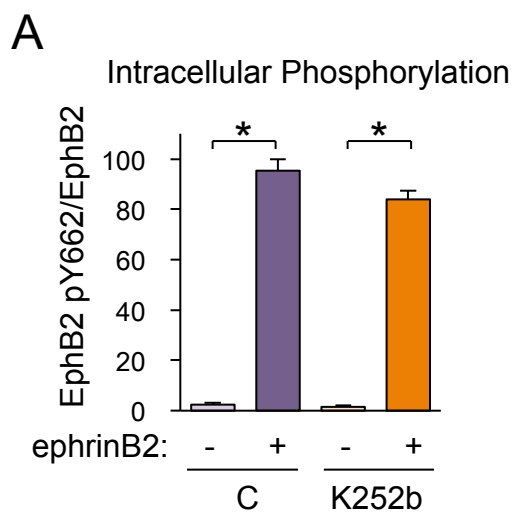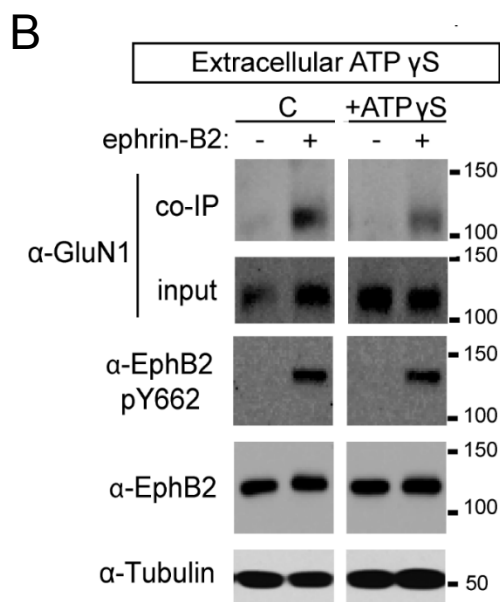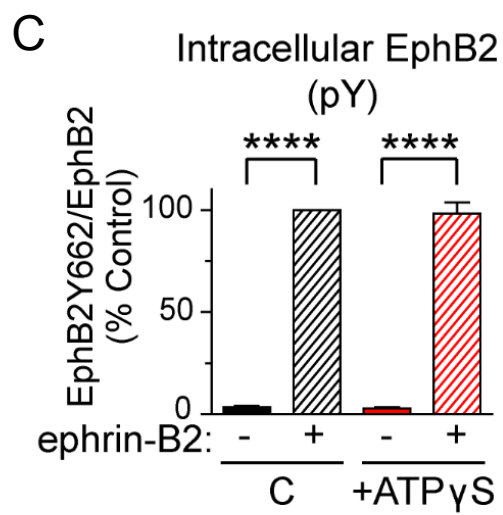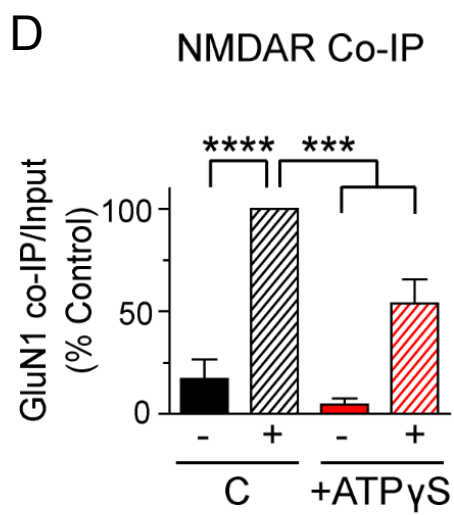

Supplement: S3 Fig — (A) Quantification of the effects of ephrin-B2 treatment after blockade of extracellular kinase activity with K252b on the phosphorylation of Y662 in neurons (*p<0.01, ANOVA followed by Fisher’s; n = 5 experiments for each condition). No significant difference was found (p > 0.05) between ephrin-B2 treated samples. (B) Untransfected cultured rat cortical neurons (DIV 6–7) were treated with ephrin-B2 (+) or control reagents (-) for 45–60 minutes and either control (C) or ATPγS applied to the extracellular space (1 μM). EphB2 was immunoprecipitated and blots were probed for GluN1 (top blots). Lower blots are input controls showing GluN1 staining, EphB2 pY662, EphB2, and tubulin. (C) Quantification of the ratio of EphB2 pY662 to total EphB2. (****p < 0.001, ANOVA followed by Fisher’s, n = 5 experiments). (D) Quantification of the ratio of GluN1 pulled down with EphB2 and GluN1 levels in the input (***p < 0.005, ANOVA followed by Fisher’s, n = 5 experiments). (PDF) [file pbio.2002457.s003.pdf]

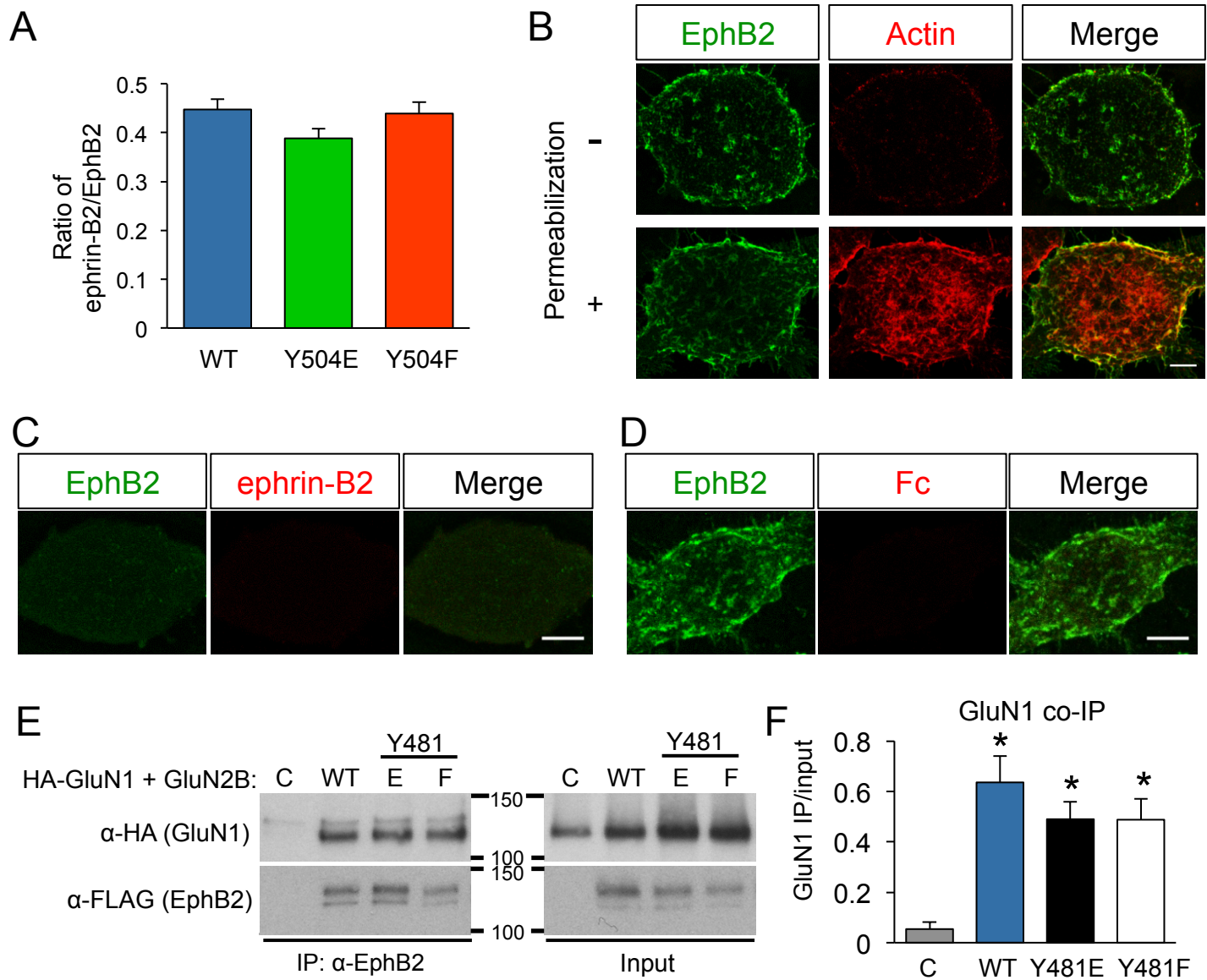

Supplement: S4 Fig — (A) Quantification of ephrin-B2-Fc binding to surface FLAG-tagged EphB2 WT and Y504E and Y504F mutants (n = 25, 25 and 25 cells from three independent experiments for WT, Y504E and Y504F, respectively, p > 0.05 ANOVA). (B) Regular immunocytochemistry (ICC) and surface staining of living HEK293T cells expressing FLAG-tagged EphB2 WT with α-actin revealed weak background staining in a living condition. (C) Images of HEK293T without transfection, incubated with ephrin-B2 for 45 minutes, stained as in (Fig 4B). (D) Images of HEK293T transfected FLAG-tagged EphB2 WT, incubated with control (human Fc) for 45 minutes, stained as in (B). (E) Immunoprecipitation (IP) of FLAG-tagged EphB2 with α-EphB2 antibodies from HEK293T. All lanes are transfected with HA-GluN1 and GluN2B. Control lane has only HA-GluN1 and GluN2B alone, WT lane transfected with FLAG-EphB2, E lane is transfected with FLAG-EphB2 Y481E, F lane is transfected with FLAG-EphB2 Y481F. Top blots were probed for HA (GluN1), bottom blots probed for FLAG (EphB2). Left panels are IP samples, right panels are lysates. (F) Quantification of ratio of co-IPed GluN1 to total GluN1 in input. IP of FLAG-tagged EphB2 mutants revealed that neither EphB2 Y481E nor Y481F affect the ability of EphB2 pull-down HA-GluN1. (*p < 0.01 for WT, p = 0.196 for Y481E and p = 0.191 for Y481F compared to WT, ANOVA followed by Fisher’s PLSD test, n = 5). (PDF) [file pbio.2002457.s004.pdf]

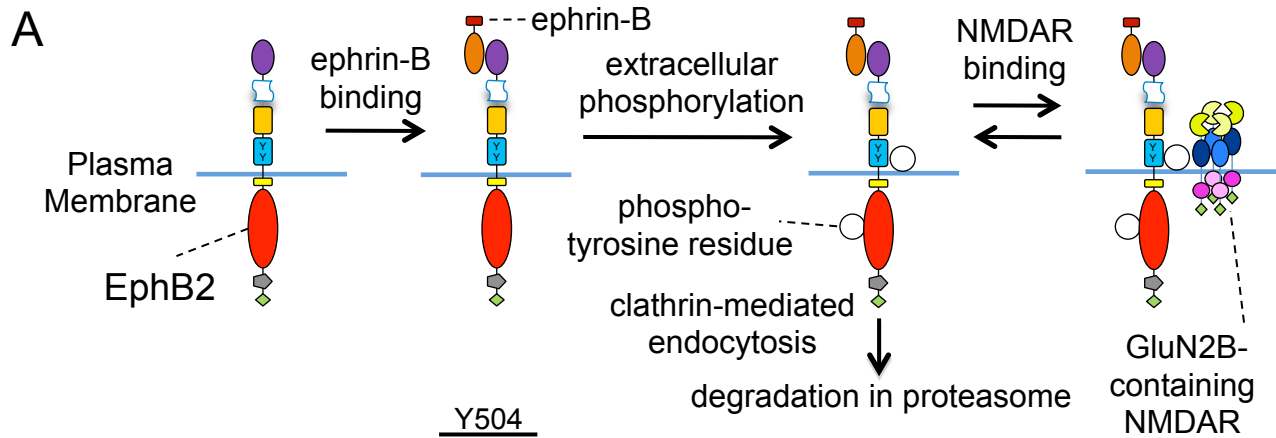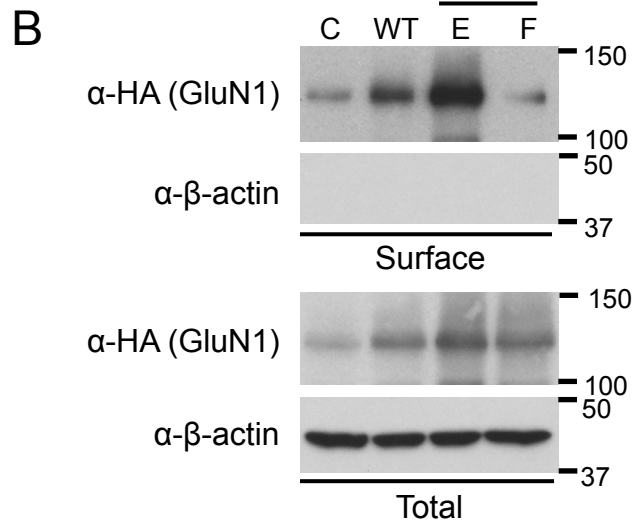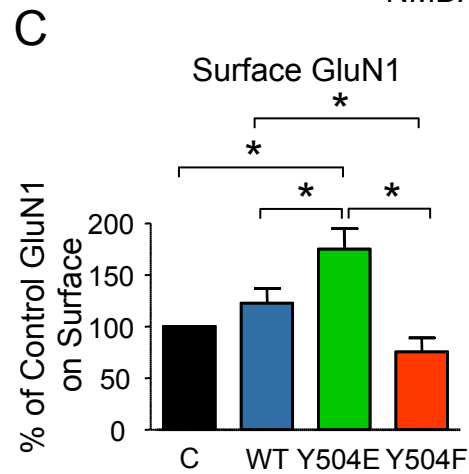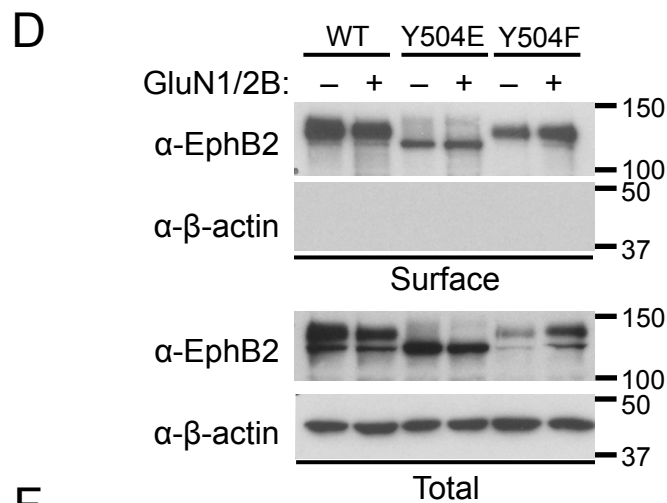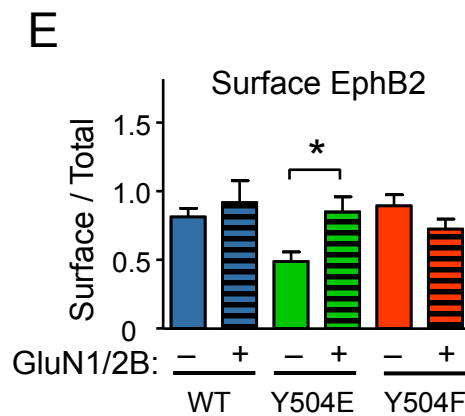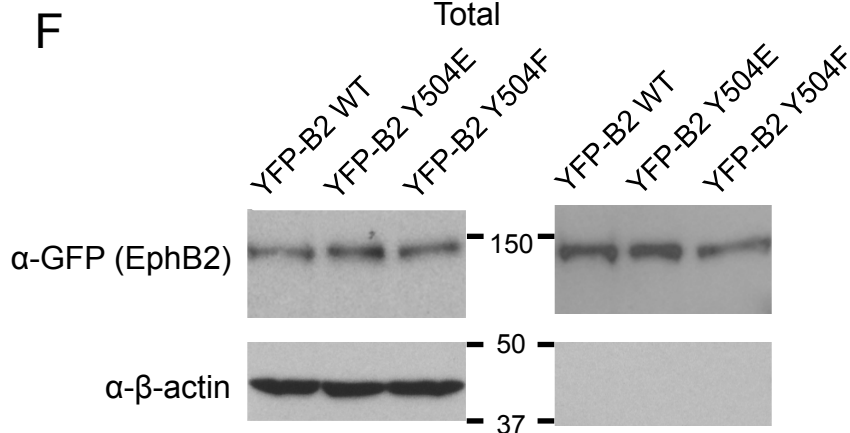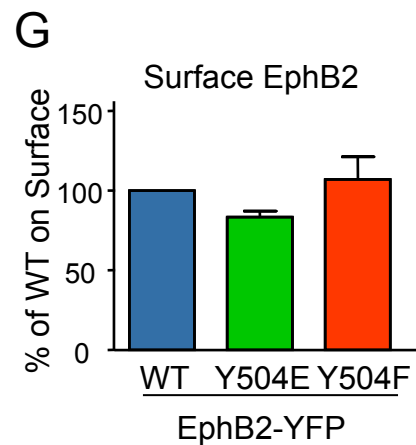

Supplement: S5 Fig — (A) Model of how extracellular phosphorylation at Y504 modulates EphB receptor surface retention and the EphB-NMDAR interaction. (B and C) EphB2 Y504 regulates surface retention of NMDAR in HEK293T cells. Co-expression of EphB2 Y504E with GluN1 and GluN2B significantly increased the fraction of surface GluN1 receptors on the plasma membrane compared to HEK293T cells transfected with GluN1 and GluN2B alone or with EphB2 WT or Y504F (*p < 0.05, ANOVA followed by Fisher’s PLSD test, n = 6). In contrast, co-expression of non-phosphorylatable Y504F mutant EphB2 receptors that fail to interact with NMDARs resulted in a significant decrease of GluN1 receptors on the cell surface compared to WT EphB2 (p = 0.0272). (D and E) Co-expression of NMDAR increases the surface retention of EphB2 Y504E mutant in HEK293T cells (*p < 0.05, n = 6, Fisher’s PLSD test). (F and G) Cultured neurons (DIV 7) infected with EphB2 YFP or Y504 mutants at DIV 2 were used to detect the surface level of EphB2 WT or Y504 mutants. In neurons, Y504E mutants are retained on the plasma membrane (p = 0.2175 for Y504E and p = 0.5814 for Y504F, compared with WT, Fisher’s PLSD test). (PDF) [file pbio.2002457.s005.pdf]

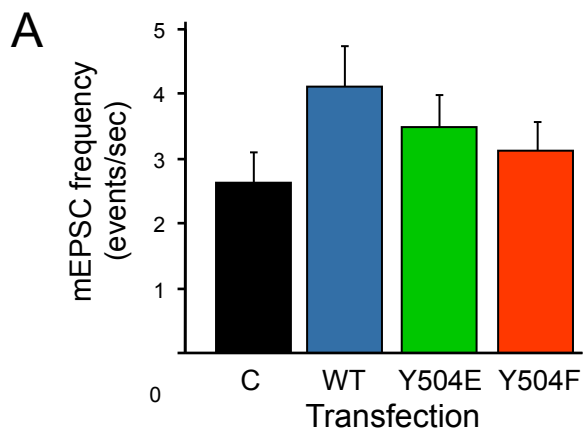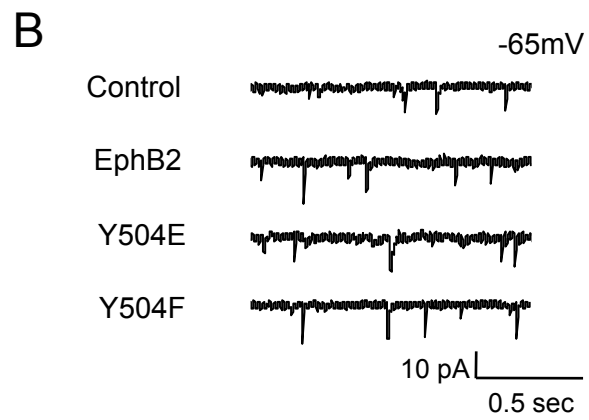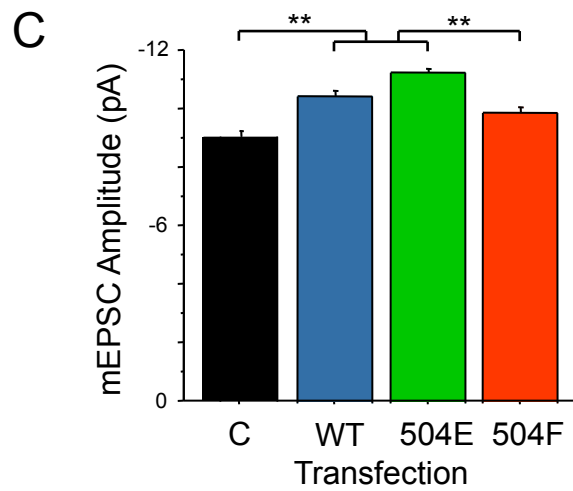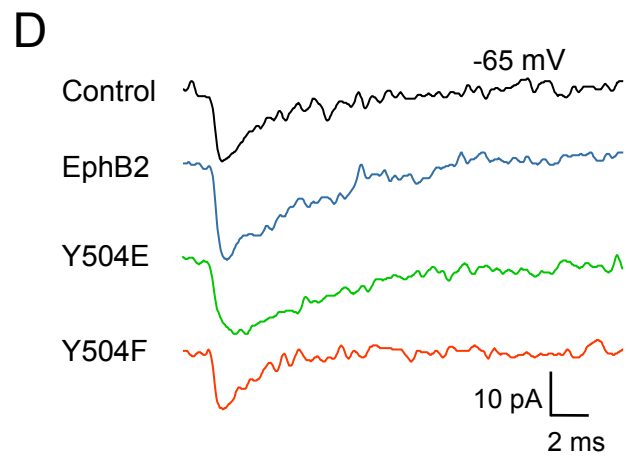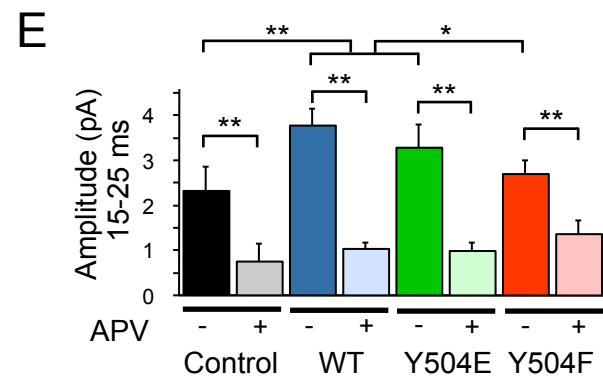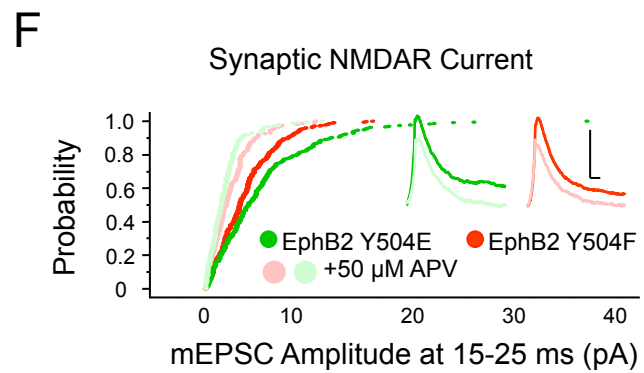

Supplement: S6 Fig — (A) Effects of EphB2 Y504 mutation on the mEPSC frequency recorded at -65 mV in mature cultured cortical neurons. Whole-cell patch-clamp recordings from DIV 21–23 cultured rat cortical neurons transfected with EGFP and control, EphB2-YFP-WT, Y504E or Y504F. There were no significant differences between control (9 neurons) and EphB2 WT (p = 0.0541, ANOVA followed by Fisher’s PLSD test, 10 neurons), Y504E (p = 0.259, ANOVA followed by Fisher’s) or Y504F (p = 0.473, ANOVA followed by Fisher’s, 14 neurons) mutants in mean mEPSC frequency. Data are shown as mean ± s.e.m. (B) Example whole-cell patch-clamp recordings from cortical neurons in each condition showing the mEPSC frequency. (C) Quantification of mean mEPSC amplitude. An increase in mEPSC amplitude recorded at -65 mV was observed with overexpression of EphB2 WT (p < 0.0001, ANOVA followed by Fisher’s, 2534 events from 11 neurons) and Y504E (p = <0.0001, ANOVA followed by Fisher’s, 1983 events from 11 neurons) and Y504F (p = 0.0002, ANOVA followed by Fisher’s, 2433 events from 15 neurons) in compared with control neurons (1366 events from 10 neurons). In addition, mEPSC amplitude of neurons expressing Y504F was significantly lower than neurons expressing EphB2 WT or Y504E (*p = 0.0048 and < 0.0001, ANOVA followed by Fisher’s). Data are shown as mean ± s.e.m. (D) Sample traces of whole cell patch-clamp recording at -65 mV shows that mEPSC amplitude of neurons expressing EphB2 WT and Y504E, but not Y504F are higher than control neurons. (E) Effects of overexpression of EphB2 WT and Y504 mutants without or with APV on the mean amplitude of mEPSC (15–25 msec after the mEPSC peak) in mature cortical rat DIV 21–23 neurons. Neurons were transfected at DIV 14. Overexpression of EphB2 WT or Y504E significantly increased amplitude of the NMDAR dependent component of mEPSC compared to control or Y504F mutants (*p < 0.01, ANOVA followed by Fisher’s, n = 421, 308, 758, 818, 349, 469, 541 and 293 events for Control, Control + [file pbio.2002457.s006.pdf]

**A**

HEK 293T EphB1 Co-IP

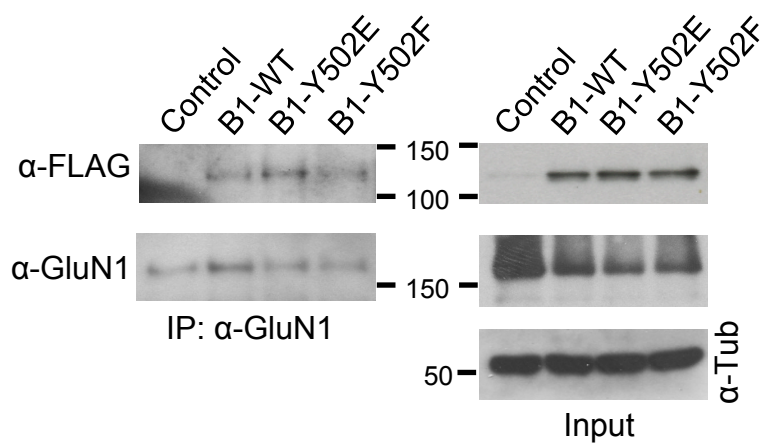

**B**

p\*Y in HEK 293T

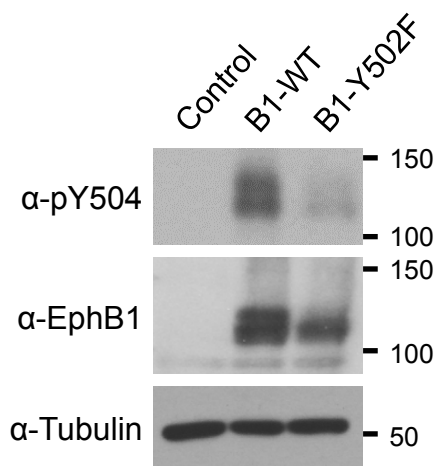

**C**

p\*Y in Spinal Cord

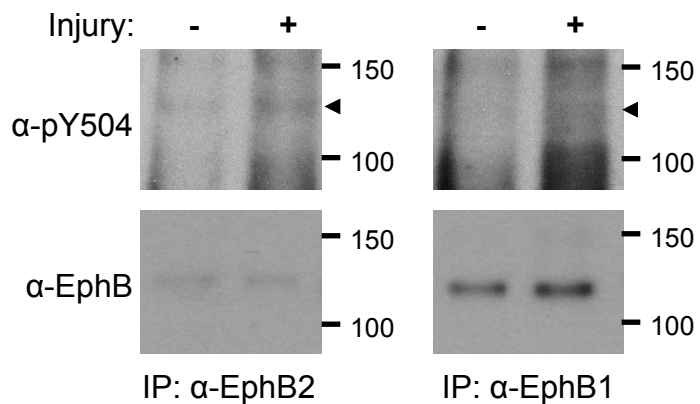

Supplement: S7 Fig — (A) Co-immunoprecipitation (Co-IP) of GluN1 and EphB1 with α-GluN1 antibody from HEK293T. All lanes are transfected with HA-GluN1 and GluN2B. Control lane is transfected with GFP, WT lane is transfected with EphB1 WT, E lane is transfected with EphB1 Y502E, F lane is transfected with EphB1 Y502F. Top blots were probed for FLAG, bottom blots probed for GluN1. Left panels are IP samples pulled down with α-GluN1 antibody, right panels are lysates. Tubulin is used as loading control. (B) Top blot shows HEK293T lysates probed with a phospho-specific antibody α-EphB2 p*Y504. Middle blot shows same lysates probed for EphB1. Bottom blot shows lysates probed for tubulin loading control. Lanes were loaded with lysates of HEK293T cells transfected with either GFP Control, EphB1 WT, or EphB1 Y502F. (C) Adult mice underwent unilateral plantar incision. 24 hours following incision spinal cord was separated into ipsilateral (+) and contra-lateral (-) sides to injury. Endogenous EphB2 or EphB1 were immunoprecipitated with α-EphB2 or α-EphB1 antibodies, respectively from these tissues. Blots were probed with phospho-specific antibody α-p*Y504 and EphB. (PDF) [file pbio.2002457.s007.pdf]

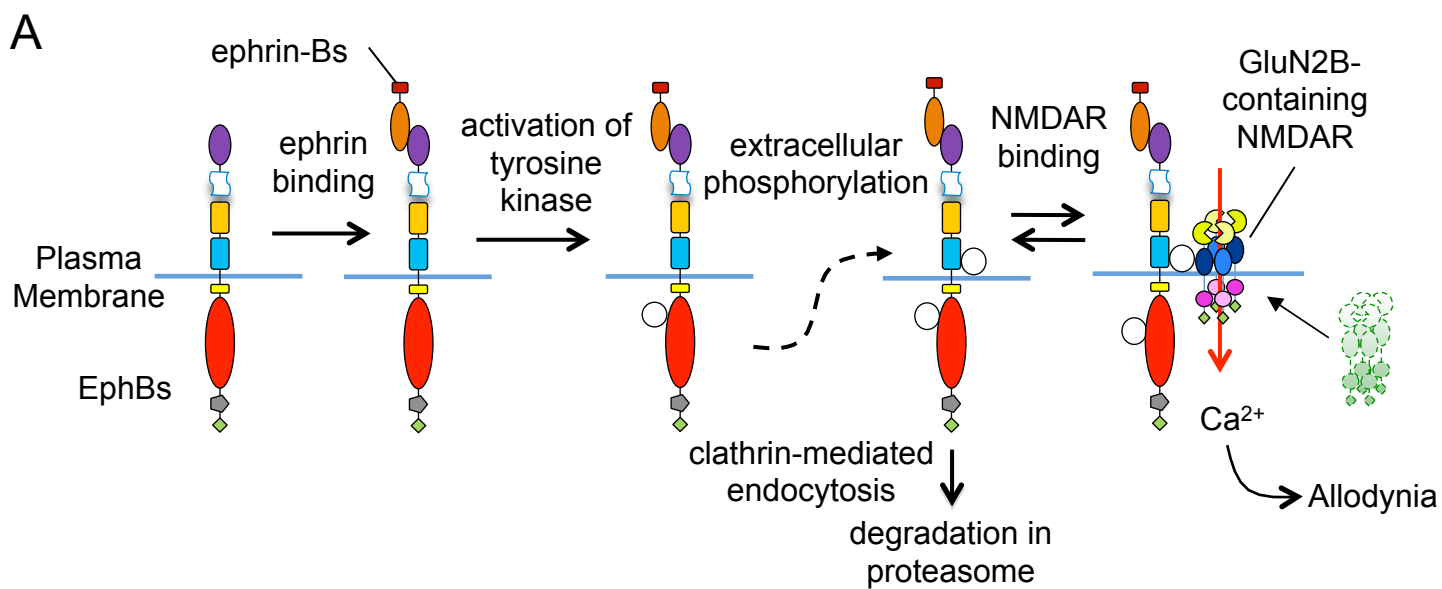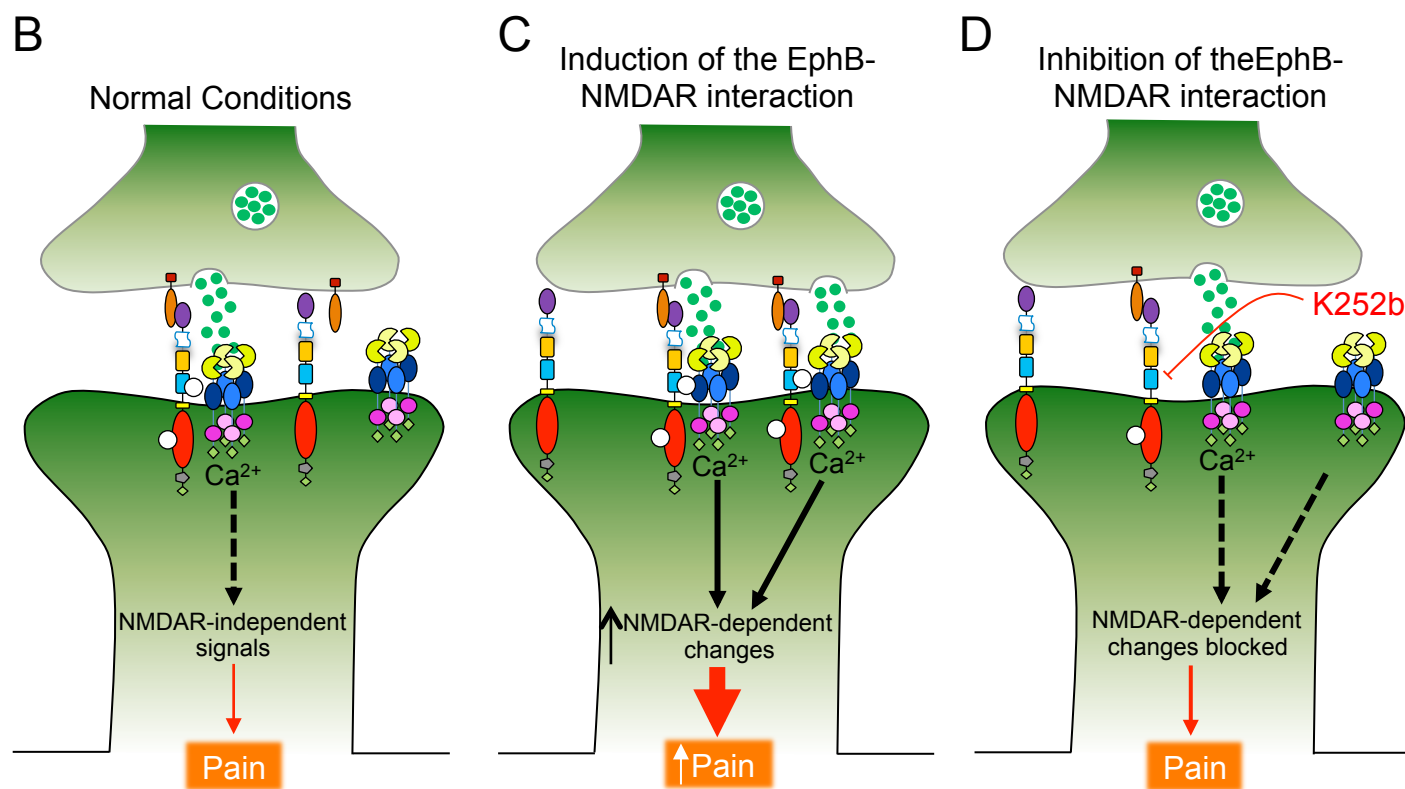

Supplement: S8 Fig — (A) Model of effects of extracellular phosphorylation of EphB cFN3 on the surface retention, accumulation and function of GluN2B-containing NMDARs and pain plasticity. (B) Under physiological conditions, ephrin-B-induced extracellular tyrosine phosphorylation of EphB cFN3 is not required for pain sensitivity through regulating EphB-NMDAR interaction, Ca2+-dependent signaling, and NMDAR-dependent gene expression. (C) Injury increases the level of ephrin-B and/or EphB expression. In turn, this induces NMDAR-dependent pain plasticity potentially leading to pathological pain. (D) Potential approach for therapies to alter pain plasticity: In chronic malignancy-induced or neuropathic pain diseases, EphB-dependent enhancement of NMDAR activity may be prevented by blocking the extracellular tyrosine phosphorylation of EphB cFN3 using ecto-kinase inhibitors such as K252b. (PDF) [file pbio.2002457.s008.pdf]
