# Supplementary material for: Extracellular phosphorylation of a receptor tyrosine kinase controls synaptic localization of NMDA receptors and regulates pathological pain
Source: PLoS Biol. 2017 Jul 18;15(7):e2002457. doi: 10.1371/journal.pbio.2002457 (PMC5515392; doi:10.1371/journal.pbio.2002457)
Supplement: S1 Table — (DOCX) [file pbio.2002457.s009.docx]

**Phosphopeptide Mapping**

| Location | | Sequence | Modifications | Peptide  Mass [Da] | Mascot  Score | Phosphorylation  localized? | Previously  Known? |
| --- | --- | --- | --- | --- | --- | --- | --- |
| Extracellular | 477-487  (cFN3) | ELSE**Y**NATAIK | 1 Phosphotyrosine | 1317.59 | 34 | Yes | unknown |
|  | 500-509  (cFN3) | AGAI**Y**VFQVR | 1 Phosphotyrosine | 1202.58 | 63 | Yes | unknown |
| Intracellular | 573-580  (JM) | ADSEY**T**DK | 1 Phosphoserine/Phosphothreonine | 1007.35 | 21 | Yes |  |
|  |  | AD**S**EY**T**DK | 1 Phosphoserine/Phosphothreonine | 1007.35 | 29 | No |  |
|  |  | AD**S**EYTDK | 1 Phosphoserine/Phosphothreonine | 1007.35 | 50 | Yes |  |
|  |  | ADSE**Y**TDK | 1 Phosphotyrosine | 1007.35 | 42 | Yes | known |
|  | 595-610  (JM) | IYIDPFT**Y**EDPNEAVR | 1 Phosphotyrosine | 2020.88 | 112 | Yes | known |
|  |  | I**Y**IDPFTYEDPNEAVR | 1 Phosphotyrosine | 2020.88 | 79 | Yes | known |
|  |  | IYIDPF**T**YEDPNEAVR | 1 Phosphoserine/Phosphothreonine | 2020.88 | 23 | Yes |  |
|  |  | I**Y**IDPF**T**YEDPNEAVR | 1 Phosphotyrosine,  1 Phosphoserine/Phosphothreonine | 2100.85 | 45 | Yes | known |
|  |  | I**Y**IDPFT**Y**EDPNEAVR | 2 Phosphotyrosine | 2100.85 | 77 | Yes | known |
|  | 770-787  (kinase) | FLEDDTSDPT**Y**TSALGGK | 1 Phosphotyrosine | 1995.84 | 128 | Yes | known |
|  |  | FLEDDT**S**DP**T**YTSALGGK | 1 Phosphoserine/Phosphothreonine | 1995.84 | 100 | No |  |
|  |  | FLEDD**T**SDPTYTSALGGK | 1 Phosphoserine/Phosphothreonine | 1995.84 | 43 | Yes |  |
|  |  | FLEDD**T**SDPT**Y**TSALGGK | 1 Phosphoserine/Phosphothreonine,  1 Phosphotyrosine | 2075.80 | 51 | Yes | known |
|  |  | FLEDDT**S**DPT**Y**TSALGGK | 1 Phosphoserine/Phosphothreonine,  1 Phosphotyrosine | 2075.80 | 71 | Yes | known |
|  |  | FLEDD**TS**DPT**Y**TSALGGK | 1 Phosphoserine/Phosphothreonine,  1 Phosphotyrosine | 2075.80 | 71 | No | known |

**Table S1. Identification of EphB2 Phosphorylation Sites, Related to Figure 1**

LC-MS/MS identified novel phosphorylation site (yellow), Y504 (AGAIYVFQVR) and known phosphorylation site, Y481 (ELSEYNATAIK) in the extracellular portion of the EphB2 (gray) and three known phosphopeptides in juxtamembrane region (JM) (ADSEYTDK and IYIDPFTYEDPNEAVR) and kinase domains (FLEDDTSDPTYTSALGGK). The information about known tyrosine phosphorylation sites is from a database on phosphorylated sites, PhosphoSitePlus (http://www.phosphosite.org/homeAction.do). BOLD RED - indicates localized phosphorylation site, BOLD BLUE - indicates possible sites of unlocalized phosphorylation.
