# Supplementary material for: Extracellular phosphorylation of a receptor tyrosine kinase controls synaptic localization of NMDA receptors and regulates pathological pain
Source: PLoS Biol. 2017 Jul 18;15(7):e2002457. doi: 10.1371/journal.pbio.2002457 (PMC5515392; doi:10.1371/journal.pbio.2002457)
Supplement: S1 Text — (DOCX) [file pbio.2002457.s010.docx]

**EXTENDED EXPERIMENTAL PROCEDURES**

**Animals**

All animal procedures were approved by the Institutional Animal Care and Use Committee of Thomas Jefferson University (01286 and 01797), University of Pennsylvania (802645), University of Texas at Dallas (14-04), and The University of Arizona (09-115) and were in accordance with International Association for the Study of Pain guidelines. Embryonic day 17 (E17) to E18 Long Evans rats (Charles River) were used for preparing dissociated cortical neural culture as previously described [1, 2]. Postnatal day 30 wild-type CD1 mice (Charles River) were used for synaptosome preparation as previously described [2]. Male ICR mice (Harlan) were used for all behavioral studies. Mice were used for behavioral experiments starting at 8-12 weeks of age.

**Expression Constructs**

Generation of FLAG-tagged full length EphB2 WT, truncated FLAG-EphB2 (fEphB2 Tr) and FLAG-tagged kinase dead (KD; K663R) EphB2 were previously described [3]. Single amino acid point mutations to Y481 and Y504 were introduced using sequence specific primers and site-directed mutagenesis (Stratagene, La Jolla, CA). Sequences used were: EphB2 Y504F, 5’-GCCTCAAAGCCGGTGCCATCTTTGTCTTCCAGGTGCGG-3’; EphB2 Y504E, 5’-GCCTCAAAGCCGGTGCCATCGAAGTCTTCCAGGTGCGG-3’; EphB2 Y481F, 5’-ATGAGAAGGAGCTAAGTGAGTTCAACGCCACGGCCATA-3’; EphB2 Y481E, 5’-ATGAGAAGGAGCTAAGTGAGGAGAACGCCACGGCCATA-3’. Forward and reverse oligonucleotides were synthesized (Integrated DNA Technologies, Coralville, IA). Generation of EphB2-YFP was previously described [4]. AgeI and MfeI sites were added to EphB2-YFP using site-directed mutagenesis and sequence specific primers: EphB2-YFP_AgeI, 5’-AAACCGGTTTACCGTGGAAGAAACCCTGATG-3’; EphB2-YFP MfeI, 5’-TTAATCCAATTGGAGTGACAGAGCAGCAGGGAC -3’. Then point mutations at Y504 were introduced using site-directed mutagenesis. PCR products were ligated into the pFUGW vector using Age1 (bp3860) and EcoR1 (bp4609) sites. Expression of each EphB2 version used was validated using RT-PCR from mouse brain cDNA. In some cases, the RT-PCR products were validated by sequencing. FLAG- and EYFP-tagged EphB2 expression constructs gave similar results in assays tested. Lentiviruses were produced and purified by the Gene Therapy Program Penn Vector Core Facility at the University of Pennsylvania (UPenn Vector Core).

Generation of FLAG-tagged full length EphB1 WT, Y502E, and Y502F occurred via site-directed mutagenesis. pDONR223-EPHB1 plasmid was obtained from Addgene. Addition of the FLAG tag and single amino acid point mutations to Y502 were introduced using sequence specific primers and site-directed mutagenesis. Sequences used were: FLAG-tagged EphB1, 5’-

GCAGTGGCTGCGATGGAAgattataaagatgatgatgataaaGAAACGTTAATGGACACC-3’; EphB1 Y502E, 5’-GGGCTGCGGCCcGGCATGGTAgAaGTGGTACAGGTG-3’; EphB1 Y502F, 5’-GGGCTGCGGCCcGGCATGGTcTtTGTGGTACAGGTG-3’. Forward and reverse oligonucleotides were synthesized (Eurofins Genomics). PCR products were inserted into the pFUGW vector using Gateway LR recombination (Invitrogen).

**NG108 Cell Culture and Peptide Identification**

NG108 cells were maintained in Dulbecco’s modified Eagle’s medium (DMEM) (Special Media, Philipsburg, NJ) and supplemented with 10% dialyzed fetal bovine serum (Invitrogen), HAT (Sigma-Aldrich, St. Louis, MO), and 100 units/mL of penicillin / streptomycin, FLAG-tagged EphB2 plasmid was transfected in NG108 cells using Superfect (Qiagen, Valencia, CA). At 48 hrs after clustered ephrin-B1 treatment (Kayser et al., 2006), cells were lysed in lysis buffer containing 1% Triton X-100, 150 mM NaCl, 20 mM Tris, pH 8.0, 0.2 mM EDTA, pH 8.0, 2 mM Na_3_VO_4_, 2 mM NaF, and protease inhibitors (Complete tablet; Roche, Mannheim, Germany).

Lysates were incubated with agarose-conjugated anti-FLAG M2 antibody (Cat# F1804, Sigma-Aldrich) overnight. After incubation, the beads were spun and washed in lysis buffer. Precipitated proteins were eluted by boiling in SDS-PAGE sample buffer. After elution sample volume was reduced by ½ by vacuum centrifugation. Samples were separated on gels (BioRad, Hercules, CA), the gels were stained with Coomassie Blue, and the EphB2 band was excised (receptor IPs). For in-gel digestions, excised gel bands were cut into small pieces and destained in 50 mM ammonium bicarbonate / 50% acetonitrile, dehydrated with acetonitrile, and dried. The gel pieces were rehydrated with 10 ng/µL trypsin solution in 50 mM ammonium bicarbonate and incubated overnight at 37 °C. Peptides were extracted twice with 5% formic acid / 50% acetonitrile followed by a final extraction with acetonitrile. After in-gel digestion, samples were dried by vacuum centrifugation. TiO_2_ tips (NuTip, 1-10 µL for affinity purified proteins and 10-100 µL for complex mixtures, Glygen Corp., Columbia, MD) were conditioned by pipetting 10 (purified protein) or 100 µL (complex mixture) of 1.0% TFA in 80% acetonitrile loading buffer through the tip 10 times. Peptides were dissolved in 10 or 100 µL of loading buffer and loaded on to the tip by pipetting peptide solution through the tip at least 10 times. The tip was then washed with an additional 10 or 100 µL of loading buffer by pipetting the solution through the tip at least 10 times. The tip was washed a second time with 10 or 100 µL of 0.1% TFA in 80% acetonitrile by pipetting the solution through the tip at least 10 times. Bound peptides were eluted by pipetting 3.5-10 or 25-100 µL of 500 mM NH_4_OH through the tip 10 times. Solvent was removed by vacuum centrifugation and stored at -20 °C until MS analysis.

Samples were resuspended in 5 µL of 0.1% formic acid in HPLC grade H_2_O and loaded onto a Symmetry 5μm particle, 180 μm x 20 mm C18 precolumn (Waters), then washed 5 min with 1% acetonitrile in 0.1% formic acid at a flow rate of 20 µL/min. After washing, peptides were eluted and passed through a BEH300 1.7 μm particle, 75 μm x 150 mm C18 analytical column (Waters, Milford, MA) with a gradient of 1-80% acetonitrile in 0.1% formic acid. The gradient was delivered over 120 min by a nanoACQUITY UPLC (Waters) at a flow rate of 250 nL/min, to a fused silica nanoelectrospray needle (New Objective, Woburn, MA). The LTQ-Orbitrap (Thermo Scientific, Waltham, MA) was operated in positive ion mode, a full scan was acquired in the Orbitrap analyzer at resolution 60,000 and at the same time MS/MS spectra of the five most-intense peptide ions were acquired in the LTQ analyzer. The lock mass (siloxane ion of m/z 445.120025) option was enabled in all full scans to improve mass accuracy of precursor ions [5]. Multistage activation was enabled in all MS/MS scans to improve fragmentation spectra of phosphopeptides and MS3 was triggered upon neutral losses of 98, 49, or 32.7 m/z (corresponding to +1, +2, +3 charge states of the loss of phosphate) if this loss was observed as one of the top 3 most intense fragment ions [6]. Raw data were processed using Bioworks (version 3.2, Thermo Scientific) or DTASupercharge (open source made available by Mathias Mann and colleagues at http://msquant.sourceforge.net/) software and peak lists were searched against concatenated forward and reversed NCBInr Mouse and Rat protein databases using the Mascot search engine (version 2.2, Matrix Science) with the following search criteria: full tryptic specificity; 1 missed cleavage; phosphorylation (S,T and Y) were included as a variable modifications; precursor mass error of 10 ppm and fragment mass tolerance of 0.5 Da. To identify the most likely sites of phosphorylation for EphB2 observed peptides, spectra identified as phosphopeptides by Mascot were processed and validated using MSQuant software (Peter Mortensen and Matthias Mann, http://msquant.sourceforge.net/). MSQuant calculated the probabilities for phosphorylation at each potential site and generated PTM scores as described previously [6].

**HEK293T Cell Culture**

HEK293T cells were maintained in DMEM (Invitrogen), 10% Fetal Bovine Serum (Hyclone, Logan, UT), penicillin-streptomycin (Invitrogen), and glutamine (Invitrogen). For transfection, HEK293T cells were plated in 6-well or 35 mm culture plates (BD Bioscience) and transfected with indicated EphB2, GluN1, or GluN2B constructs using the calcium phosphate method [3, 7, 8]. 100 μM APV (Sigma-Aldrich) was added to the culture medium after NMDAR transfection to prevent excitotoxicity.

**Primary Neuronal Culture**

Dissociated cortical neurons were prepared from embryonic day 17 (E17) to E18 Long Evans rats as previously described [1, 2]. Dissociated spinal cord neurons were prepared from E14.5 rats. Motor neurons were removed by using Optiprep solution (Nycomed Pharma) and centrifugation [9]. Neurons are plated on poly-D-lysine (BD Bioscience, Bedford, MA) and laminin (BD Biosciences) coated glass coverslips (12 mm; Bellco Glass, Vineland, NJ) in 24-well plates (Corning Life Sciences, Lowell, MA). For immunostaining and electrophysiology experiments, neurons were transfected using Lipofectamine 2000 (Invitrogen, Carlsbad, CA) as previously described [1, 2]. For biochemistry, dissociated neurons were plated at a density of 8-10 × 10^6^ cells per 100 mm diameter dishes (BD Bioscience) pre-coated with poly-D-lysine and laminin. For the transfection of each construct, the wells or dishes were randomly chosen.

**Immunoprecipitation**

Immunoprecipitations were performed as previously described with small changes [3, 10]. After treatment with clustered ephrin-B2-Fc or Fc control, cortical neuron cultures (or untreated HEK293T cells) were lysed in RIPA buffer (750 µL for 100mm dishes or 300-500 µL per well for 6-well plates) containing protease inhibitors and agitated at 4°C. Cell lysates were harvested and centrifuged at 13,000 rpm for 25 minutes to pellet cellular debris. A fraction of the resulting supernatant (100 µL for 100mm dishes or 50-75 µL per well for 6-well plates) was removed as an input control. The remaining supernatant was incubated with appropriate antibody to conjugate on ice for 2 hrs: rabbit polyclonal anti-GFP (Cat# ab290; Abcam, Cambridge MA), goat polyclonal anti-EphB2 (Cat# AF467, R&D Systems), mouse monoclonal anti-GluN1 (05-432, Millipore) or mouse monoclonal anti-FLAG (M2; Sigma-Aldrich, Cat# F7425). Antibody-bound proteins were then isolated using pre-blocked protein-G agarose beads (Invitrogen) on a rotator at 4 °C. Samples were then centrifuged and beads were washed four times in RIPA lysis buffer and two times in TBS-V. Immunoprecipitants were eluted from the agarose beads by adding boiling SDS-sample buffer and boiled at 95 °C. For *in vivo* spinal cord samples, endogenous EphB1 was immunoprecipitated with anti-EphB1 (M-19; Cat# SC-9319; Santa Cruz).

**Western Blot Analysis**

Lysates from HEK293T cells or neurons were separated using 8% SDS-polyacrylamide gels and transferred onto 0.45 µm PVDF membranes (Millipore). Immunoblots were then blocked in 5% nonfat dry milk or 2% Bovine Serum Albumin in TBS-T (150 mM NaCl, 10 mM Tris pH 8.0, 0.05% Tween-20). Indicated primary antibodies were presented in blocking solution for 2 hrs at room temperature or overnight at 4 °C: mouse monoclonal anti-GFP (1:1,000; Roche, Mannheim, Germany), mouse monoclonal anti-Dynamin (1:1,000, BD Transduction Laboratories, Cat# D25520-050, Lexington, KY), goat polyclonal anti-EphB2 (1:500; R&D Systems; Minneapolis, MN), mouse monoclonal anti-NMDAR1 (1:500; Cat# 556308, BD Pharmingen, San Diego, CA or 1:300-500; Cat# 828201; Biolegend (Formerly Covance MMS-501P)), mouse monoclonal anti-FLAG (M2; 1:2500; Cat# F7425; Sigma-Aldrich), rabbit polyclonal anti-FLAG (1:500; Cat# F7425; Sigma), goat polyclonal anti-EphB1 (M-19; 1:500; Cat# SC-9319; Santa Cruz), mouse monoclonal anti-PSD-95 (Clone K28/43; 1:2500; NeuroMab), mouse monoclonal anti-GAPDH (Clone 6C5; 1:500; Cat# MAB374; Millipore), rabbit polyclonal anti-alpha tubulin (1:10,000; Cat# ab18251; Abcam), mouse monoclonal anti-pTyr (PY99) (1:100-300; Cat# SC-7020; Santa Cruz), rabbit polyclonal anti-EphB2 (1:500) [3], rabbit polyclonal anti-EphB2 pY662 (1:1000) [3]. HRP-conjugated secondary antibodies were used at 1:20,000 in blocking solution for 1 hr (Jackson ImmunoResearch) then visualized using ECL (PerkinElmer, Waltham, MA) and autoradiography film (Kodak, Rochester, NY or TruMark Scientific, Edison, NJ). Protein band immunoreactivity was quantified using NIH Image J software. The sample size for each experiment for western blot analysis was chosen based on previous studies [3]. Statistical analysis was performed using ANOVA followed by a post hoc comparison with Fisher’s PLSD.

**Generation of a Phosphorylation-specific Antibody**

The phosphorylated peptide Ac-CKGLKAGAI-pY-VGQVRA-NH_2_ was conjugated to Keyhole Limpet Hemocynein (KLH) in position 1 (EZBiolab, Carmel, IN). This conjugated, phosphorylated peptide was injected into rabbits for polyclonal antibody production (Covance). Antibody titer was assessed using an ELISA (Covance) with a non-phosphorylated form of the injection peptide Ac-CKGLKAGAIYVGQVRA-NH2 (EZBiolab). Serum from each animal was affinity purified using SulfoLink Coupling Resin (Pierce Biotechnology, Rockford, IL) according to manufacturer’s instructions and the phosphorylated peptide without KLH conjugation. Protein concentration was calculated using a Bradford assay (BioRad, Hercules, CA). Eluates were dialyzed overnight in Slide-A-Lyzer 10K Dialysis Cassettes (Pierce Biotechnology), then aliquoted with a final concentration of 10% glycerol and stored at 4 °C.

**Inhibitors and Reagents**

K252b was obtained from Sigma-Aldrich or Alomone labs (Jerusalem, Israel). D(-)-2-Amino-5-phosphonopentanoic acid (D-APV) and ATPγS were obtained from Sigma-Aldrich. Vanadate was obtained from ICN Biomedicals (Irvine, CA). Lactacystin was obtained from Calbiochem (EMD Biosciences, San Diego, CA). Alkaline Phosphatase, Calf Intestinal (CIP) was purchased from New England BioLabs (Ipswich, MA). PD161570 was obtained from Tocris Bioscience (Ellisville, MO). Ephrin-B2-Fc and Fc control were obtained from R&D Systems and clustered using anti-Human IgG (Jackson ImmunoResearch) then used as previously described [2, 3].

**Synaptosome Preparation**

Synaptosomes were prepared as previously described[2]. Briefly, whole brains from P30 WT CD1 mice were homogenized in HEPES-buffered sucrose. Centrifugation was used to remove the nuclear fraction and generate a S1 fraction. A crude membrane (P1) fraction was generated by centrifugation of the S1 fraction. The P1 fraction was resuspended in HEPES-buffered sucrose and centrifuged again to generate a crude synaptosome. The crude synaptosome was layered onto a sucrose gradient and found at the interface of buffer and 1.2 M sucrose. The interface was diluted and centrifuged again at 230,000 X g to obtain the final pure synaptosomal (Syn) pellet.

**Immunocytochemistry**

For phospho-tyrosine surface staining, live neurons were stained for 10 min at room temperature in PBS with anti-phospho-tyrosine (PY99, Santa Cruz, 1:50), then washed and fixed with paraformaldehyde with sucrose. After washing with PBS, ephrin-B2-Fc was added for 45 min to label the ephrin-B2-binding sites. After several washes, cells were blocked with 1% ovalbumin (Sigma-Aldrich) / 0.2% cold water fish skin gelatin (Sigma-Aldrich). For surface FLAG-tagged EphB2 staining, HEK293T cells were stained for 10 min at 37 °C with anti-FLAG (M2, Sigma, 1:400), then washed, fixed as described above. For detecting colocalization via STED of EphB2 (WT, Y504E, and Y504F), GluN1, and vGlut1, neurons were fixed paraformaldehyde with sucrose at room temperature. After several washes, cells were blocked and permeabilized with 1% ovalbumin / 0.2% cold water fish skin gelatin / 0.1% saponin (Sigma-Aldrich) for 1 hr at room temperature. Then, primary antibody was incubated for 2 hr at room temperature in blocking reagents. Antibodies used were: rabbit polyclonal anti-FLAG (1:5000; Cat# F7425; Sigma), mouse anti-GluN1/NMDAR1 (1:400; Cat# 828201; Biolegend (Formerly Covance MMS-501P)), and guinea pig polyclonal anti-vGlut1 (1:5000; Cat# AB5905; Millipore). After washing with PBS, cells were incubated with secondary antibodies in blocking reagents for 45-60 min at room temperature. Cells were then washed three times in PBS before mounting using Mowiol mounting medium for STED images or Aqua-Mount (Lerner, Kalamazoo, MI) for confocal images. Cy2, Cy3, and Cy5 secondary antibodies were used for confocal images (1:250; Jackson ImmunoResearch, West Grove, PA). ATTO 425 (1:250, Rockland antibodies & assays), Alexa Fluor 594 (1:500; Jackson ImmunoResearch), and Alexa Fluor 647 (1:500; Jackson ImmunoResearch) secondary antibodies were used for STED images. The sample size for each experiment was chosen based on previous studies [1-3, 7].

**Immunohistochemistry**

Unfixed spinal tissue was frozen in OCT compound, sectioned at 30-40 μm and mounted on microscope slides (Fisher Scientific), then stored at -80 °C until further use. The slides were warmed to room temperature and then washed in 1X PBS to remove excess OCT, then immersed for 10 minutes in 4% PFA/PBS at room temperature to fix the tissue. Antigen retrieval was carried out using a modified protease treatment protocol [11]. Slides were incubated at 37 °C for 5 minutes in dH_2_O, followed by a 10 minute incubation at 37 °C in a 0.2N HCl solution containing 0.5 mg/mL pepsin protease. Following antigen retrieval, the slides were washed 3X 5 minutes in PBS and blocked overnight at 4°C in blocking solution (10% FBS+1%BSA+0.2% TX-100 in 1X PBS). The slides were then incubated overnight at 4 °C in blocking solution containing primary antibodies. Primary antibodies used were: chicken polyclonal anti-GFP (1:750, Abcam), mouse anti-NR1 (1:200; Cat# 828201; Biolegend (Formerly Covance MMS-501P)), guinea pig polyclonal anti-VGLUT2 (1:2500; Cat# AB2251; Millipore). After washing 3X 10 minutes in PBS, sections were incubated 2 hours at room temperature in blocking solution containing secondary antibodies, washed 3X 10 minutes in PBS, then mounted with Aquamount mounting medium. Secondary antibodies used were: anti-chicken conjugated to DyLight 488 (1:500; Abcam), anti-mouse conjugated to Cy3 (1:500; Jackson ImmunoResearch). Anti guinea pig conjugated to Alexa Fluor 647 (1:500; Jackson ImmunoResearch).

**Imaging and Analysis**

Images of cultured cortical neurons were obtained using Leica TCS SP5 and SP8 confocal scanning microscopy (Leica Microsystems, Wetzlar, Germany) as previously described [2, 7]. Briefly, images were acquired at 63X resolution, numerical aperture 1.4, and oil-immersion objective. For each experiment, images were taken with identical confocal settings across conditions. The region for the image acquisition in the coverslips was randomly chosen. Analysis was done using NIH ImageJ for phospho-tyrosine surface staining on ephrin-B2-binding sites. Images were collected from at least three independent experiments. For measuring the number of phospho-tyrosine -positive puncta on ephrin-B2-binding sites, co-localized puncta were defined and counted using Colocalization plug-in (http://rsbweb.nih.gov/ij/plugins/colocalization.html) with the same threshold across experimental conditions. Both image acquisition and quantification were performed by investigators who were blind to the experimental conditions. The sample size for each experiment was chosen based on previous studies.

STED images were obtained using Leica TCS SP8 and Leica TCS STED CW confocal microscope (Leica Microsystems, Wetzlar, Germany) with 100X oil immersion objective with 5–10X zoom to obtain 15–30 nm pixel sizes as previously described [12]. Analysis was done using NIH ImageJ. Images were collected from three independent experiments. Both image acquisition and quantification were performed by investigators who were blind to the experimental conditions.

Images of spinal cord sections were obtained using Leica TCS SP8 confocal scanning microscopy with 10X resolution objective. Analysis was done using ImageJ software for measuring average fluorescence intensity GluN1and VGLUT2 in superficial layers of dorsal horn where VGLUT2 staining is prominent. For the comparison of GluN1 and VGLUT2 intensity in different conditions, the amount of GluN1 or VGLUT2 intensity was normalized to the average intensity of control condition.

**Cell-surface Biotinylation**

Biotinylations were performed as previously described [2]. Briefly, cells were washed twice in ice-cold PBS-Ca^2+^/Mg^2+^ rinsing solution then cell surface proteins were bulk labeled using 1 mg/mL Sulfo-NHS-SS-Biotin (Pierce Biotechnology). Unreacted biotin was quenched using rinsing solution with 100 mM glycine. Cells were then washed and lysed using radioimmunoprecipitation assay (RIPA) buffer. Lysates were harvested and centrifuged to remove cellular debris. Biotinylated proteins were extracted using monomeric avidin agarose (Pierce Biotechnology). Surface proteins were solubilized off the avidin beads using SDS sample buffer. Equal amounts of total cell lysate and biotinylated (surface) proteins were subjected to SDS-PAGE and western blot analysis.

**Optical Stimulation and Electrophysiology**

To stimulate DIV 21-23 cultured rat cortical neurons, an optogenetic method using channelrhodopsin-2 (ChR2; a cationic channel that is activated by blue light) was adopted. DIV 14 cultured neurons were transfected with ChR2 (H134R)-mCherry under the control of the CaMKIIa promoter (Addgene 26975) or transduced with adeno-associated virus, AAV1 or AAV5 expressing ChR2 (UPenn Vector Core). Focal-excitation of ChR2 in neurons was achieved by irradiation of 470 nm light delivered through a quartz glass optical fiber that has a blunt end and was connected to LED lamp house at the other end. An applied light intensity was adjusted manually with a constant-current control unit. Recordings from DIV 21-23 cultured rat cortical neurons were performed using whole-cell patch methods as previously described [2, 7]. Briefly, coverslips were moved into a recording chamber and bathed in a HEPES-buffered artificial cerebrospinal fluid (ACSF) solution in mM: 140 NaCl, 5 KCl, 2 CaCl_2_, 1 MgCl_2_, 20 glucose, and 10 HEPES, pH 7.2). Tetrodotoxin (TTX) and picrotoxin were used at 1 and 10 μM, respectively (Sigma-Aldrich). Bicuculline was used at 50 μM, D-APV was used at 10 μM, and (αR, βS)-α-(4-hydroxyphenyl)-β-methyl-4-(phenylmethyl)-1-piperidinepropanol maleate (Ro25-6981) was used at 2.5 μM (Tocris Bioscience, Ellisville, MO). All data were collected at 5 kHz and filtered at 1 kHz; events were detected in Clampfit 9.2 (Molecular Devices, Sunnyvale, CA). Event analysis and statistics were performed using Clampfit software (Molecular Devices, Sunnyvale, CA). To isolate the NMDAR component, neurons were held at +50 mV in the presence of TTX (1 μM), bicuculline (50 μM), and picrotoxin (10 μM). The sample size for each experiment was chosen based on previous studies [2, 7].

**Behavioral testing and drug administration**

For pain studies animals across cages for an individual experiment were assigned numbers and numbers were pulled from a box to assign to groups. Groups were always spread across housing cages (e.g. no group was ever housed in a single cage). Paw withdrawal responses were measured using calibrated von Frey hairs using the up-down method at 1, 3 or 24 hrs after intrathecal (i.t.) injection of Fc control or ephrin-B2-Fc (0.2 μg/animal, n = 6); 1, 2, 3, 4 weeks after i.t. injection of lentivirus harboring EphB2 WT and Y504E; 1, 3, 24 hrs after i.t. injection of K252b (1 μg/animal that was injected with lentivirus harboring EphB2 WT or Y504E). Plantar incision was performed as described previously [13]. Paw withdrawal responses were measured 2, 4, 6 hrs after i.t. injection of K252b (1 µg) at the time of plantar incision and 1, 2, 3 hrs after second injection of K252b, 24 hrs following incision. Investigators in behavioral experiments were always blinded to the group allocation and the treatment. Investigators were not blinded when conducting data analysis for behavior experiments. The sample size (6 mice per group) for each experiment was chosen based on previous studies [14] and extensive historical data expecting an effect size of ~ 25% difference between groups. For pain studies, the only inclusion criteria was the ability to establish a baseline mechanical threshold of ~ 1.5 grams. Exclusion criteria were based on adverse reactions to viral injection or plantar incision. Any mouse showing excessive signs of discomfort was immediately excluded and euthanized.

***In vitro* kinase assay**

For the *in vitro* kinase assay, purified recombinant protein fragment with EphB2 intracellular domain (EphB2, active; Millipore) was used. The kinase assay was carried out according to manufacturer’s protocol with slight modifications. Kinase reaction was started by addition of 10 mM MnCl_2_, 10 mM MgAc and 100 µM ATP in MOPS buffer (8 mM MOPS/NaOH, pH7.0, 0.2 mM EDTA). After 30 min of incubation at 30 °C, phosphorylated proteins were separated by SDS-PAGE and analyzed by western blotting with anti-phosphotyrosine antibody (PY99).

For kinase assay using medium conditioned with ephrin-B2-treated or control cultured neurons, medium of cultured neurons was replaced with artificial cerebrospinal fluid (ACSF) to remove proteins present in regular culture medium and leaked from dead cells. Neurons were treated with ephrin-B2 or control reagents for 45-60 minutes. Conditioned ACSF were filtered to remove cell debris and mixed with 100 µM ATP, 10 mM magnesium acetate and 10 mM manganese chloride to activate kinase released from cultured neurons. Extracellular region of EphB2 fused with human IgG (EphB2-Fc) was used as the substrate. After 30 min of incubation at 37 °C, EphB2-Fc was precipitated with Protein G agarose and tyrosine phosphorylation of EphB2-Fc were detected as described above.

**Statistics**

Statistical analyses were performed using analysis of variance (ANOVA) followed by post hoc comparison with Fisher’s PLSD test, or nonparametric Kruskal-Wallis test followed by Dunn's multiple comparison post test as appropriate. Cumulative frequency of mEPSC amplitude was analyzed with Kolmogolov-Smirnov test. For statistical analysis of behavioral data non-parametric Mann-Whitney U-test or ANOVA followed by Dunnet’s post hoc comparison were used. *P* values less than 0.05 were considered significant.

**SUPPLEMENTAL REFERENCES**

1. Kayser MS, Nolt MJ, Dalva MB. EphB Receptors Couple Dendritic Filopodia Motility to Synapse Formation. Neuron. 2008:56-69. doi: 10.1016/j.neuron.2008.05.007.

2. Nolt MJ, Lin Y, Hruska M, Murphy J, Sheffler-Colins SI, Kayser MS, et al. EphB Controls NMDA Receptor Function and Synaptic Targeting in a Subunit-Specific Manner. The Journal of Neuroscience. 2011;31(14):5353-64. doi: 10.1523/JNEUROSCI.0282-11.2011.

3. Dalva MB, Takasu MA, Lin MZ, Shamah SM, Hu L, Gale NW, et al. EphB receptors interact with NMDA receptors and regulate excitatory synapse formation. Cell. 2000;103(6):945-56. doi: 10.1016/S0092-8674(00)00197-5.

4. Kayser MS, McClelland AC, Hughes EG, Dalva MB. Intracellular and Trans-Synaptic Regulation of Glutamatergic Synaptogenesis by EphB Receptors. The Journal of Neuroscience. 2006;26(47):12152-64. doi: 10.1523/jneurosci.3072-06.2006.

5. Olsen JV, de Godoy LMF, Li G, Macek B, Mortensen P, Pesch R, et al. Parts per Million Mass Accuracy on an Orbitrap Mass Spectrometer via Lock Mass Injection into a C-trap. Molecular & Cellular Proteomics. 2005;4(12):2010-21. doi: 10.1074/mcp.T500030-MCP200.

6. Olsen JV, Blagoev B, Gnad F, Macek B, Kumar C, Mortensen P, et al. Global, I*n Vivo*, and Site-Specific Phosphorylation Dynamics in Signaling Networks. Cell. 2006;127(3):635-48. doi: 10.1016/j.cell.2006.09.026.

7. McClelland AC, Sheffler-Collins SI, Kayser MS, Dalva MB. Ephrin-B1 and ephrin-B2 mediate EphB-dependent presynaptic development via syntenin-1. Proceedings of the National Academy of Sciences. 2009;106(48):20487-92. doi: 10.1073/pnas.0811862106.

8. Xia Z, Dudek H, Miranti CK, Greenberg ME. Calcium influx via the NMDA receptor induces immediate early gene transcription by a MAP kinase/ERK-dependent mechanism. The Journal of Neuroscience. 1996;16(17):5425-36.

9. Wen X, Tan W, Westergard T, Krishnamurthy K, Markandaiah SS, Shi Y, et al. Antisense Proline-Arginine RAN Dipeptides Linked to C9ORF72-ALS/FTD Form Toxic Nuclear Aggregates that Initiate *In Vitro* and *In Vivo* Neuronal Death. Neuron. 2014;84(6):1213-25. doi: 10.1016/j.neuron.2014.12.010.

10. Takasu MA, Dalva MB, Zigmond RE, Greenberg ME. Modulation of NMDA Receptor- Dependent Calcium Influx and Gene Expression Through EphB Receptors. Science. 2001;295(5554):491495. doi: 10.1126/science.1065983.

11. Fukaya M, Watanabe M. Improved immunohistochemical detection of postsynaptically located PSD-95/SAP90 protein family by protease section pretreatment: A study in the adult mouse brain. Journal of Comparative Neurology. 2000;426(4):572-86. doi: 10.1002/1096-9861(20001030)426:4<572::aid-cne6>3.0.co;2-9.

12. Hruska M, Henderson NT, Xia NL, Marchand SJ, Dalva MB. Anchoring and synaptic stability of PSD-95 is driven by ephrin-B3. Nature Neuroscience. 2015;18(11):1594-605. doi: 10.1038/nn.4140.

13. Banik RK, Woo Y, Park S, Brennan TJ. Strain and Sex Influence on Pain Sensitivity after Plantar Incision in the Mouse. Anesthesiology. 2006;105(6):1246. doi: 10.1097/00000542-200612000-00025.

14. Asiedu MN, Tillu DV, Melemedjian OK, Shy A, Sanoja R, Bodell B, et al. Spinal Protein Kinase M ζ Underlies the Maintenance Mechanism of Persistent Nociceptive Sensitization. The Journal of Neuroscience. 2011:6646-53. doi: 10.1523/JNEUROSCI.6286-10.2011.
